# Unique transposon landscapes are pervasive across *Drosophila melanogaster* genomes

**DOI:** 10.1093/nar/gkv1193

**Published:** 2015-11-17

**Authors:** Reazur Rahman, Gung-wei Chirn, Abhay Kanodia, Yuliya A. Sytnikova, Björn Brembs, Casey M. Bergman, Nelson C. Lau

**Affiliations:** 1 Department of Biology and Rosenstiel Basic Medical Science Research Center, Brandeis University, Waltham, MA 02454, USA; 2 Institute of Zoology, Universität Regensburg, Regensburg, Germany; 3 Faculty of Life Sciences, University of Manchester, Manchester M21 0RG, UK

## Abstract

To understand how transposon landscapes (TLs) vary across animal genomes, we describe a new method called the Transposon Insertion and Depletion AnaLyzer (TIDAL) and a database of >300 TLs in *Drosophila melanogaster* (TIDAL-Fly). Our analysis reveals pervasive TL diversity across cell lines and fly strains, even for identically named sub-strains from different laboratories such as the ISO1 strain used for the reference genome sequence. On average, >500 novel insertions exist in every lab strain, inbred strains of the *Drosophila* Genetic Reference Panel (DGRP), and fly isolates in the *Drosophila* Genome Nexus (DGN). A minority (<25%) of transposon families comprise the majority (>70%) of TL diversity across fly strains. A sharp contrast between insertion and depletion patterns indicates that many transposons are unique to the ISO1 reference genome sequence. Although TL diversity from fly strains reaches asymptotic limits with increasing sequencing depth, rampant TL diversity causes unsaturated detection of TLs in pools of flies. Finally, we show novel transposon insertions negatively correlate with Piwi-interacting RNA (piRNA) levels for most transposon families, except for the highly-abundant *roo* retrotransposon. Our study provides a useful resource for *Drosophila* geneticists to understand how transposons create extensive genomic diversity in fly cell lines and strains.

## INTRODUCTION

Transposons comprise major portions of nearly all sequenced animal genomes because they continue to successfully proliferate in spite of host mechanisms that suppress their activity. One conserved transposon-suppressing mechanism is the Piwi/piRNA pathway, in which germ cells produce piRNAs, small RNAs antisense to transposon sequences that target the Piwi proteins to transposon transcripts to engage silencing processes. For transposons to persist and spread, they must evade suppression mechanisms and mobilize to new genomic loci that either benefit or do not harm the fitness of the host (1). Changing copy numbers and locations of transposons within genomes can be perceived as a dynamic ‘landscape’ of transposons that can profoundly affect the architecture of the host animal genome.

To better understand how truly diverse transposon landscapes (TLs) are across broad numbers of animal genomes, we need computational tools that efficiently and accurately quantify new transposon Insertion and Deletion (InDel) events in genomic data. With short-read deep sequencing becoming commonplace, a plethora of model organism genomes is now available that enable new insights into the dynamics of TLs across individuals and populations. A prominent example of this genomics revolution is the immense trove of *Drosophila melanogaster* genomes (currently >600) that have been sequenced to high coverage, including genomes from worldwide populations, cell lines, and laboratory strains (2–14).

*Drosophila melanogaster's* compact genome and global cosmopolitan distribution makes it a prime model system for population genomics studies. As such, the majority of resequenced genomes in this species have been sampled from natural populations, including: (i) the *Drosophila* Genetic Reference Panel (DGRP), a bank of ∼192 highly-inbred strains from Raleigh, North Carolina, maintained in the Bloomington *Drosophila* Stock Center (BDSC) for Genome-Wide Association Studies of biomedical-relevant traits (8,11,14); (ii) the *Drosophila* Genome Nexus (DGN), a broad collection of genome sequences from several independent population studies of *D. melanogaster* strains isolated from Europe, the Middle East and Sub-Saharan Africa (10,12) and (iii) various pools of flies sampled from several locales in the United States, Austria, Italy, Portugal and Australia (6,7,9,13).

Since Illumina short-read sequences are currently the dominant format for these population genomics studies, most TL analyses in *D. melanogaster* entail comparing reads to the reference genome sequence of the ISO1 strain (genotype of *y^1^*; *cn^1^ bw^1^ sp^1^*) (15–18). In this genome, the large bulk of transposons is densely packed near the telomeres and in pericentromeric heterochromatin, as well as specific transposon-dense chromosomes like the Y and fourth (19–22), which are still notoriously challenging to assemble and annotate with even current genomics technologies. Although heterochromatin is generally thought to be inert (23), it serves important structural roles for chromosome maintenance (24–27), and reproduction (28). However, another ∼10% of the euchromatic genome is also filled with transposons that can reside within gene introns or near gene promoters, and thus able to influence gene expression (21,22,29–35). The most recent version of the reference *D. melanogaster* genome sequence, called Release6 (or dm6), has merged all major euchromatic and heterochromatic scaffolds into one assembly (20,36). RepeatMasker (37) annotates ∼32 750 transposon loci in this release, comprised of 135 well-characterized transposon families. These transposons can also be mainly broken down to Class 1 retrotransposons (∼70%), Class 2 DNA transposons (∼10%) and rolling-circle transposons (∼18%, *DINE-1* elements, (38)).

Several programs have been previously described for detecting *de novo* transposon InDels relative to the Release5/dm3 *D. melanogaster* genome (9,39–41), whereas other programs have been specifically developed for determining the presence and absence of reference-genome annotated transposons (42,43). These tools have been used to define preferred Target Site Duplication (TSD) sequences for transposon insertions (39,41,42), and reveal the frequent occurrence of transposons at low allele frequency at many genomic loci (40). These different programs have typically not been applied to identical datasets, however previous work found that only a small minority of transposon insertions were called in common by three programs on the same genomic sequence input (44). Thus, the best approach for determining TLs from Illumina sequences remains an unresolved problem for geneticists.

Here, we introduce a new bioinformatics pipeline that generates annotation-rich outputs of TLs for individual genome inputs called the Transposon Insertions and Depletion AnaLyzer (TIDAL). In this study, we have chosen to refer to transposon absences as ‘depletions’ rather than ‘deletions’ because in pooled or heterogeneous samples the absence of a reference transposon may not be complete. Furthermore, the absence of a reference transposon in a sample may not actually be a deletion but rather an insertion in the reference genome sequence. Our tool rapidly generates TL datasets in a format that is amenable to aggregate analyses, and tracks the ratio between the reads supporting the transposon InDel relative to the reads corresponding to the unaltered genome sequence.

Using this new tool, we have generated transposon annotations for >300 *D. melanogaster* genomes from wild populations, lab strains and cell lines, and created a website database called TIDAL-Fly (http://www.bio.brandeis.edu/laulab/Tidal_Fly/Tidal_Fly_Home.html) that displays these data in user-friendly format for simple sorting and text searching in spreadsheet programs as well as aggregate analysis with Structured Query Language (SQL). Importantly, the TLs in TIDAL-Fly were run on the latest genome assembly improvements of Release 6/dm6 (20). Furthermore, we benchmarked TIDAL against three other programs on transposon insertions using identical DGRP datasets and we conducted genomic PCR to experimentally assess TIDAL's outputs. Finally, we use TIDAL-Fly outputs to provide new insights to key genomics questions such as: how frequently and how many transposon loci differ between individual fly strains, and which transposon families are most prevalent in these differences? How do TLs differ between *D. melanogaster* cell lines to individual fly strains to pools of flies? Do fly strains from different labs with identical strain name possess similar or different TLs? How does read length and sequencing depth influence the number of discovered transposon InDels?

## MATERIALS AND METHODS

### Library construction and deep sequencing of genomic DNA from *D. melanogaster* cells and lab strains

Genomic DNA (gDNA) for the S2c1 cell line was extracted from 5 × 10^6^ cells lysed overnight in SDS lysis buffer containing 20 uM Proteinase K, and phenol/chloroform extraction. 8 ug of gDNA was then fragmented in a Bioruptor sonicator (Diagenode) with 8 cycles (20 s pulse and 90 s pause) at the ‘High’ power level. Fragmented DNA was used for library construction performed essentially as described (45). After end repair and 3′-A tailing, we performed adaptor oligo duplex ligation (duplex of PE_Tdot_common_C* and barcoded linker, Supplementary Table S2). Ligation products of size between 400 and 450 bp were agarose-gel purified and used for PCR amplification with primer oligos PE-POSTPCR_1 and PE-POSTPCR_2. Amplified libraries were gel purified and quantified on an Agilent Bioanalyzer. A single-end 150 nt sequencing run was performed on the MiSeq with the version 3 kit.

DNA for CanS sub-strains from (46) was extracted from 20 females using the Qiagen DNeasy Blood and Tissue Kit (Cat. No. 69504), with the final elution step replaced by an ethanol precipation. Input DNA was tagmented using the Nextera DNA sample preparation kit (Cat. No. FC-121-1030). Following a cleanup using the Zymo-Spin kit (Cat. No. D4023) the purified, tagmented DNA was then amplified via limited-cycle PCR which also added the indices (i7 and i5) and sequencing primers. AMPure XP beads (Cat. No. A63881) were then used to purify and size select the library DNAs. The libraries were then normalized to 2nM and pooled prior to cluster generation using a cBot instrument. The loaded flow-cell was then paired-end sequenced (2 × 101 nt) on an Illumina HiSeq2500 instrument. Demultiplexing of the output data (allowing one mismatch) was performed with bcl2fastq 1.8.4.

Genomic sequence data that we generated for this study has been deposited in the Sequencing Reads Archive (SRA) under the accession numbers SRR1983913 and ERP009394. All the other genomic DNA and small RNA datasets used in this study were downloaded from the SRA, with all accession numbers recorded in Supplementary Table S1.

### Construction of the TIDAL pipeline

The initial framework for the TIDAL pipeline was built for detecting TE insertions from single-end Illumina reads using a split-read approach (33). Here, we improved and expanded the TIDAL pipeline to also detect TE depletions from the same input of single-end reads, and automated the pipeline using a combination of shell, C and PERL scripts that follows the detailed flowchart path in Supplementary Figure S1. Source code for the TIDAL program can be found on GitHub at: https://github.com/laulabbrandeis/TIDAL.

TIDAL starts with an input file listing the library name, the SRA Run accession numbers (SRR#), and a user-defined read length reflecting the size of reads in the library. The specified SRA file is downloaded and converted to a FASTQ file with the fastq-dump command from the SRA Tool kit using the ‘–split-spot [readlength]’ parameter to convert all paired-end reads into single-end reads. In case of the DGRP libraries, one SRA accession might have several libraries of varying length reads, so only reads within 10 nt of the specified length is extracted with a custom perl script. Trimmomatic (47) was used to trim low quality bases from 5′ and 3′ end of the read and if the average quality of the read is below 20 (using parameters ‘LEADING:20 TRAILING:20 AVGQUAL:20’).The trimmed reads are then mapped to the Release6/dm6 reference genome sequence with Bowtie2 (48) using parameters ‘–sensitive –end-to-end’. The alignments are filtered with samtools (49) to retain those with MAPQ scores ≥ 10. These alignments are used for coverage analysis later in the pipeline and by Control-FREEC application (50) to generate a Copy Number Variation genome chart in PDF format (using parameters step = 5000, dm6 chr file and pre-computed 100mers GEM mappability tracks). All the unmapped reads from the reference genome mapping are aligned to *Drosophila* viruses, structural RNAs, and transposon consensus sequences curated from Repbase (51) and Flybase (20) using Bowtie1 (52) (using parameters ‘-v 3 –k 2 –m 100000’), the unmapped reads used for split read analysis. Less than 5% of the reads from the input library is left at this stage and fraction of reads culled at each step of the pipeline is recorded in a file. For TE insertions, the 22 nt long 5′ and 3′ termini of these unmappable reads were mapped with Bowtie1 to TE consensus sequences with parameters ‘-v 2 -k 5 –m 5’, and to the masked reference genome with parameters ‘-v 1 -k 5 –m 5′. Reads with one termini mapping to ≤5 TEs and one termini mapping uniquely to the genome are potential candidate reads that shows evidence of de novo TE insertion. These candidate reads are aligned to the reference genome with BLAT (53), where the BLAT alignment score reflects the fraction of bases from the read that can mapped to the reference genome. If almost the entire read maps to the reference genome then it will have a very high BLAT score and is likely to be a false positive read. These reads are then grouped into clusters where the genomic coordinates of the split reads mapping are from same strand and fall within 300 nt of each other and the other end correspond to the same TE family. We only retain clusters with at least 4 reads and cluster size that is greater than half of the read length minus 22 nt, whereas clusters in specific repeat-rich regions (not marked by RepeatMasker) are discarded. The last key validation step is to discard false-positive clusters which display an average score >83% for all of the BLAT score ratios of the full length reads within the cluster. A coverage ratio (CR) for each TE insertion is then determined for the interval of the cluster window, expanded by 22 nt at the 5′ coordinate, by dividing the number of TE insertions reads over the number of reference genome mapped reads, which are estimated by using coveragebed (54) from earlier Bowtie2 alignment to reference genome, plus a pseudocount of 1. For example, a TE insertion that has 30 TE-insertion reads and 2 reference genome mapping reads results in a CR of 10 = [30/(2 + 1)]. By using Refseq annotation tracks from UCSC genome browser track, we then annotated the identity of nearby genes for each TE insertion; and if no genes are nearby then it is annotated as intergenic.

For TE depletions, both split read termini are mapped to the masked reference genome with Bowtie1 using parameters ‘-v 3 -k 5 –m 5’. Reads are marked as candidates where both termini maps uniquely with the same orientation to the same chromosome and distance between the 22mers is greater than read length of the library. These split reads are then grouped into clusters if their genomic coordinates and orientation fall within a maximum of 300 nt of each other. Clusters with at least four reads are retained and clusters in specific repeat-rich regions (not marked by RepeatMasker) are discarded. This step generates extensive lists of depletions, for which a majority are relatively small genomic deletions of <500nt that may not correspond to any TE sequence. Since TIDAL does not determine precise breakpoints of TE depletions, we instead rely on the coordinates of the read clusters for estimate of coverage. The CRs for each of these depletion sites are determined with this formula, CR=(depletion reads)/(1 + average(RefGen_5p, RefGen_3p)), where depletion reads is the number of reads in the cluster, RefGen_5p represents the number reference genome reads at a fixed interval near 5′ breakpoint and RefGen_3p represents the number of reference genome reads at a fixed interval near 3′ break point. The interval near 5′ breakpoint is defined as the 5′ read cluster − end coordinate − 2/5*(read length) to 5′ end coordinate + 22 + 1/5*(read length). The interval near 3′ breakpoint is defined as the 3′ read cluster start coordinate − 1/5*(read length) to the 3′ start coordinate +1/5*(read length). The ‘coveragebed’ tool is used to count the number of mapped reads in these interval from the earlier reference genome alignment by Bowtie2

The genomic coordinates of each TE InDel is then searched against the RefSeq and RepeatMasker annotation tracks downloaded from the UCSC Genome Browser (55) for the Release6/dm6 genome, to assign the closest gene names and the closest TE name for depletions. If no genes are nearby, the annotation defaults to ‘intergenic, not near genes’ whereas a blank is listed for depletions that do not encompass a reference TE sequence. The depletions table is filtered to keep the entries annotated with a TE name, and the final data both from the insertion and depletion components of the pipeline is saved in BED format. The reference genome coordinates are then binned into 5kb intervals, and the counts of the TE Insertions and Depletions per 5kb bin are tabulated into the Fixed-Bin table. Finally, this fixed bin table is used by a custom R script to generate the final transposon landscape genome charts as PDFs. All text file and PDF outputs were then connected to hyperlinks to form the TIDAL-Fly database website that is hosted at: http://www.bio.brandeis.edu/laulab/Tidal_Fly/ Tidal_Fly_Home.html.

### Analyses of transposon landscapes (TLs) and small RNAs

TIDAL tables were imported into Microsoft Access and aggregate analyses were conducted with queries written in Structured Query Language (SQL). Profiles of TE families were then compiled and plotted in Microsoft Excel, tracking individual families with ≥20 InDel events while grouping the remainder in an aggregated category. The TE families were then ranked according to their frequency of InDels, and then plotted as proportions in the stacked column charts. To compare transposon InDels between different TE-prediction programs, the genomic coordinates for each InDel was first converted to Release6/dm6 with the UCSC Genome Browser LiftOver tool (55). Coordinates were then rounded to the nearest kilobase (kb) to normalize the small numerical differences in the InDel coordinates calls between the different programs. Empirically, <3% of InDels had coordinates within 1 kb of another InDel, therefore the numerical rounding strategy was sufficient to maintain unique configurations for each program's TL. Venn diagrams of overlapping TE InDel coordinates between up to five libraries were conducted in R-studio with the Vennerable package (https://r-forge.r-project.org/projects/vennerable).

The libraries for DGRP fly strains ovarian small RNAs were downloaded from the SRA with the project accession number SRP019948 (44). The only small RNA library from that project not analysed for this study was RAL-427 (SRR1572816) because no Illumina genomic sequence was available for this DGRP line (only low coverage 454-genomic sequence). Small RNA reads were quality checked by FastQC (http://http://www.bioinformatics.babraham.ac.uk/projects/fastqc/), sorted according to the barcode sequence in their 5′ adaptor, and then adaptor sequences were trimmed by FASTX-Toolkit http://hannonlab.cshl.edu/fastx_toolkit/. Structural RNAs were determined by cross-mapping to a custom database, and removed from subsequent analyses. Mapping to TEs was performed with Bowtie1 against a list of *Drosophila* consensus TE sequences obtained from the Repbase database (51) and from FlyBase (22), while virus sequences were obtained from Genbank. Up to three mismatches were allowed in the small RNA mapping to the TE consensus sequences. The basic processing pipeline is written in shell script (process-quick.sh) described in (33).

### Genomic PCR assays of transposon insertions and depletions

We arbitrarily selected 49 TE InDel candidates with CRs≥3.0 from the S2c1 cell line (15 insertions, 10 depletions) and the ISO1-BL fly strain (12 insertions, 12 depletions). We also selected 48 total TE insertions that were either predicted only by TIDAL, by the LnB program (which were also predicted by TIDAL), only by TEMP, and only by the CnT program (12 sites each). Primers flanking the candidate TE InDel were designed to initially amplify a short amplicon of between 150 and 500 bp, or a long amplicon containing the TE which can range from 800bp (a solo LTR) to >9 kb (full-length intact TE). Noting that very large amplicons can be challenging to amplify, we employed multiple control genomic DNA samples to compare with the target genomic DNA sample, reasoning that the same PCR capable of amplifying the small non-TE amplicon in control samples might simply have greatly reduced or absent amplicons in the target genomic DNA sample because of the presence of the TE. We also tested one set of primer combinations that used a reverse primer base-pairing to the TE sequence, but found such TE-pairing primers to fail more frequently or generate amplicon artifacts. The list of primer sequences are listed in Supplementary Table S2.

Since we extracted one bulk sample of genomic DNA for each *D. melanogaster* cell line (S2c1) or fly strain (ISO1-BL, RAL-362, RAL-517, and RAL-765), these single DNA samples were used throughout the genomic PCR assays to demonstrate consistency in the PCRs and primers. 50ul PCR mixes contained 10 ng of gDNA template, 0.5 uM primers, 0.3 mM dNTPs, 1× GC buffer, 1 M Betaine, and 1 unit Phusion polymerase that was added only after the reaction reached 95^o^C for a hot start protocol. Annealing temperatures ranged from 52 and 68^o^C as optimized for particular primer pairs, and 10 minute extension times were used in 35 total PCR cycles. Amplicons were electrophoresed in 1% agarose gels.

## RESULTS

### TIDAL discovers transposon insertions and depletions with a more accurate split-read approach

While attempting to apply previously developed transposon InDel programs on OSS and OSC cell genomes (33), we discovered certain pitfalls in short read mapping to the Release5/dm3 *D. melanogaster* genome and transposon consensus sequences that affected the calls and total counts of transposon insertions (9,39–41). First, many reads in nearly all Illumina-sequenced *D. melanogaster* genomic libraries contain multiple single nucleotide polymorphisms (SNPs) and short (i.e. 5–25nt) InDels when compared to the reference genome sequence. Thus, a very-short read mapping algorithm like Bowtie1 (52,56) can only map ∼70% of genomic library reads, while other short read algorithms like BWA (57) and Bowtie2 (48) that accept longer reads can map >90% of genomic reads because they can accommodate these SNPs and InDels. Second, we found better reliability and simpler interpretations of novel transposon InDel patterns by only tracking single-end reads rather than considering paired-end reads. We found paired-end reads frequently generated InDel prediction artifacts from one of the paired-ends mapping to a genomic region missed by RepeatMasker (a commonly applied algorithm for the prediction and masking of transposons in reference genomes (58)), and therefore being mis-interpreted as unique euchromatic sequence (39,40). We frequently spotted these potential artifacts in un-masked heterochromatic regions that have extreme transposon density, such as many portions within the Y and 4^th^ chromosomes, telomeric and pericentromeric regions, and contigs in ‘Chr#Het’ or ‘ChrU’ (‘Het’ for heterochromatin, ‘U’ for unknown). When these un-annotated regions were re-queried with BLAT, it was apparent that these unmasked regions were in fact repetitive sequences.

**Figure 1.**
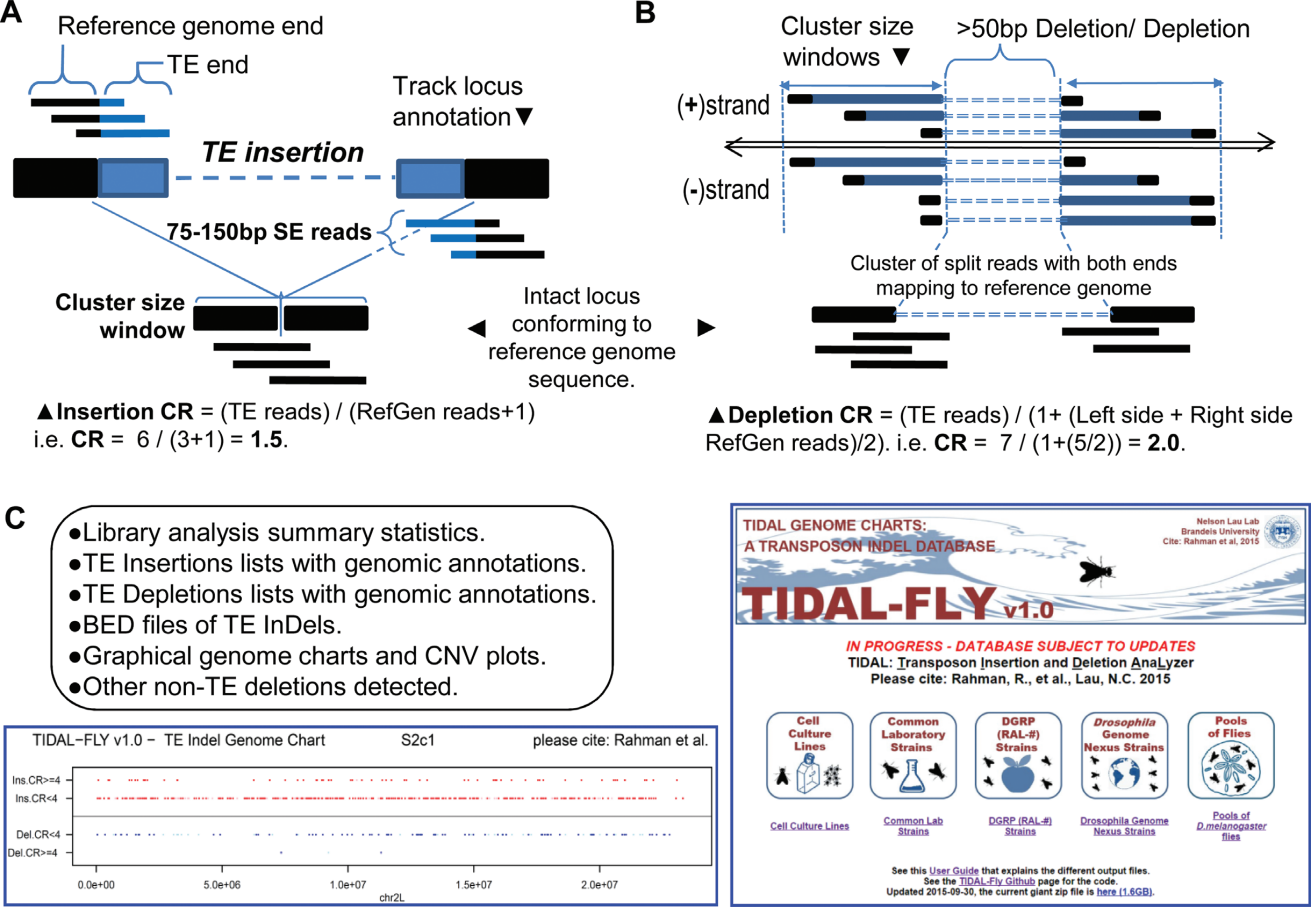
The design of the transposon insertion and depletion analyzer (TIDAL). (**A**) Diagram of the split-read approach for detecting transposon insertions, and (**B**) transposon depletions that include the calculation of a coverage ratio (CR) for each insertion and depletion. Detailed flowchart of the bioinformatics pipeline is shown in Supplementary Figure S1. (**C**) List of the output files accessible from the database, screenshot of the genome charts of transposon landscapes determined by TIDAL, and screenshot of the TIDAL-Fly database website homepage.

To overcome these obstacles, we developed a sensitive single-end, split-read transposon detection approach called TIDAL that leverages the strengths of different short read mapping tools. TIDAL first counts paired-end reads as two independent single-end reads, and culls the majority of reads which fully map to the reference genome and transposon consensus sequences. It then applies split-read mapping and validation procedures to the ‘unmappable’ reads (Figure 1A, detailed flow chart in Supplementary Figure S1). We reasoned that both transposon insertions and depletions should be accurately represented by split reads both upstream and downstream of the InDel breakpoints (Figure 1B and C). Bona fide split-reads representing the transposon InDel are then clustered together such that the genomic interval containing a cluster of split reads does not exceed a sequence window of 300 nucleotide (nt) (twice the length of the longest150 nt reads. This cluster also defines a clear window for counting reads representing the reference genome allele unaltered by a transposon InDel. By dividing the count of transposon InDel split-reads by the count of unaltered reference genome reads plus a pseudo-count of 1 in the interval defined by a cluster, we derived a relative metric called the CR, which approximates the frequency of the transposon InDel allele versus the reference genome allele of the same locus in the library. The importance of the CR is discussed further below in genomic PCR experiments and when comparing transposon landscapes between individual fly strains to pools of flies.

A second key improvement in TIDAL is the use of the BLAT algorithm (53) to validate that candidate reads are truly a split between a transposon-mapping end and a euchromatic genome end. BLAT is a sequence alignment program that yields a score which scales with different read lengths, and this score allowed us to address those genomic regions that were clearly repetitive yet were missed by RepeatMasker. Since a bona fide candidate split read cannot map to the genome in its entirety, we only retained reads whose ratios of the BLAT score to read length were ≤83%. This empirical threshold was found to be effective at clearing 92% of false positive reads such as degenerated transposon reads which can still map to multiple loci with BLAT. BLAT is slower compared to highly efficient short read aligners, so we perform BLAT alignments only for the relatively short list of candidate split reads first identified by Bowtie1 (Figure 1A). The importance of the BLAT algorithm is discussed further below when TIDAL is benchmarked against other transposon insertion algorithms applied to DGRP fly strains.

A final major improvement in TIDAL is generation of over 300 TLs based upon the latest *D. melanogaster* genome build, Release6/dm6 (see Supplementary Table S1 for accession numbers). Supplementary Figure S2 shows substantial changes in the genome build structure between the latest Release6/dm6 build and the previous Release5/dm3 build for which the bulk of modEncode (2,3) and previous transposon insertion programs were based upon (9,39–41). TIDAL outputs are accessible from a website database located at: http://www.bio.brandeis.edu/laulab/Tidal_Fly/ Tidal_Fly_Home.html and will be maintained in future phases to incorporate additional *D. melanogaster* genomes (4,5). The outputs in TIDAL-Fly are grouped according to cell lines, common lab wild-type reference strains, DGRP flies, DGN flies and pools of flies from population genetics studies (Figure 1C).

### Retrotransposons make up the bulk of TL diversity amongst *D. melanogaster* cell lines

**Figure 2.**
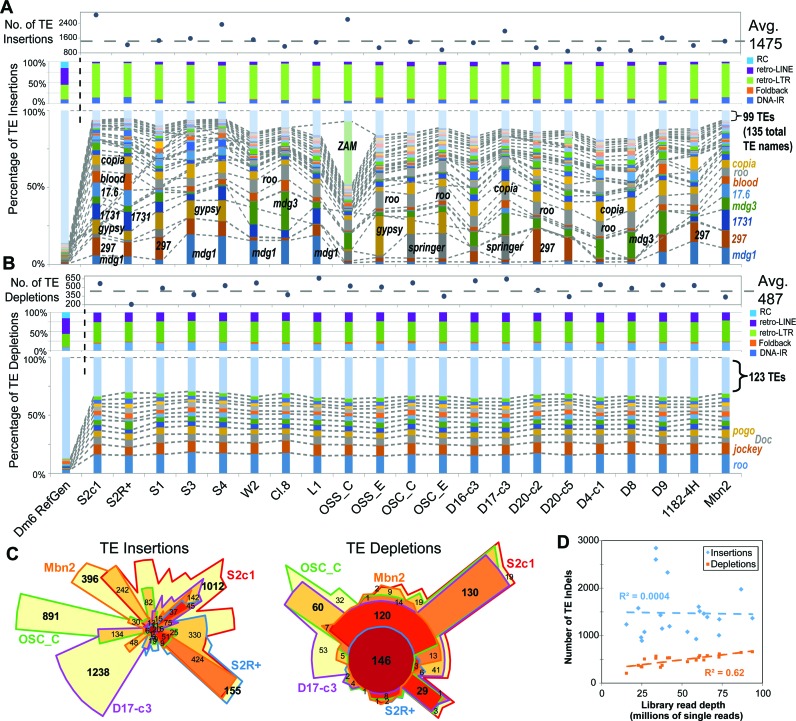
Transposon landscapes of *D. melanogaster* cell lines. (**A**) Profiles of transposon insertions, and (**B**) transposon depletions for 21 cell lines, grouped by their annotated strain origin (Supplementary Table S1A). The top panels show the total counts of transposon InDels, the middle panels show the proportions of each transposon class, and the bottom panels show the proportions of the TE families with ≥20 InDels (the rest are grouped together at the top). The dashed lines and labels in the bottom panels mark notable TE families. (**C**) Euler plots (area-proportional Venn diagrams) comparing between five representative cell lines the overlap of shared TE InDels based on their genomic coordinates. Bold numbers highlight the notably large numbers of cell-line specific TE insertions and broadly shared TE depletions. (**D**) Scatterplot comparing cell lines library read depth to TE InDels numbers.

We applied TIDAL to 21 *D. melanogaster* cell line genomes, one of which is new to this study (S2c1), some of which we and others had sequenced (33,34), and other lines from modEncode (2). Between ∼800 to ∼3000 novel transposable element (TE) insertions could be detected across cell lines, with LTR-retrotransposons making up the bulk of these new insertions, as they do in the reference genome (22) (Figure 2A). The composition of these LTR-retrotransposon insertions varied widely between cell lines, ranging from abundant *mdg1* insertions in lines originating from Oregon-R embryos (S3, S4, W2, Clone.8 and L1), *297* in several other lines, *gypsy* and *springer* in OSC lines, and an explosion of *ZAM* in one OSS line that was previously observed (33). In contrast, TE depletions were much more similar between cell line genomes, averaging at ∼480 transposons that included Class I (retrotransposons) and Class II (DNA-cut-and-paste) transposons, with very consistent patterns of the same types of transposons being depleted (Figure 2B).

When comparing the genomic coordinates of TE insertions between cell lines, gratifyingly two S2 cell variants, S2c1 (this study) and S2R+ (from modENCODE (2)) shared the greatest proportions of the same TE insertions, whereas the other cell lines tended to have cell-line-specific TE insertions (Figure 2C). These cell-line-specific insertions may represent transposition events that occurred either in cell culture or in the original fly strains used to generate these cell lines. In contrast, the sites of TE depletions at the locus level also tended to show greater similarity between cell lines. The number of TE depletions discovered by TIDAL scaled with library read depth, but no correlation existed between the number of TE insertions and read depth (Figure 2D), perhaps reflecting the extensive copy number variation amongst various loci in cell lines (2) that could contribute to major fluctuations in TE insertion number. TE insertions in exons, which would likely disrupt protein function, are rare as they are in the reference genome (35) but have a higher than expected proportion in introns (Supplementary Figure S2B).

To evaluate the efficacy of TIDAL predictions with experimental validations, we focused on TE InDels for the S2c1 cell line because we had enough genomic DNA remaining from initial library construction to also conduct genomic PCR on this sample. Our previous empirical studies showed that even a single primer designed for one end of a TE could lead to multiple amplicons that obfuscated the genomic PCR analyses (33). Therefore, we optimized a long-amplicon genomic PCR protocol using only primers mapped to the euchromatic genome and directly flanking the TE InDel. In addition to S2c1 cell genomic DNA, we included PCR tests of genomic DNA from ISO1 obtained from the BDSC (ISO1-BL) and RAL-362 fly strains, which were predicted to lack the TE InDels; these provided a set of controls for interpreting some results that were limited by inherent challenges with amplifying long multi-kilobase genomic amplicons (Supplementary Figure S3). In genomic DNA mixtures, shorter amplicons lacking the TE insertion will preferentially amplify over the rarer, longer amplicons in the PCR. Therefore, we also considered additional proxies for an insertion such as some large amplicons unable to electrophorese into the gel, and significant decreases of the short amplicons due to the insertion being too large to be amplified but still reflecting a reduced amount of short amplicon template. We arbitrarily selected 15 insertion and 10 depletion sites predicted by TIDAL with CR>3 in the S2c1 genomes, and ∼66–70% of these predictions could be validated by the genomic PCR analyses. These data are in line with our previous estimate of an empirical false discovery rate of <12% from earlier comparisons of TIDAL outputs to PCR assays with genomic DNA from OSS and OSC cells (33). We attribute the differences between the current and previous analyses to the small sample sizes of PCR studies and other undetermined genomic DNA variations between the different cell lines.

### Extensive TL diversity in common laboratory *D. melanogaster* strains

**Figure 3.**
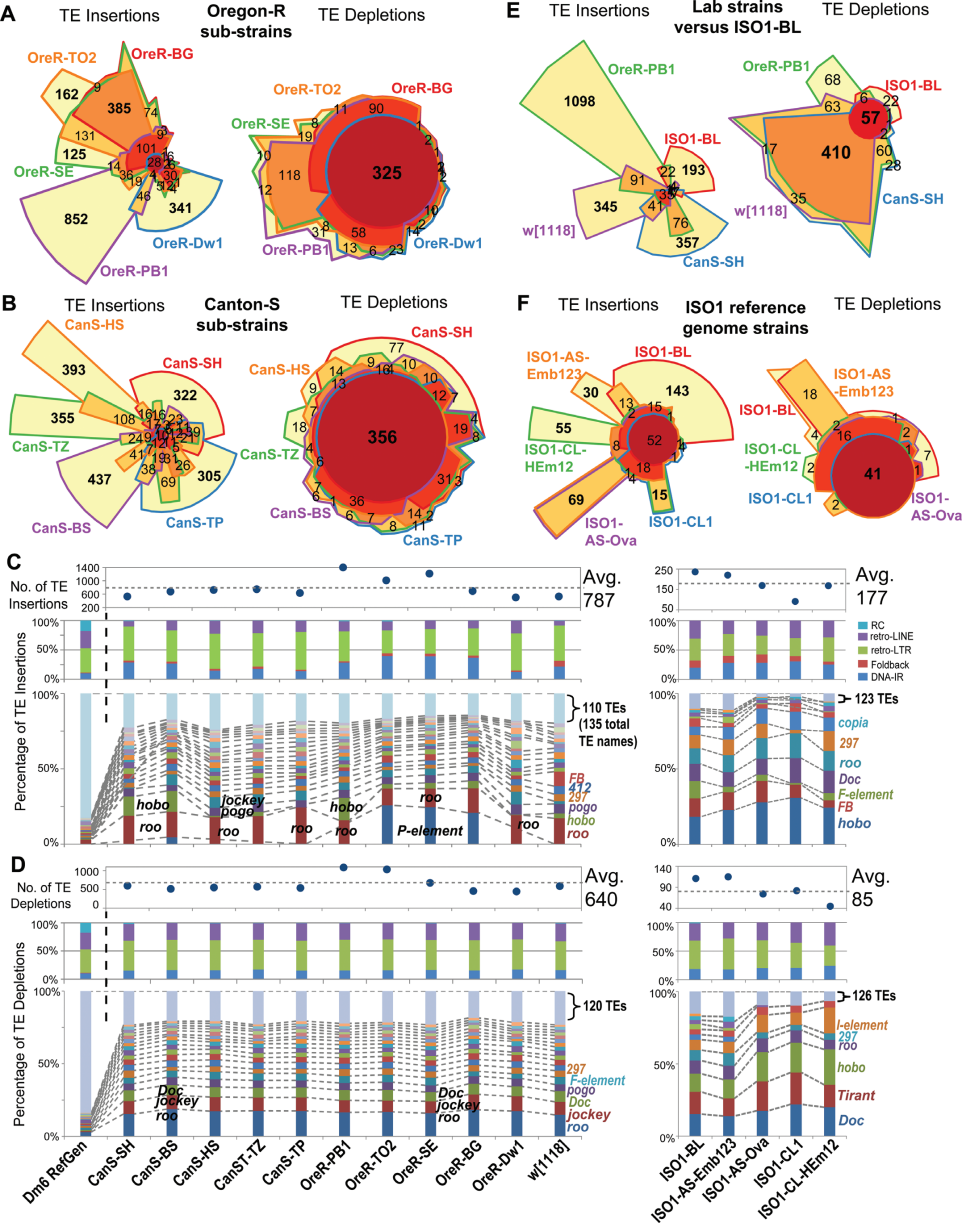
Transposon landscapes of common *D. melanogaster* lab strains. Euler plots comparing overlapping transposon InDels detected in Oregon-R (OreR) sub-strains (**A**) and Canton-S (CanS) sub-strains (**B**) from five different laboratories. Bold numbers highlight the notably large numbers of sub-strain-specific TE insertions and broadly shared TE depletions. Additional analyses of the CanS sub-strains are in Supplementary Figure S4. Profiles of transposon (**C**) insertions and (**D**) depletions for 11 lab strains (left set of panels) and five genomes from three sub-strains of the ISO1 strain (right panels, -BL: Bloomington, -AS: Spradling lab, -CL: Langley lab). In (**C**) and (**D**), the top panels show the total counts of transposon InDels, the middle panels show the proportions of each transposon class, and the bottom panels show the proportions of the TE families with ≥20 InDels (the rest are grouped together at the top). The dashed lines and labels in the bottom panels mark notable TE families. Euler plots comparing overlapping transposon InDels between the ISO1-BL strain and other lab strains (**E**) and other sub-strains of ISO1 (**F**). Bold numbers highlight the notably large numbers of strain-specific and sub-strain-specific TE insertions compared to broadly shared TE depletions. Additional analyses of the ISO1 sub-strains are in Supplementary Figure S5.

Next, we applied TIDAL to the genomes of sets of common laboratory ‘wild-type’ strains like Oregon-R (OreR), sequenced as part of modEncode and other projects (3,59), and Canton-S (CanS), used in a recent behavioral study (46) and from isogenized lines (60). We compared the TLs between five different OreR and six different CanS sub-strains isolated from different labs (Figure 3A, B), and discovered very different TLs among sub-strains of lab lines labeled with the same strain name. Whereas the OreR from TO2, SE, and BG labs shared many TE insertions, the OreR from PB1 and Dw1 had markedly distinct TE insertions. Likewise, all six CanS sub-strains also exhibited their own distinct TE insertion patterns. Differences among CanS sub-strains is not simply a reflection of TE InDels with low CR values, because a comparison of InDels with CR ≥3 still indicated highly distinct TLs (Supplementary Figure S4A-D). Most of the TE insertion differences between CanS sub-strains were represented by the *roo* retrotransposon (Figure 3C). However, in some of the OreR sub-strains, the *P-element* was a major factor in the TL differences. This observation suggests introgression of *P-element*-containing lab strains into certain stocks of OreR, which originally should be free of *P-elements* (61). Similar to cell lines, novel TE insertions in lab fly strains rarely occur in exons, but are overrepresented introns and intergenic regions (Supplementary Figure S4E), which are genomic regions that can still affect gene expression in *D. melanogaster* (33,34).

In contrast to the diversity in TE insertion landscapes, the TE depletion landscapes amongst the lab strains was highly similar both in the genomic coordinates and TE family (Figure 3A, B and D). Similar to the greater homogeneity in TE depletion patterns amongst cell lines, we interpret these depletions as representing TE insertions in the ISO1 strain that was used for *D. melanogaster* reference genome sequence. Surprisingly, TIDAL analysis of genomic data from ISO1-BL, a recently sequenced isolate of the ISO1 strain from the BDSC (18), revealed an additional 236 and 111 novel TE insertions and depletions, respectively, with regards to the reference genome (Figure 3C and D). The number of TE InDels in ISO1-BL relative to the ISO1 reference genome is lower than other lab strains (Figure 3E), supporting its close relationship to the original isolate of ISO1 used for genome sequencing (15,16). We were able to validate several of these TE InDels with genomic PCR analyses using DNA from ISO1-BL compared to the ISO1 sub-strain from the Celniker lab (ISO1-UC) that was used for the reference genome (Supplementary Figure S3C and D).

Other ISO1 sub-strains from the Spradling (62) and Langley (63) labs also displayed ∼100–300 novel TE insertions (Figure 3F, Supplementary Figure S5), with each ISO1 sub-strain appearing to have its own unique TE landscape (Supplementary Figure S5A and B). In the Spradling lab samples, there were potential signatures of tissue-specific TLs (Supplementary Figure S5C). The most commonly mobilizing TE family in ISO1 sub-strains was *hobo* (Supplementary Figure S5D and E), supporting previous results that *hobo* is unstable in ISO1 sub-strains (64,65). These data indicate that TLs are much more diverse amongst common lab fly strains than previously appreciated, even for sub-strains of the ISO1 strain used for the *D. melanogaster* reference genome sequence. These results add additional considerations to previous studies using piRNA-seq and ChIP-seq to study transposon control (66–68), since it cannot be assumed that the TL of a lab strain is the same as the reference genome.

Several of the CanS sub-strains and the ISO1-BL strain contained many individual Illumina-sequencing runs that we initially combined into single libraries for TIDAL analysis. We analyzed different subsets of these runs as ‘technical replicates’ in order to conduct an internally-controlled computational experiment that evaluates TIDAL's performance as a function of sequencing depth. In both CanS and ISO1-BL libraries, there is a linear increase in computation processing time with increasing numbers of reads (Supplementary Figure S4F, S5F, S5G), with TIDAL run times ranging from ∼100 min/∼25M reads to ∼330 min/∼100M reads. The whole-genome alignments by Bowtie2 are the most computationally intensive steps in the TIDAL pipeline. Final TIDAL outputs as displayed on the TIDAL-Fly database are only <50MB, however the intermediate BAM and FASTQ files can put disk usage at ∼5–50GB per library. TIDAL consistently detected more TE insertions than TE depletions amongst both CanS and ISO1-BL individual Illumina-sequencing runs, reaching an asymptote of fewer new TE InDels with ever increasing library depth (Supplementary Figure S4F, S5F, S5G). These analyses suggest that for lab-maintained fly stocks, there are diminishing returns in TE InDel discovery using TIDAL for genomic sequencing beyond 100M reads, as most TL patterns are already well defined at this depth.

### Benchmarking TIDAL on DGRP strain genomes

**Figure 4.**
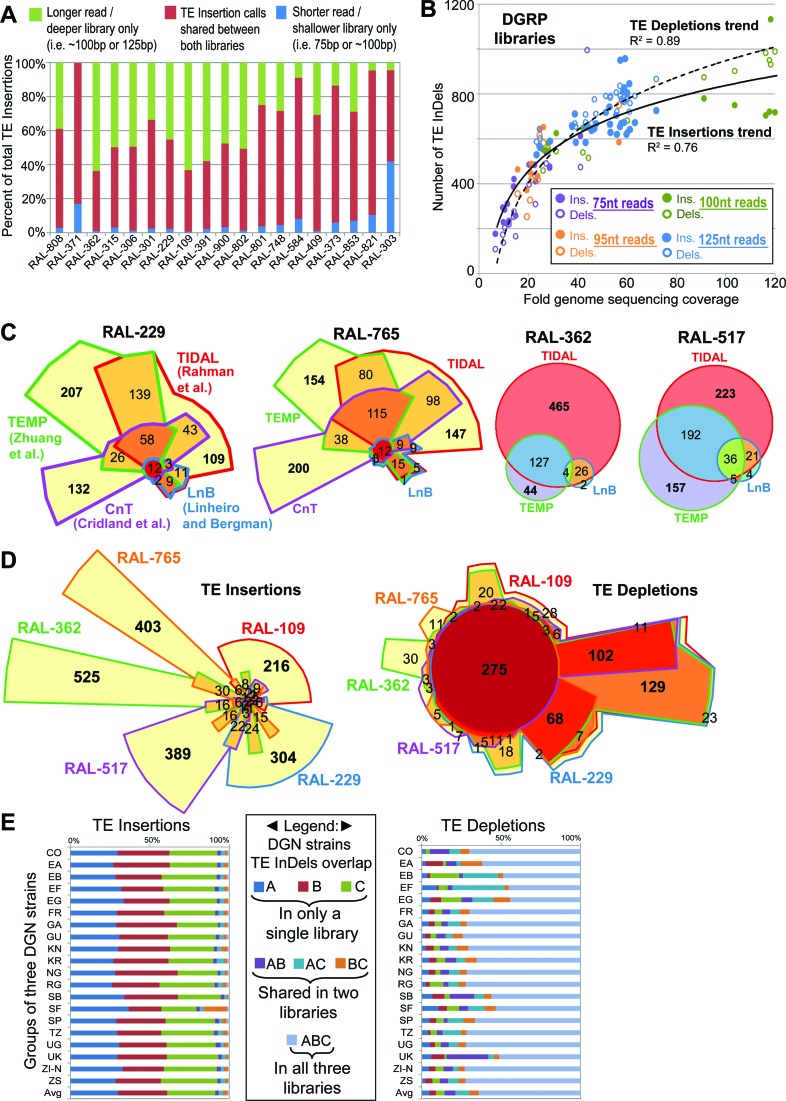
TIDAL analyses of DGRP and DGN fly strains. (**A**) Significant overlap in TE Insertions called by TIDAL for 19 DGRP strains that were sequenced twice by different Illumina genomic libraries of different lengths and depths. (**B**) Scatterplot comparing the fold genome sequencing coverage (read length*depth/genome size) to the number of TE InDels for 68 libraries from 23 DGRP strains. Logarithmic trend lines were fitted to the TE InDel points. (**C**) Euler plots comparing the genomic locus overlap in transposon insertion calls made by TIDAL and three other transposon insertion programs for four DGRP strains. The CnT program lacked any insertion predictions for RAL-362 and RAL-517. Bold numbers highlight the notably large numbers of program-specific TE insertion calls. (**D**) Euler plots comparing the TE InDels called by TIDAL for five DGRP strains. Bold numbers highlight the notably large numbers of strain-specific TE insertions and broadly shared TE depletions. (**E**) Comparisons of TE InDel calls within cohorts of three strains per geographic isolates of flies from the DGN (see Supplementary Table S1D). Across multiple geographic isolates, the vast majority of TE insertions are unique to a single strain within each 3-strain cohort, whereas TE depletions are broadly shared across DGN fly strains.

Most *D. melanogaster* genomic sequence libraries consist of a single read length. However, many DGRP strains have sequencing runs with different paired-end read lengths (i.e. 75nt, 95nt, 100nt, and 125nt long reads) that have been consolidated under single SRA entries (8,11). Since TIDAL's split-read approach requires a minimum of 50nt reads for TE InDel discovery, we asked whether read lengths impacted TE InDel determination. These DGRP libraries with multiple sequencing runs of different read lengths provided an excellent opportunity to assess this question in an internally-controlled experiment. Gratifyingly, the majority of TIDAL TE insertions calls in the shorter read library were identically represented in the longer read library for the same DGRP strain, with many new TE insertions only called in the longer-read library, which also tended to be sequenced at greater depth (Figure 4A). This result confirmed the reproducibility of the TIDAL outputs and the independent deep-sequencing runs. By plotting the total number of TE InDels as a function of sequencing coverage (read length*depth/genome size) across these DGRP libraries, we also observed a logarithmic trend in TE InDel discovery with greater genome sequencing coverage that was consistent with the lab fly strains analyses (Figure 4B). However, unlike cell lines or lab strains, the capacity to detect TE depletions in DGRP fly libraries was similar to the detection of TE insertions. By classifying the data points according to read length, we detect a trend suggesting library depth may have a greater influence on TE InDel detection sensitivity than read length (Figure 4B).

Song *et al*. (44) previously showed for a single DGRP strain (RAL-391) that there was relatively little agreement between three TE insertion prediction programs called: TEMP (39), a program by Cridland *et al*. (40), and a program by Linheiro and Bergman (41) (we refer to the two latter programs as CnT and LnB, respectively). We extended this observation by analyzing several DGRP libraries to benchmark TIDAL's TE insertion calls against the calls made by TEMP, CnT, and LnB programs. Because these programs had outputs of Release5/dm3 genomic coordinates for TE insertions, we first converted their predictions to 1-kb binned Release6/dm6 coordinates for comparison. We conducted overlap analysis on the TE insertion genomic coordinates, and observed a very low number of TE insertions called by all four programs (i.e. <∼30 common calls out of >∼1000 TE insertions) (Figure 4C and Supplementary Figure S6).

Indeed, a substantial number of common TE insertions calls were shared by only two programs (Figure 4C and Supplementary Figure S6A), and significant numbers of TE insertions were uniquely called by TIDAL, TEMP and CnT. The LnB program always had far fewer TE insertion calls compared to TIDAL, TEMP and CnT, because it was designed to optimize specificity over sensitivity (41). TIDAL consistently displayed the best overlap in common TE insertion calls with the LnB split-read method, more so than CnT and TEMP, which both use BWA as its main read-mapping algorithm and paired-end information. We found more commonality in TE depletion calls between TIDAL to TEMP than for TE insertion calls (Supplementary Figure S6A). Neither the LnB nor CnT program call TE depletions.

To better understand why so many TE insertion calls in DGRP lines differed between the programs, we noticed that many TEMP and CnT output coordinates were in heterochromatic regions that are already dense with TEs, such as Chr4, ChrY, Chr2RHet and ChrU (Supplementary Figure S2, S6B), and these can be removed without affecting the overlapping calls between these programs and TIDAL (Supplementary Figure S6C). Closer inspection in the genome browser indicate that these heterochromatic regions are often not masked by RepeatMasker, yet TIDAL and LnB can avoid these regions because the pure split-read and BLAT approach can remove these reads from too high a BLAT mapping score. For 48 arbitrarily-selected sites with less-obvious reasons for discrepancy, we used PCR to evaluate TE insertion calls for the RAL-765 strains that were predicted only by TIDAL, by LnB (which overlapped with TIDAL calls), only by TEMP, and only by CnT (Supplementary Figure S7). Using the same criteria in the genomic PCR assay for the S2c1 cell line and ISO1 sub-strain data above, the number of PCR events supporting a TE insertion was approximately the same for TIDAL-only predictions (7/12) compared to TEMP-only (5/12) and CnT-only (6/12) predictions, while the agreement between TIDAL and LnB predictions was strongly supported very frequent detection of insertions by PCR (11/12). These data show that while no single method yet can identify all TE insertions in *D. melanogaster* genomes, the majority of TIDAL predictions are likely to be real insertions.

### Widespread TE insertion diversity amongst wild fly strains across the globe

To address the question of whether the TL diversity observed in cell lines and lab strains was particularly large or small compared to naturally wild fly strains, we applied TIDAL to sets of *D. melanogaster* genomes in the DGRP, DGN, and pools of flies. For the first phase of this project, we analyzed the TLs in 57 and 70 strains in the DGRP and DGN resources, respectively, and 52 different pools of flies. We chose these particular strain libraries for general qualities such as high library read depth and widespread global distributions (Supplementary Table S1), and assumed these initial subsets would represent the greater trends of TLs that would apply to the rest of the strains.

We conducted overlap analyses of the TE InDel genome coordinates between DGRP fly strains to assess the similarity of TLs. A representative Euler diagram comparing five DGRP strains shows that the overwhelming majority of novel euchromatic TE insertions are unique to each strain (Figure 4D). In contrast, the TE depletions are frequently shared between DGRP strains. To examine these TL patterns globally, we conducted overlap analyses of DGN strains by comparing cohorts of three strains each from various geographically disparate regions. Similar to the DGRP flies, all the DGN 3-strain cohorts from the same region exhibited TE insertion patterns that were frequently unique to each strain, whereas TE depletion patterns were more frequently shared between all three strains (Figure 4E). These TL depletion patterns in DGRP and DGN fly strains are remarkably consistent with lab fly strains and even cell lines, reinforcing the notion that these actually represent TE insertions in the ISO1 reference genome sequence. Although there are a few individual fly strains with unique depletion patterns, commonality of the TE depletion patterns is the prevailing picture at all of the individual genomic sites (Figure 4E) or TE family level (Supplementary Figure S8). Thus, until we have additional *D. melanogaster* reference genome sequences as a basis for comparison, we believe the current TE depletion analyses cannot be accurately used to consider TE absence differences between genomes.

### A minority of families make up the majority of TE insertion diversity

**Figure 5.**
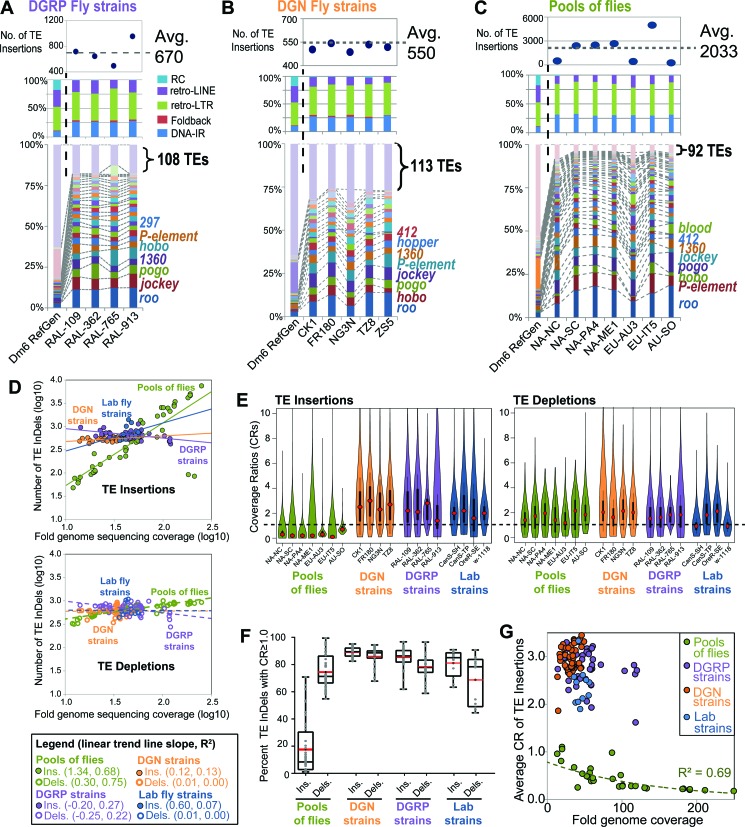
DGRP and DGN fly strains have distinct transposon landscape characteristics compared to pools of flies. Abridged profiles of transposon insertions for (**A**) DGRP fly strains, (**B**) DGN fly strains, and (**C**) Pools of flies. Detailed transposon insertion profiles for all analyzed libraries are shown in Supplementary Figure S9. The top panels show the total counts of transposon insertions, the middle panels show the proportions of each transposon class, and the bottom panels show the proportions of the TE families with ≥20 insertions (the rest are grouped together at the top). The dashed lines and labels in the bottom panels mark notable TE families. (**D**) Scatterplots comparing the fold genome sequencing coverage (reads*read length/genome size) to the number of TE InDels for the *D. melanogaster* libraries analyzed by TIDAL for this study. Linear trend lines were fitted to each group of insertions (top graph, solid lines) and depletions (bottom graph, dashed lines), with the slopes and R^2^ values for each trend line listed in parenthesis in the legend. (**E**) Violin plots of the distribution of CRs for TE insertions and depletions from select sets of fly libraries. (**F**) Box plot of the distributions of percentages of TE InDels with CR≥1.0 for each group of fly strains and pools of flies. (**G**) Scatterplots of the same data points in the box plot, but plotted against the fold genome coverage to show a unique exponential decay trend for the CR for TE insertions from pools of flies.

As a result of limited insight that can be gained by examining TE depletion patterns, we decided to focus our remaining attention to the TE insertion diversity amongst DGRP and DGN fly strains and pools of flies. Abridged plots (Figure 5A–C) that are representative of broader comparisons between strains and pools of flies (Supplementary Figure S9) reveal three new insights into *D. melanogaster* TL patterns: (i) The average number of novel euchromatic TE insertions in wild fly strains (∼550–670) from the DGRP and DGN is similar but somewhat lower than the average insertions inbred lab strains like OreR and CanS (∼750) and much lower than cell lines (>1400); (ii) compared to their ∼10% proportion of the total TEs in the reference genome (22), insertions of Class 2 DNA transposons are proportionally more abundant in both lab and wild fly strains (≥25%) relative to the Class 1 retrotransposons, which dominate in cell lines; and 3) in cell lines, lab and wild strains, a minor proportion (∼16–27%) of all characterized *D. melanogaster* TE families make up the bulk of the TE insertion diversity (≥75%).

Interestingly, the average number of TE insertions in pools of flies was frequently much greater (>2000, Figure 5C) than samples from DGRP, DGN and lab fly strains. We investigated this further by plotting the log10-transfrormed numbers of TE InDels relative to the library sequencing depth (fold genomic coverage, Figure 5D). Linear trends were fitted to each group of libraries, and the slopes for the DGRP, DGN and lab fly strains trend lines for TE InDels were all <1, indicative of the diminishing returns of TE InDel discovery with increasing read depths. However, the trend line for TE insertions in pools of flies is much higher (1.34) than the individual fly strains (0.12–0.6), suggesting that in pools of flies there is no obvious limit to discovering more TE insertions with greater depth in sequencing. This result is consistent with TE insertion landscapes varying widely among individual flies, which when pooled, lends to greater TE insertion landscape diversity in genomic libraries relative to individual strains.

If TE insertion landscape diversity is widespread between individual flies, we would predict that TE insertion reads in pools of flies would be diluted because a sample of individuals with many rare TE insertions would contribute more reads representing the unaltered reference genome sequence at any given loci. The CR for each TE InDel provides this relative measurement, and indeed the vast majority of TE insertions in pools of flies have CR <1.0, whereas the majority of TE InDels in individual fly strains have CR ≥1.0 (Figure 5E and F). In fact, the mean CR for TE insertions across the different pools of flies is 0.45 (*N* = 32), whereas the mean CR for DGRP and DGN lines is 2.98 (*N* = 46) and 2.73 (*N* = 44), respectively. The difference in CR values between pools of flies and fly strains was only apparent in TE insertions but not TE depletions (Figure 5E), reinforcing the interpretation that TE depletions are unlikely to be absences from a single strain but rather insertions that are unique to the ISO1 reference. Additionally, TE insertion CRs in pools of flies follow an exponential decay trend with greater sequencing depth (Figure 5G), which we interpret as the effect of progressively diluting the representation of each TE insertion during deeper sampling of genomic diversity in pools of flies.

### Abundance of TE families besides *roo* negatively correlates with piRNA expression

**Figure 6.**
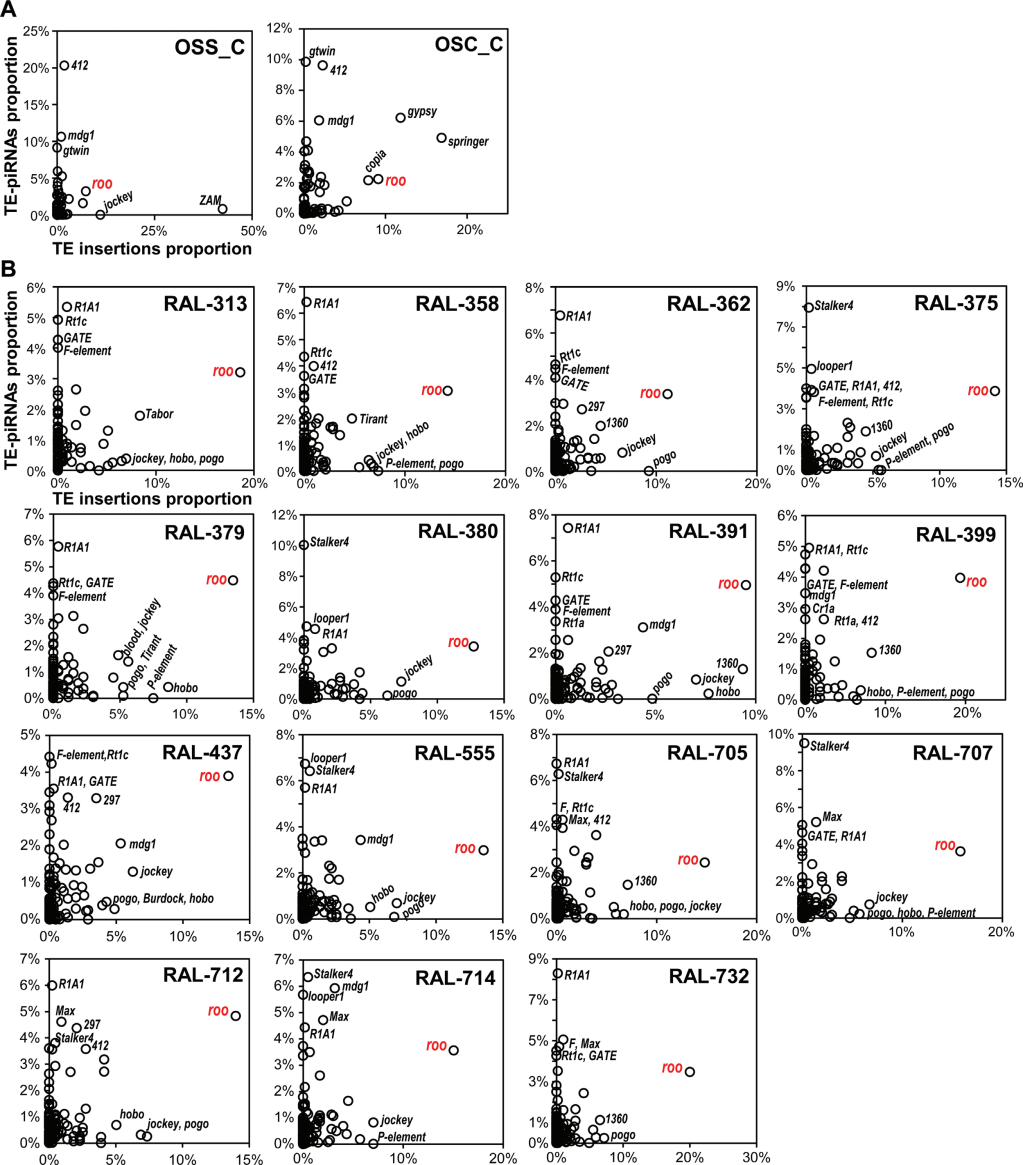
Comparisons of TE-directed piRNAs versus TE Insertions for OSS/OSC cells and DGRP fly strains. Scatterplots compare the relative proportions of TE-directed piRNAs and TE insertions for each sample in (**A**) OSS and OSC cells and (**B**) DGRP fly strains with sequenced piRNAs and genomes sequenced by Illumina. The *roo* transposon is highlighted for its exceptionally high proportion of strain-specific insertions despite the high proportions of *roo*-directed piRNAs.

Our study confirms earlier findings that TE insertion patterns vary widely among *D. melanogaster* strains (30,65,69), but our TIDAL-Fly data also indicates that only a small subset of TE families make up the majority of this *D. melanogaster* TE insertion landscape diversity. How does this TE insertion diversity relate to host TE suppression mechanisms such as the Piwi/piRNA pathway? A previous study sequenced piRNA libraries from 16 DGRP strains, but their analysis, which relied on earlier predictions of TE insertions, was unable to detect a significant correlation across strains between piRNA levels and the number of novel TE insertions for any TE family (44). We re-analyzed these and other previously published piRNA datasets (33,44) with a different approach that compared normalized proportions of piRNA counts for all TE families to the proportions of novel TE insertions predicted by TIDAL within a given cell line or strain. By measuring all piRNAs mapped directly to TE consensus sequences without normalization to the reference genome sequence, we were then able to correlate these proportions of TE-directed piRNAs with the proportions of TE insertions across all TE families within each *D. melanogaster* OSS/OSC cell lines (Figure 6A) and DGRP strain (Figure 6B). This approach quite effectively normalizes the variation in absolute counts of piRNAs and TE insertions, which can differ greatly between different small RNA and genomic DNA libraries.

In both *D. melanogaster* cell lines that express piRNAs and in 15 DGRP strains (1 strain lacked Illumina genomic sequences), the TE families with the greatest proportions of TE-directed piRNAs were in the lowest proportions of novel TE insertions (Figure 6). This relationship is consistent with the mechanism that abundant Piwi/piRNA complexes engage TE transcripts to direct silencing (33,34,70–72). Accordingly, many TE families with large proportions of insertions tended to have lower proportions of piRNAs directed at these families. However, in all the DGRP strains, the *roo* retrotransposon stands out as the sole TE family with large numbers of novel insertions and high proportions of piRNAs directed against it. Although we do not have a mechanistic explanation for *roo's* exceptionally high number of new insertions and high levels of *roo* piRNAs in DGRP flies, this result may be related to *roo's* high transposition rate (73,74) and possible horizontal transfer of *roo* between different Drosophilid species (75).

## DISCUSSION

We report a new tool optimized for accurate determination of *D. melanogaster* TLs and demonstrate the TIDAL-Fly database's value as resource for the genome-wide analysis of TLs in *D. melanogaster*. In addition to discovering how different genomic library characteristics can impact TLs, our analysis has also uncovered notable biological and genomic insights regarding TLs in the *D. melanogaster* genome. Cell lines differ widely in their TLs, with transposon insertion numbers showing weak correlation with sequencing depth (Figure 2D), perhaps reflecting the frequent aneuploidy and/or polyploidy in cell cultures that may allow transposon insertion numbers to proliferate in less predictable manners (2,33). In contrast, lab and wild fly strains have more defined limits of new transposon insertions, with each strain carrying several hundred new TE insertions. Although additional insertions can be detected with greater sequencing depth, these new insertions tend to have low (<1.0) CRs, indicating that these TE copies are rarer amongst all the genomes sampled in each library. Common lab strains, and even sub-strains with the same lab strain name, also differ in their TLs, which in some cases is almost certainly due to contamination or introgression (i.e. the introduction of the *P-element* in some OreR strains, Figure 3C). Finally, pooled fly samples typically reveal many more TE insertions relative to individual lab or wild strains, with additional sequencing coverage being able to identify more insertions in pooled samples.

The CRs that TIDAL determines can inform which TE insertions can be detected by genomic PCR, because it reflects the ‘penetrance’ of each transposon InDel within each genomic library (Figure 1B and C). However, the striking difference of CR distributions between fly strains and pools of flies also suggest a need to reexamine how TLs should be interpreted if they are derived from pools of flies versus collecting various individual strains. In fly strains, TE InDels with very high CRs (>4) may likely represent germline transposition events in past generations that are present in most cells in the organism. However, the meaning of the bulk of insertions with CR values <4.0 (Figure 5E) is less clear, and some proportion of these events may reflect somatic insertions. Future sequencing experiments with individual flies and comparisons of the soma to germline is needed to test if somatic transposition events are perhaps more prevalent than appreciated. Despite the economy of sequencing pools of flies compared to the more demanding collection and sequencing of many individual fly strains (9), we suggest caution in evaluating TL differences between pools of flies without considering CRs because differences in allele frequencies may confound interpretation of TE location or abundance. For example, each of the many TE insertions in pooled fly samples that have low CRs might be germline events within a few individual flies that are then diluted by the pervasive diversity of unique TLs from other flies within the pool. Alternatively, if somatic transposition is common, the interpretation of low CRs insertions from pooled samples would still be challenging since somatic insertions could be conflated with low frequency alleles.

Recently, genomic mosaicism was observed between *D. melanogaster* follicle cells and salivary glands (62), and transposons have been described as sources of genome variation amongst individual neurons (76,77). With TIDAL, we are better poised to further explore these and other phenomena in *D. melanogaster*. For example, an emerging hypothesis for animal aging proposes the progressive incapacity of older cells to reign in transposons (78), and several lines of evidence in *D. melanogaster* suggest aging animals display signatures of heterochromatin mark alterations that would be molecularly indicative of TL expansion (77,79). Furthermore, some transposon insertions have been reported to show signatures of adaptation (31,32,80), and TIDAL could enable deeper scrutiny of individual strains possessing the most penetrant TE insertions near genes in these fly strains.

TIDAL has the limitation of not being able to analyze new transposon InDels in TE-dense heterochromatic regions, including large intergenic piRNA clusters that serve as genetic traps for new TE insertions that then prevent future transposon mobilization events (81–83). This limitation is tied to the short length of Illumina reads that cannot be mapped uniquely in repetitive regions nor span the lengths of full transposons. Extremely-long read (>20 kb) sequencing technologies like the PacBio system may pave the way for future routine sequencing efforts of *D. melanogaster* genomes when its costs can approach the Illumina platform. For example, the ISO1 strain's genome was recently re-sequenced and completely re-assembled *de novo* from PacBio runs, with impressive closure of previous genome gaps that included transposons (84,85). Our data suggest that frequent transposon depletions overlapping between disparate *D. melanogaster* genomes may actually reflect specific TE insertions to the ISO1 sub-strain used for the reference genome. We therefore propose that the field could benefit from PacBio sequencing of new *D. melanogaster* reference genomes such as population-specific type strains or commonly-used cell lines to provide additional reference states that would improve our understanding of the transposon architecture in these genomes.

Our bioinformatics results also allow us to compare genome-wide data against the previous classic studies of transposon accumulation and transposition rates in *D. melanogaster*. Related lab stocks have been previously shown to display distinct restriction length polymorphisms from transposon probes (86), while other lab lines undergoing mutation accumulation have been examined with low-resolution approaches like in situ hybridization of individual TE families in polytene chromosomes to estimate transposition rates (64,73,74,87–97). These studies described transposition rates ranging from ∼10^−4^ to 10^−5^ per TE copy per generation for several TE families. Making an assumption of ∼15 years separating the ISO1-BL strain from the original ISO1 strain whose DNA comprises the reference genome sequence (18), and using a 12-day average *Drosophila* generation time, our TIDAL outputs derived transposition rates for active families that are on the same order of magnitude as previous studies (Supplementary Figure S5H). Finally, Nuzhdin et al. predicted that inbreeding flies would foster increases in transposition (87,98). TIDAL outputs also support this prediction by revealing higher average numbers of TE insertions in lab strains inbred for decades (∼790), versus DGRP fly strains which have only recently been inbred (∼670), and these are also higher than outbred DGN fly strains (∼550).

Understanding rates of transposition in other genetic and environmental contexts remains an open question, and this can now be studied accurately using high-resolution genome sequencing and TIDAL. For example, it is now appreciated that the Piwi/piRNA pathway acting in the animal germline may strongly modulate the evolution of TLs (88,99–101), to a point where TL diversity might be an expected outcome of the Piwi/piRNA pathway by preventing detrimental explosions of transposition, but allowing some low level of transposition to occur. If wild *D. melanogaster* are constantly allowing low levels of transposition activity (102), this activity can be intensified by various natural mechanisms such as horizontal transfer (103–106), stressful environmental conditions (107,108), permissive alleles in the host (109), or crosses between distant strains (110). In addition, our comparisons of TE insertions versus piRNA content in DGRP strains suggest that *roo* may be an example of a transposon that has been able to evade Piwi/piRNA silencing by unknown mechanisms (Figure 6B). Our future interests will be to re-examine the rates of transpositions by genome sequencing and TIDAL analysis of direct lineages of flies subjected to stress and aging, and in sub-fertile mutants of the Piwi pathway. With this high-resolution genomics approach, we will better understand the détente between genomes and the genetic parasites that reside within them.

## NOTE ADDED IN PROOF

In a very recent update of the TEMP program applied to two DGRP libraries (RAL-109 and RAL-229) and the Release 6 genome, there was greater overlap in TE insertion calls between TEMP and LnB and TIDAL. However, many program-specific calls remain, requiring future continuing development of all TE discovery programs.

## Supplementary Material

SUPPLEMENTARY DATA
