# Supplementary material for: Unique transposon landscapes are pervasive across Drosophila melanogaster genomes
Source: Nucleic Acids Res. 2015 Nov 17;43(22):10655–72. doi: 10.1093/nar/gkv1193 (PMC4678822; doi:10.1093/nar/gkv1193)
Supplement: SUPPLEMENTARY DATA [file supp_43_22_10655__index.html]

Unique transposon landscapes are pervasive across Drosophila melanogaster genomes — SUPPLEMENTARY DATA 

# Unique transposon landscapes are pervasive across *Drosophila melanogaster* genomes

## SUPPLEMENTARY DATA

- SUPPLEMENTARY DATA
