# Supplementary material for: Unique transposon landscapes are pervasive across Drosophila melanogaster genomes
Source: Nucleic Acids Res. 2015 Nov 17;43(22):10655–72. doi: 10.1093/nar/gkv1193 (PMC4678822; doi:10.1093/nar/gkv1193)
Supplement: SUPPLEMENTARY DATA [file supp_gkv1193_nar-02846-h-2015-File003.pdf]

Rahman, et al.

SUPPLEMENTARY MATERIALS

SUPPLEMENTAL FIGURES AND TABLES LEGENDS

Figure S1. Detailed flowchart of the TIDAL program.

After the split, transposon insertions are detected through the steps on the left, while depletions are detected through the steps on the right. Complete parameter details are described in the Methods section.

Figure S2. Comparison of the overall transposon distributions and genomic architecture between the current Release6/dm6 build and the previous Release5/dm3 build, and functional annotations of TE InDels in cell line genomes.

(**A**) The percentage content of RepeatMasker masked sequences per 50kb window is shown above the banding patterns for each chromosome of the *D. melanogaster* genome. Many of the regions previously absent in the diagrams for Release5/dm3 were not assembled as contiguous portions of the main chromosome arms. (**B**) Proportions of the functional annotations for TE InDels in the various cell line genomes. This panel relates to Fig. 2.

Figure S3. Genomic PCR validation of TIDAL predictions for *D. melanogaster* S2c1 cells (Part 1) and ISO1 sub-strains (Part 2).

Part 1: (**A**) Transposon insertions validation tests for S2c1 cells predictions. Top, schematic of primers designed to flank the TE insertion by pairing to unique genomic sequence. Middle, gels of 15 sites tested by PCR with genomic DNA from S2c1 cells, and ISO1-BL and RAL-362 flies. Bottom, table listing the loci with TE insertions tested in the PCR assays above. (**B**) Transposons depletions validation tests for S2c1 cells predictions. Top, schematic of primers designed to flank the TE depletion by pairing to unique genomic sequence. Middle, gels of 10 sites tested by PCR with genomic DNA from S2c1 cells, and ISO1-BL and RAL-362 flies. Bottom, table listing the loci with TE depletions tested in the PCR assays above. Asterisks mark judgement calls for validations based on the absence of the reference genome allele for TE Insertions and

presence of a smaller band in the TE Depletions as an indication that the PCR result was supportive of the TIDAL prediction. **Part 2:** (**C**) Transposon insertions validation tests for ISO1-BL predictions. Top, schematic of primers designed to flank the TE insertion by pairing to unique genomic sequence. Middle, gels of 12 sites tested by PCR with genomic DNA from RAL-362, ISO1-UC, and ISO1-BL flies. Bottom, table listing the loci with TE insertions tested in the PCR assays above. (**D**) Transposons depletions validation tests for ISO1-BL predictions. Top, schematic of primers designed to flank the TE depletion by pairing to unique genomic sequence (left) and a PCR that uses one reverse primer base-pairing to the TE sequence present in the reference genome sequence (right). The reverse primer against the TE is more prone to generating a TE amplicon artifact. Middle, gels of 12 sites tested by PCR with genomic DNA from RAL-362, ISO1-UC, and ISO1-BL flies. Left gels are PCRs from primers that both flank the TE, while the right gels are PCRs with one reverse primer against TE sequence, which tends to fail more frequently with primer dimers. Bottom, table listing the loci with TE depletions tested in the PCR assays above.

Figure S4. Additional analyses of TLs in Canton-S sub-strains.
(**A**) Euler diagrams showing the overlap of TE InDels between sub-strains from 5 different labs, with all TE InDels in the top diagrams, whereas below are only the TE InDels with CR≥3.0. (**B**) Similar Euler diagrams as in (**A**) except that the CanS-JC sub-strain replaces the CanS-SH sub-strain. These diagrams are to illustrate the notably stronger overlap in TE insertions between the CanS-JC and the CanS-TP sub-strains, which are two lines maintained independently but both originating from the same single lab source. (**C**) Proportions of all of the InDels by TE family between the CanS sub-strains. (**D**) Proportions of InDels with CR≥3.0 by TE family between the CanS sub-strains, which is generally similar to the proportions of all TE InDels in (**C**). (**E**) Proportions of the genomic functional annotations immediately near the sites of TE InDels. (**F**) Scatterplot comparing sequencing depth to numbers of TE InDels detected amongst CanS sub-strain libraries (various combinations of independent sequencing runs). Trend lines show linear relationship between sequencing depth and server

processing time, and logarithmic relationship between depth and numbers of TE InDels detected by TIDAL.

Figure S5. Additional analyses of TLs in ISO1 sub-strains.

(**A**) Euler diagrams comparing the overlap of TE InDels between –BL and –CL sub-strain of ISO1, (**B**) between –BL and –AS sub-strains, and (**C**) between different tissues of the ISO1–AS fly line. Abbreviations: BL, Bloomington; CL, Charles Langley lab; AS, Alan Spradling lab; HEm12, Haploid Embryo samples 1&2; Ova, Ovaries; LSg123, Larval Salivary gland samples 1,2,3; Emb123, Embryos sample 1,2,3. The ISO1-BL library is from adult females from the Bloomington Drosophila Stock Center, the ISO1-CL libraries (whole flies and haploid embryos) are from the Langley lab, and the ISO1-AS libraries (ovaries, embryos, and larval salivary glands) are from the Spradling lab. (**D**) Proportions of InDels by TE family between ISO1 sub-strains. (**E**) Comparison of the InDels proportions by TE family predicted by TIDAL from different combinations of sequencing runs of the ISO1-BL libraries. Greater sequencing depth generally uncovers the rarer TE InDels in the genomic library. (**F**) Scatterplot comparing sequencing depth to numbers of TE InDels detected amongst ISO1 sub-strains. Trend lines show linear relationship between sequencing depth and server processing time, and logarithmic relationship between depth and numbers of TE InDels detected by TIDAL. (**G**) Scatterplot comparing sequencing depth to numbers of TE InDels detected amongst various combinations of independent sequencing runs of the ISO1-BL library. (**H**) Calculations of transposition rates in the ISO1-BL sub-strain for each TE per genomic copy and per generation, assuming that ~450 generations (~15 years between the sequencing of ISO1-BL and the original reference genome isolate, and an average 12 day life cycle).

Figure S6. Additional benchmarking results comparing TIDAL with LnB, CnT, and TEMP programs for TE insertion predictions in DGRP fly strains.

(**A**) For DGRP strains that are only analyzed by TEMP and TIDAL, there is much greater overlap in TE depletion predictions between the two programs (red bars) compared to the TE insertion predictions. (**B**) Table comparing the general design,

distinctions, and limitations of our TIDAL program compared to the other three programs with common outputs for DGRP strains. A diagram of chromosome 4's high density of transposons, from Figure S2, is shown as a reminder for how false positives can be spotted in other programs. (**C**) Euler diagrams of the overlap in TE insertions predicted by all four programs for RAL-109 and RAL-391 strains, with diagrams on the left showing all TE insertions and the diagrams on the right that lack obvious false positives such as Chr4 and Chr#Het predictions, these are frequently repetitive genomic loci that for some reason have not been masked by RepeatMasker. Note the removal of these false positives largely only reduce the predictions unique to TEMP and CnT programs.

Figure S7. Comparison of genomic PCR validation tests between TIDAL, LnB, CnT and TEMP programs for transposon insertions predictions for the RAL-765 strains. (**A**) Transposon insertions only predicted by TIDAL. Top, gels of 12 sites tested by PCR with genomic DNA from RAL-765, RAL-362, RAL-517, and ISO1-BL flies. Bottom, table listing the loci with TE insertions tested in the PCR assays above. The same format of panel A extends to panels B, C, and D. (**B**) Transposon insertions predicted by LnB which also mostly overlap with TIDAL calls. (**C**) Transposon insertions only predicted by the TEMP program. (**D**) Transposon insertions only predicted by the CnT program. Asterisks mark judgement calls for validations based on the absence of the reference genome allele for TE Insertions and presence of a smaller band in the TE Depletions as an indication that the PCR result was supportive of the TIDAL prediction.

Figure S8. Detailed profiles of transposon depletion landscapes in DGRP and DGN fly strains and pools of flies. (**A**) 45 DGRP Fly strains. (**B**) 46 DGN Fly strains. (**C**) 32 Pools of flies. The top panels show the total counts of transposon insertions, the middle panels show the proportions of each transposon class, and the bottom panels show the proportions of the transposon species with ≥20 insertions (the rest are grouped together at the top). The dashed lines and labels in the bottom panels mark notable transposon species.

Figure S9. Detailed profiles of transposon insertion landscapes in DGRP and DGN fly strains and pools of flies, referring to the abridged profiles in Fig. 5. (**A**) 45 DGRP Fly strains. (**B**) 46 DGN Fly strains. (**C**) 32 Pools of flies. The top panels show the total counts of transposon insertions, the middle panels show the proportions of each transposon class, and the bottom panels show the proportions of the transposon species with ≥20 insertions (the rest are grouped together at the top). The dashed lines and labels in the bottom panels mark notable transposon species.

Table S1. Details and accession numbers for Illumina libraries of *D. melanogaster* cell lines and fly strains analyzed in this study and incorporated into the TIDAL-Fly database. There are 5 tabs in this worksheet. (**A**) *D. melanogaster* cell lines. (**B**) DGRP fly strains. (**C**) DGN fly strains. (**D**) Key for the demographics of DGN fly strains. (**E**) Pools of flies.

Table S2. Oligonucleotides used in this study.

Rahman, et al.
Figure S1.

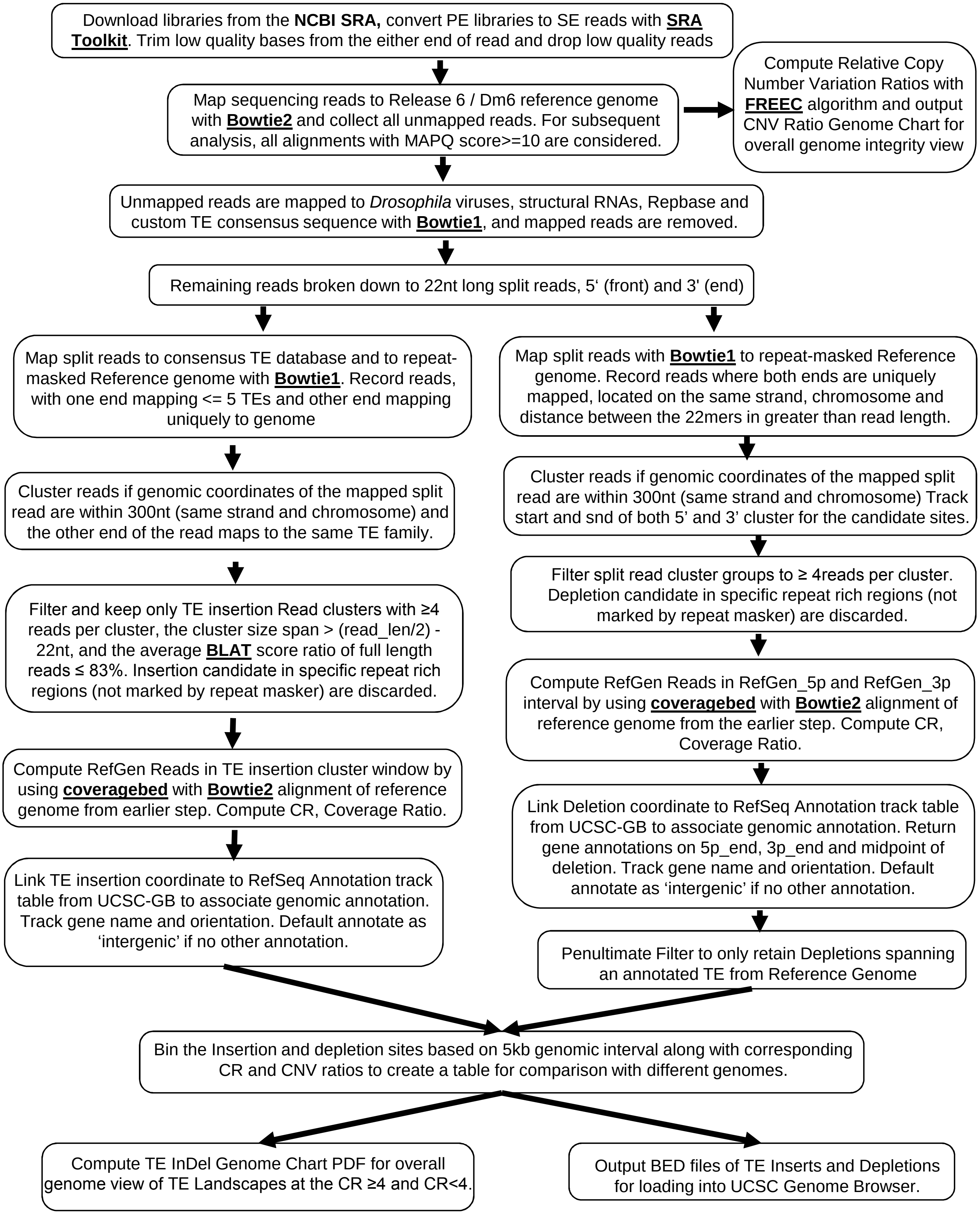

See Methods Text for details of program parameters and equations.

Rahman et al.
Figure S2.

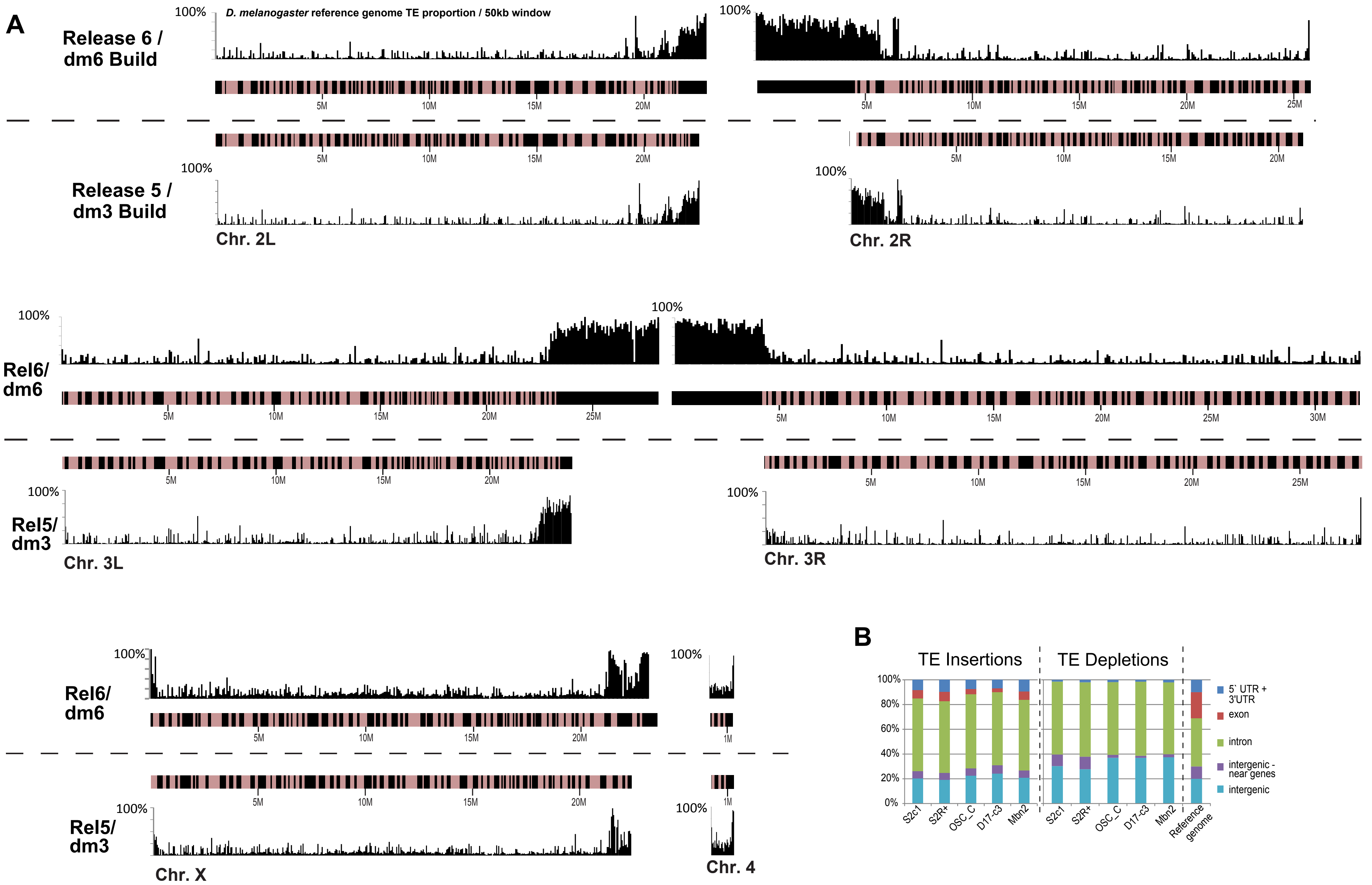

Rahman, et al. Figure S3, PART 1

A

TE insertions genomic PCR

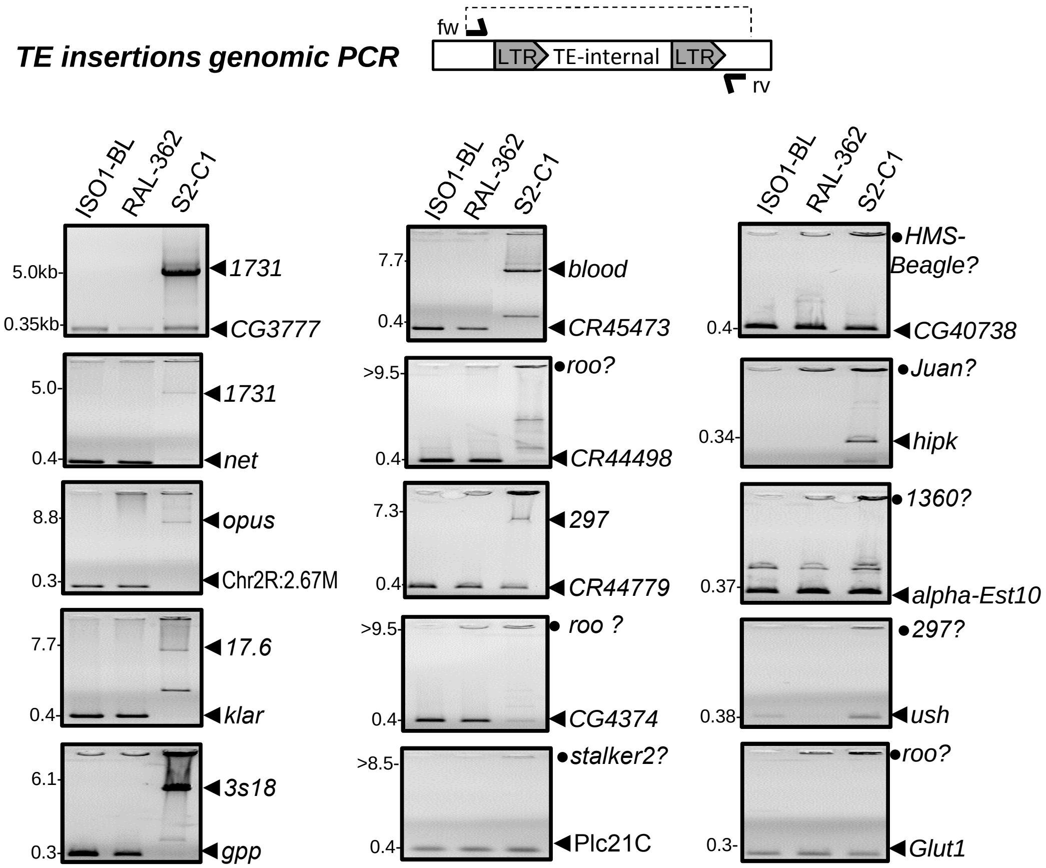

Site. No.	T.E.	Locus	Nearest Gene	Coverage Ratio	Size w/o insertion	Size with insertion	PCR evidence
1	*1731*	chrX:325934-326139	CG3777 +,CG32816 -	4.5	349	4998	yes
2	*1731*	chr2L:87055-87258	net -	4	404	5053	yes
3	*opus*	chr2R:2675584-2675743	not near genes	3.4	332	8891	yes
4	*17.6*	chr3L:516184-516383	klar -	5.1	349	7788	yes
5	*3S18*	chr3R:6430955-6431163	gpp +	5.6	293	6126	yes
6	*blood*	chrX:141518-141720	CR45473 -	5.6	379	7790	yes
7	*roo*	chrX:6005755-6005959	CR44498 +	5	381	9473	yes*
8	*297*	chrX:1082310-1082488	CR44779 +,CG3655 -	3.7	373	7368	yes
9	*roo*	chr3R:23470563-23470775	CG4374 -	4	367	9459	yes*
10	*Stalker2*	chr2L:312233-312444	Plc21C +	5.9	411	8661	no
11	*HMS-Beagle*	chr2R:544728-544940	CG40378 +	4.9	353	6882	yes*
12	*Juan*	chr3L:552782-552981	hipk +	3.3	344	4580	no
13	*1360*	chr3R:7492347-7492543	alpha-Est10 -	4.7	374	4480	no
14	*297*	chr2L:522270-522480	ush +	4.9	386	7381	no
15	*roo*	chr3L:957937-958157	Glut1 +	7.6	296	9388	no

B

TE depletions genomic PCR

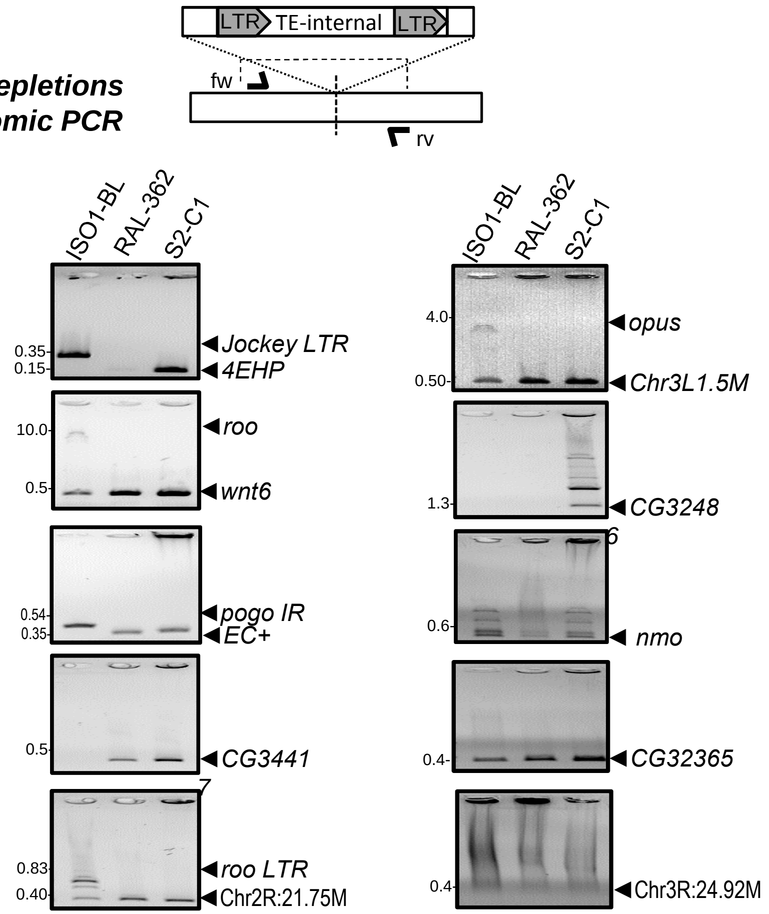

Site. no.	T.E.	Locus	Nearest Gene	Coverage Ratio	Size with depletion	Size without depletion	PCR evidence
1	jockey	chr3R:24072059-24072628	4EHP -	4.5	150	341	yes
2	roo	chr2L:7343256-7350536	Wnt6 +	4.6	5757	7480	yes
3	pogo	chrX:3842276-3842647	ec +	5.8	355	541	yes
4	Tirant	chrX:6562758-6571399	CG34417 +	5.2	1240	8802	yes
5	roo	chr2R:21753020-21753663	not near genes	5.2	405	833	yes
6	opus	chr3L:15423693-15426700	not near genes	4.6	2642	3160	yes
7	Tirant	chr3L:3061219-3069958	CG32486 -	4.6	1350	8912	yes*
8	Stalker4	chr3L:7886449-7889129	CG32365 +	4.3	1107	2873	no
9	NOF	chr3L:7985303-7987783	nmo +	4.2	620	2650	no
10	Doc	chr3R:24927069-24932010	not near genes	5.7	395	5120	no

Rahman, et al. Figure S3, PART 2

C ***TE insertions genomic PCR***

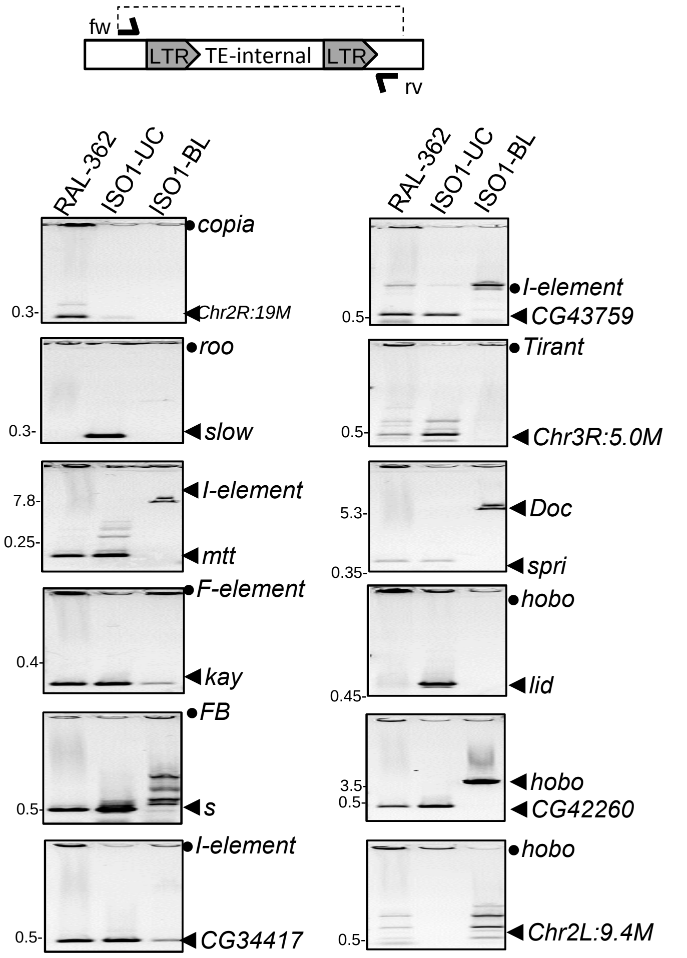

D ***TE depletions genomic PCR***

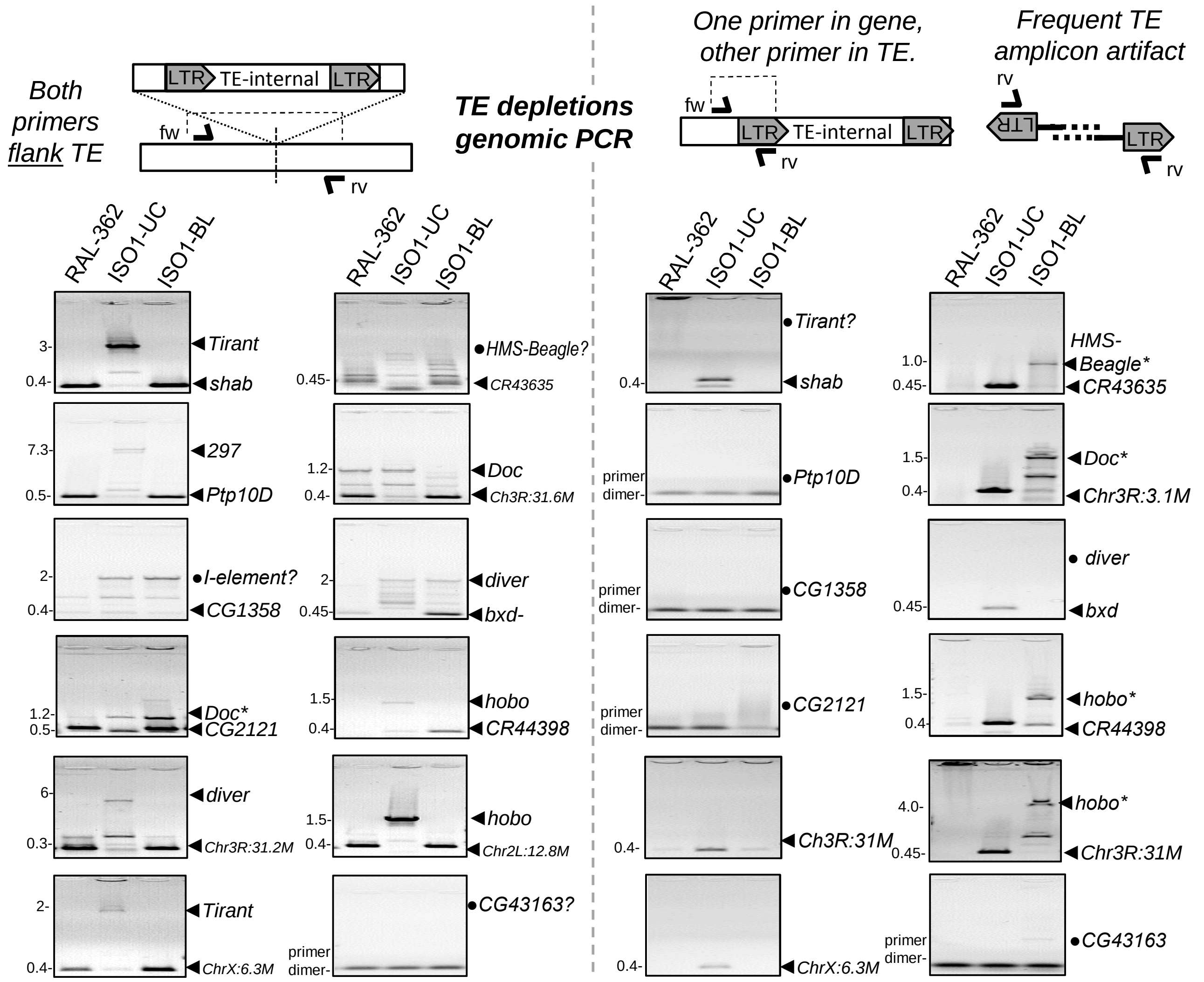

Site No.	Locus	Nearest gene	T.E.	C.R.	size w/o insertion	size with insertion	PCR validation
1	chr2R:19768184-19768307	not near genes	copia	4.5	295bp	5.1kb	yes
2	chr3L:4390337-4390468	slow	roo	4.5	330bp	9kb	yes
3	chr2R:8528862-8528983	mtt	I-element	4.4	273bp	7.8kb	yes
4	chr3R:29777197-29777247	kay	F-element	17.9	414bp	5kb	yes
5	chr2L:1068653-1068778	s	FB	4.2	517bp	2kb	yes
6	chrX:6542904-6543018	CG34417	I-element	4.3	501bp	7.9 kb	yes
7	chrX:19018636-19018748	CG43759	copia	4.7	469bp	5.6kb	yes
8	chr3R:5066592-5066727	not near genes	Tirant	4.4	515bp	9kb	yes
9	chrX:10556247-10556301	spri	Doc	5.7	363bp	5.3kb	yes
10	chr2L:5999659-5999785	lid	hobo	5.6	479bp	3.5kb	yes
11	chr2R:22786248-22786444	CG42260	hobo	5.3	523bp	3.5 kb	yes
12	chr2L:9485261-9485390	not near genes	hobo	4.2	514bp	3.5kb	no

Site No.	Locus	Nearest gene	T.E.	C.R.	size with depletion	size without depletion	PCR validation	
							Flank TE	Gene-T.E.
1	chr3L:2927813-2927871	shab+	Tirant	3.5	393bp	8.5kb	Yes	yes
2	chrX:11633392-11633445	Ptp10D+	297	2.9	497bp	7.3kb	Yes	No
3	chr2R:7608382-7608431	CG1358-	I-element	2.6	509bp	5.4kb	No	No
4	chr2R:8398189-8398240	CG2121-	Doc	3	502bp	5.0kb	Yes*	No
5	chr3R:31279353-31279405	not near genes	diver	2.5	436bp	6.4kb	Yes	yes
6	chrX:6335185-6335241	not near genes	Tirant	4.6	380bp	2.7kb	Yes	yes
7	chr3R:5045818-5045876	CR43635-	HMS-Beagle	4.4	448bp	7.3kb	No	yes*
8	chr3R:31691509-31691562	not near genes	Doc	4.3	449bp	5.0kb	Yes	yes*
9	chr3R:16756294-16756346	bxd-	diver	3.6	436 bp	6.4kb	Yes	yes*
10	chr2L:17951924-17951978	CR44398-	hobo	3.5	381bp	1.7kb	Yes	yes*
11	chr2L:12861671-12861728	not near genes	hobo	3.5	458bp	1.7kb	Yes	yes*
12	chr3L:8481746-8481802	CG43163-	roo	3.3	487bp	9.4kb	No	No

Rahman et al.
Figure S4.

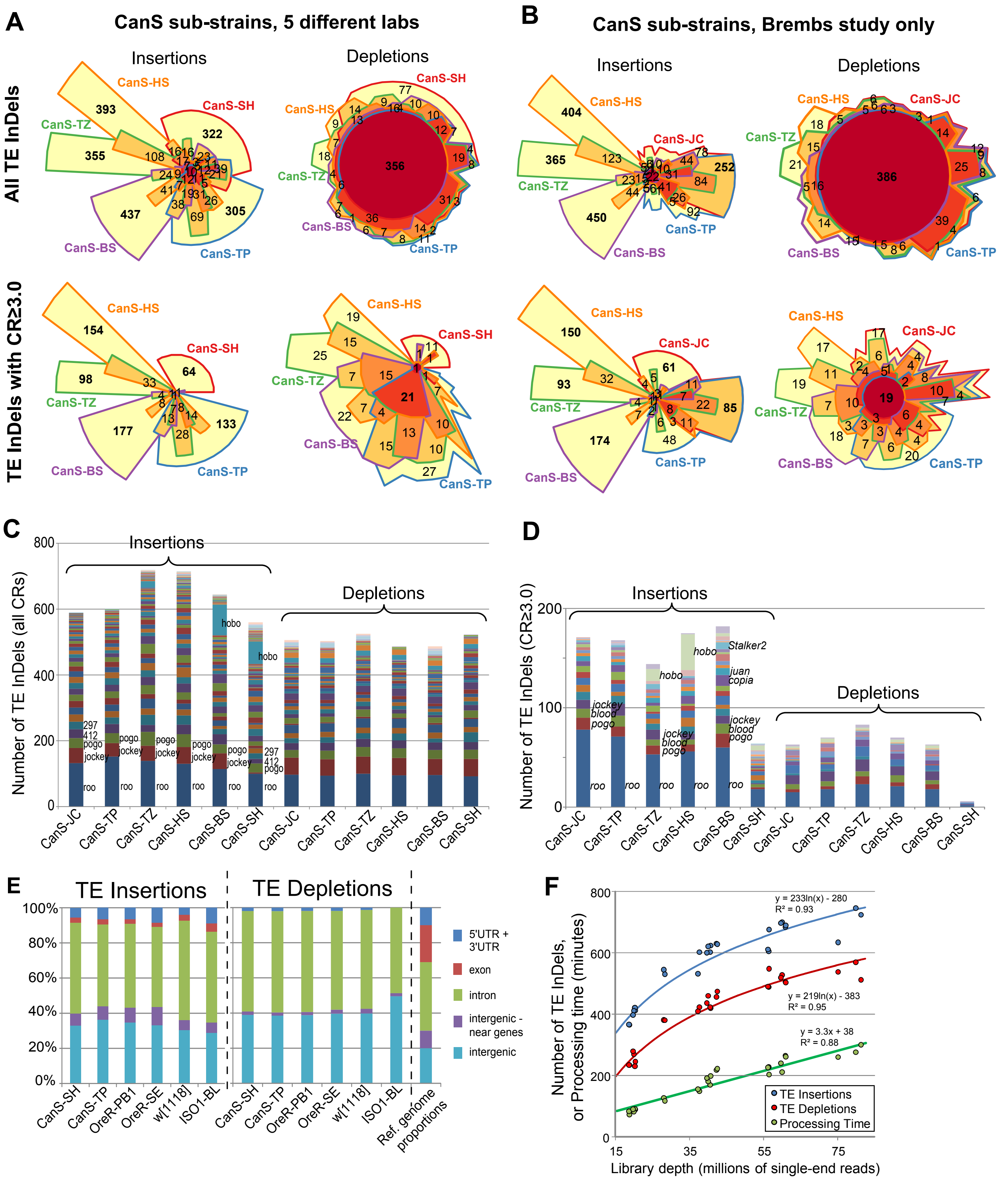

Rahman et al.
Figure S5.

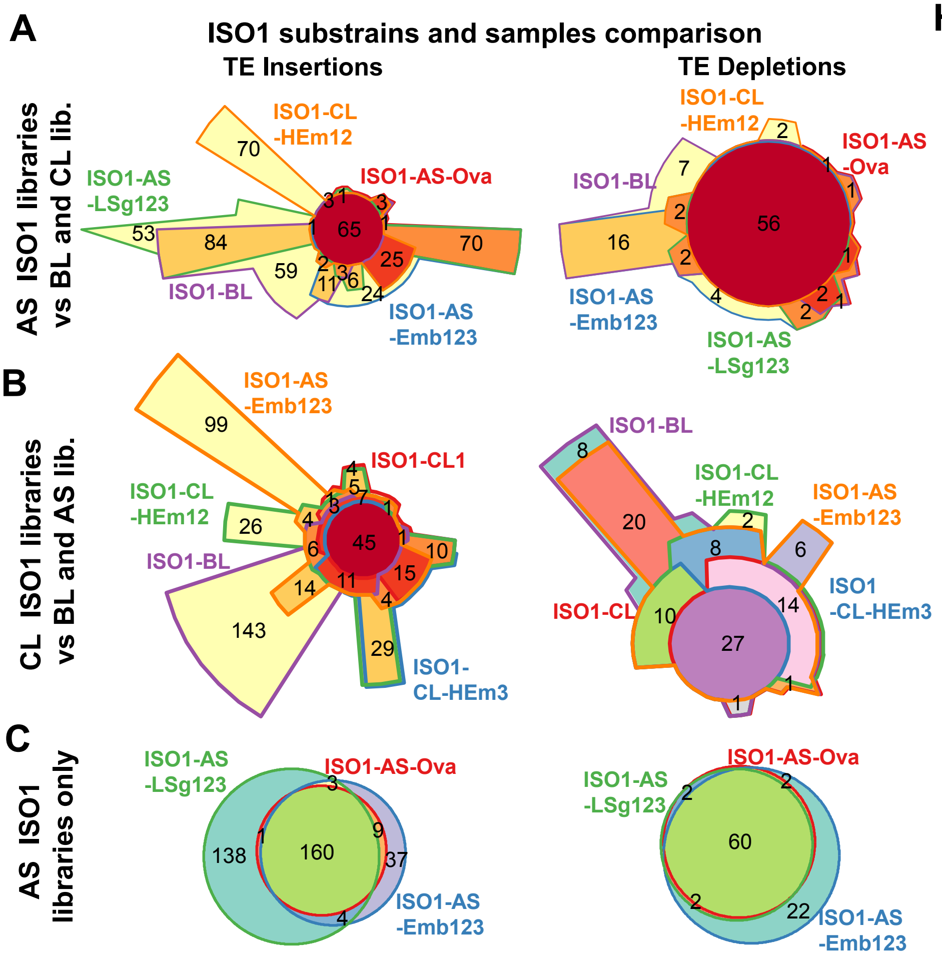

H

New TE insertions in current ISO1-BL genome	# New insertion	# Existing insertions	Rate/copy/generation
hobo	43	201	4.8E-04
FB	29	329	2.0E-04
Doc	27	276	2.2E-04
F-element	27	471	1.3E-04
roo	24	1000	5.3E-05
copia	21	302	1.5E-04
Tirant	13	127	2.3E-04
I-element	13	235	1.2E-04
HMS-Beagle	6	350	3.8E-05
diver	4	53	1.7E-04
1360	3	836	8.0E-06
Stalker4	2	408	1.1E-05
jockey	2	178	2.5E-05
297	2	451	9.9E-06
accord	2	46	9.7E-05
Quasimodo	2	421	1.1E-05
Idefix	2	623	7.1E-06
1731	1	121	1.8E-05
Chimpo	1	62	3.6E-05
Tc1-2	1	75	3.0E-05
Cr1a	1	1548	1.4E-06
diver2	1	452	4.9E-06
Stalker	1	408	5.4E-06
Max-element	1	342	6.5E-06
rover	1	184	1.2E-05
G3	1	103	2.2E-05
mdg1	1	316	7.0E-06
McClintock	1	65	3.4E-05
invader2	1	209	1.1E-05
invader6	1	112	2.0E-05
Doc3-element	1	325	6.8E-06
Average of each individual TE rate:			7.0E-05

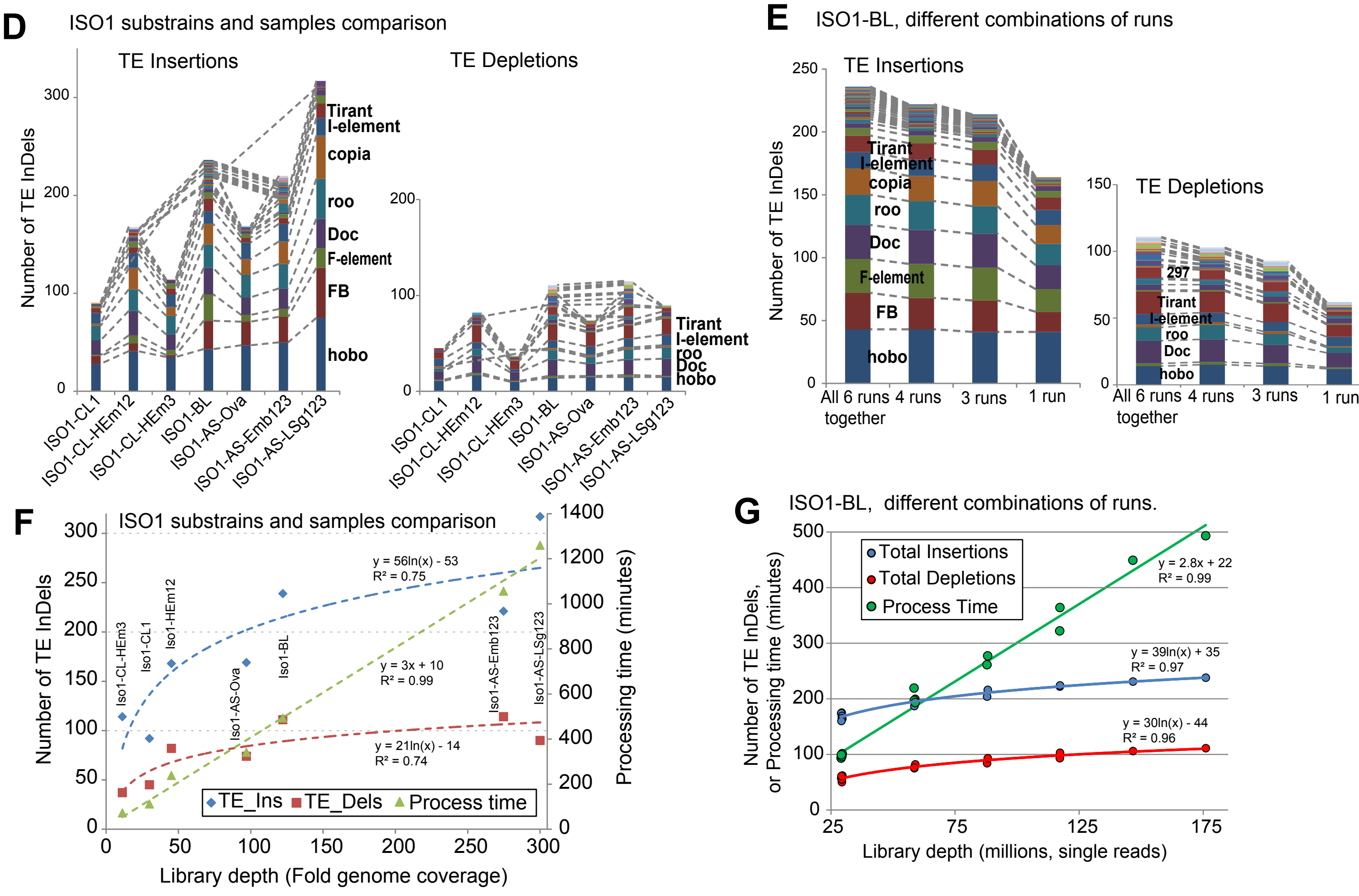

Rahman et al.
Figure S6.

A

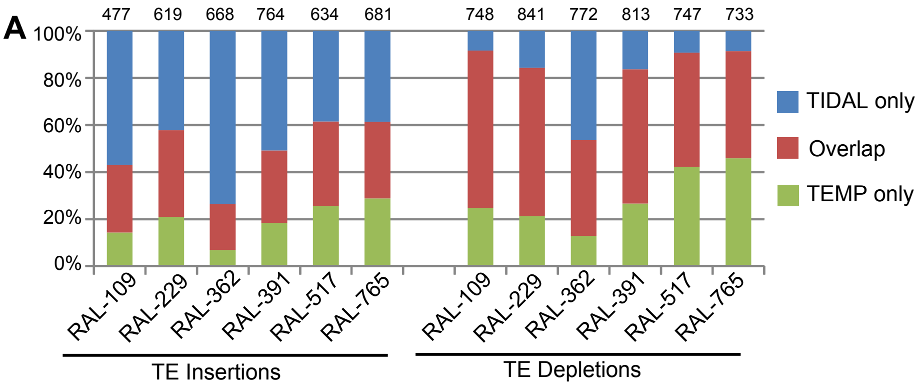

B

Program	TIDAL-Fly	TEMP	CnT	LnB
Citation	(Rahman et al, this study)	(Zhuang et al, 2014)	(Cridland et al, 2013)	(Linheiro and Bergman, 2012)
Pipeline summary	Bowtie1 aligner for split reads, Bowtie2 aligner for reference genome reads, and BLAT to enforce TE insertion read calls. Only single-end split reads processed for discordant mappings on both sides of InDel.	BWA aligner, full tracking of matched paired ends, comparison to reference supporting reads across strains and computes a population frequency.	BWA aligner, full tracking of matched paired ends, reconstruction of candidate InDels, use of BlastN for validation	BLAT aligner for split reads, first stage identifies discordant reads, second stage selects only unambiguous reads on both sides of TE insertion.
Distinctions	Optimization of split short-read approaches to balance sensitivity with specificity. Strain-specific TE landscapes in user-accessible outputs that include rich genomic annotations for Dm6/Rel6 genome release.	Versatility in application being applied to both *Drosophila* and human genomes. Strain-specific DGRP InDels genomic locations can be extracted.	Outputs and analyses focused on population genetics studies for *Drosophila*.	Identifies specific Target Site Duplication sequences. First split-read pipeline applied to both 454-Roche and Illumina datasets from DGRP fly genomes.
Limitations	Excludes any TE InDels in major heterochromatin, given high mapping ambiguity. Cannot automatically determine Target Site Duplication sequences. Currently only optimized for *Drosophila melanogaster* genomes.	Several program-specific predictions in *Drosophila*, some in heterochromatic regions, that have potential to be artifacts. Libraries with multiple read lengths can trip up program.	Many program-specific predictions, some in heterochromatic regions, that have potential to be artifacts. Outputs only available for libraries meeting read depth cutoff. TE Depletion data at broad population level, not strain level.	Pioneering pipeline sacrificed sensitivity by promoting strong specificity of BLAT algorithm. Only detects TE insertions, no capacity to detect TE depletions.

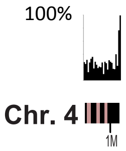

C

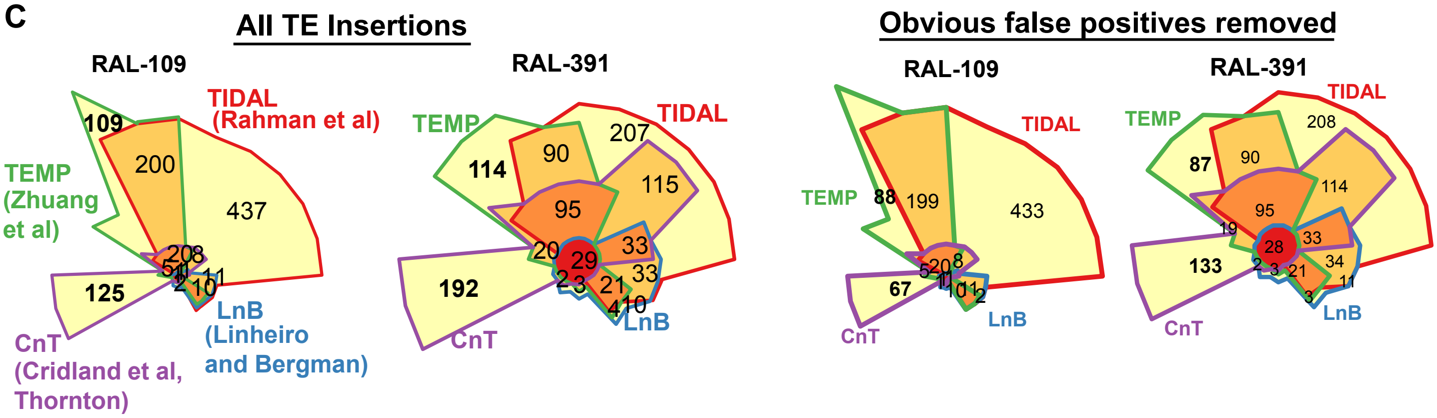

Rahman, et al., Figure S7, Part 1

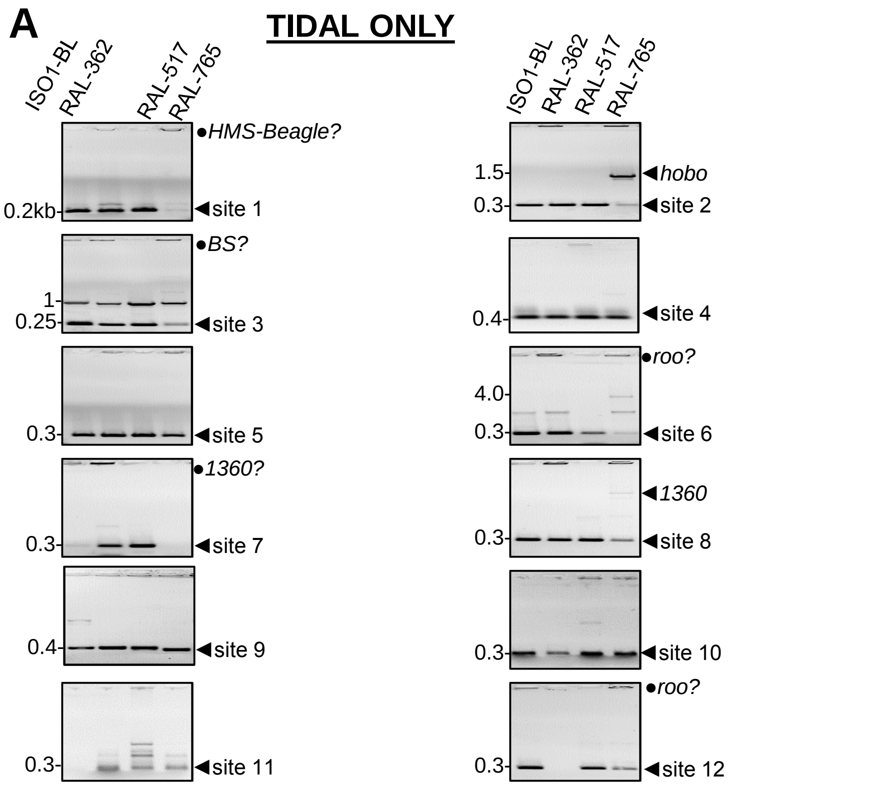

Site. No.	T.E.	Locus	Coverage Ratio	Size w/o insertion	Size with insertion	PCR evidence
1	HMS-Beagle	chr2L8133670::-8133752	2.4	275	7575	yes
2	hobo	chr2L:19834994-19835061	4.7	354	3354	yes
3	BS	chr2L:12227998-12228146	5.3	274	5374	yes
4	pogo	chr2R:12581030-12581105	5	333	2533	no
5	mdg1	chr2R:7621371-7621430	4	328	7628	no
6	roo	chr3L:4481564-4481640	4.1	337	9337	yes
7	1360	chr3L:18394377-18394445	4.2	360	4760	yes
8	1360	chr3R:4885334-4885399	1.7	354	4754	yes
9	jockey	chr3R:20125023-20125087	2.4	360	5360	no
10	roo	chr3R:11089486-11089562	5.4	359	9359	no
11	hobo	chrX:7785488-7785556	3.3	342	3342	no
12	roo	chrX:15391856-15391929	4.1	341	9341	yes

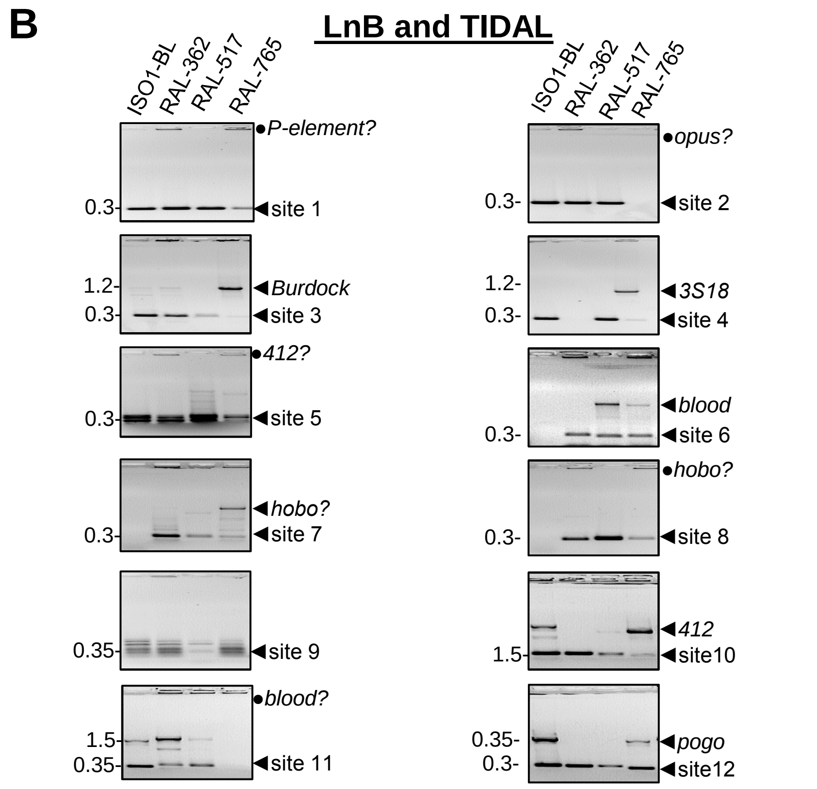

Site. No.	T.E.	Locus	Coverage Ratio	Size w/o insertion	Size with insertion	PCR evidence
1	P-element	chr2L:267478-267547	4.4	314	3214	yes
2	opus	chr2L:1167146-1167232	2.6	386	8386	yes
3	Burdock	chr2L:20800099-20800248	5.8	329	6729	yes
4	3S18	chr2R:9380884-9380961	2.4	365	6465	yes
5	412	chr2R:9442941-9443015	3.9	331	7931	yes
6	blood	chr2R:11578561-11578638	5.6	372	5672	yes
7	hobo	chr3L:934796-934851	8.3	364	3364	yes
8	hobo	chr3L:22566716-22566793	4.3	367	3367	yes
9	Burdock	chr3R:18968019-18968094	3.8	375	6775	no
10	412	chr3R:30626323-30626403	5.2	379	7979	yes
11	blood	chrX:6487781-6487856	4.3	375	5675	yes
12	pogo	chrX:21399401-21399469	3.6	381	2581	yes

Rahman, et al., Figure S7, Part 2

C

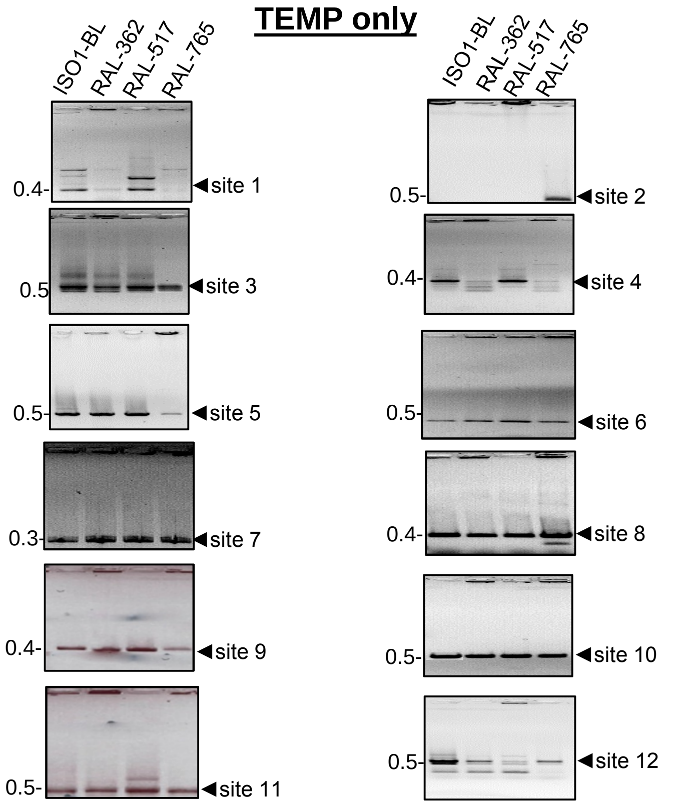

Site. No.	Locus	Amplicon Size	PCR evidence
1	chr2L:9850436-9850443	383	yes
2	chr2L:1558495-1558995	482	no
3	chr2R:5334166-5334666	494	yes
4	chr2R:2095477-2095485	405	no
5	chr3L:9571935-9572435	482	yes
6	chr3L:5056174-5056674	499	no
7	chr3R:5035704-5035739	296	no
8	chr3R:1211275-1211285	398	no
9	chrX:294866-294869	395	yes
10	chrX:5410656-5411156	488	no
11	chrY:203747-204247	487	no
12	chrY:1565254-1565753	645	no

D

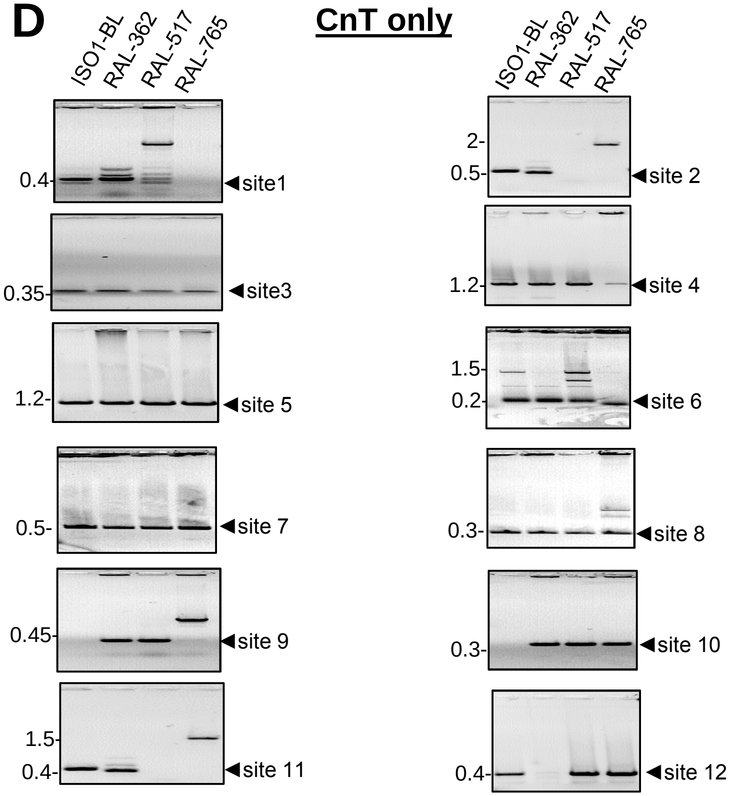

Site. No.	Locus	Amplicon Size*	PCR evidence
1	chrX:4334105-4334327	376	yes
2	chrX:14001033-14007159	6092	yes
3	chr3R:7696150-7703338	7171	no
4	chr3R:14021702-14023463	1734	yes
5	chr3L:4361048-4361901	844	no
6	chr3L:17180645-17180660	394	no
7	chr2R:6948454-6948972	493	no
8	chr2R:13064762-13064861	357	yes
9	chr2L:1809645-1810122	447	yes
10	chr2L:9783398-9783415	394	no
11	chr4:180927-183823	2892	yes
12	chr4:999517-1001425	2008	no

Rahman et al.
Figure S8.

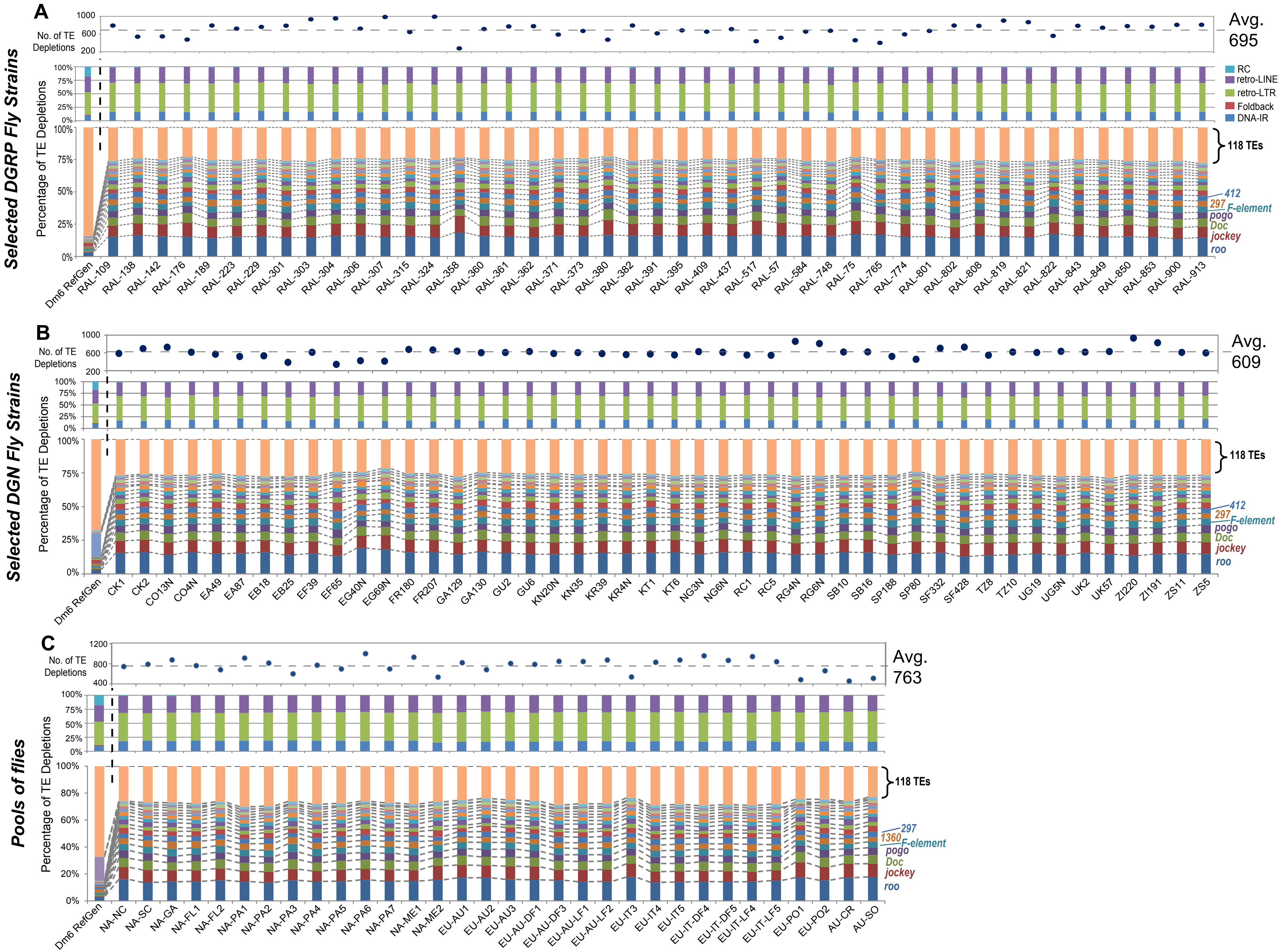

Rahman et al.
Figure S9.

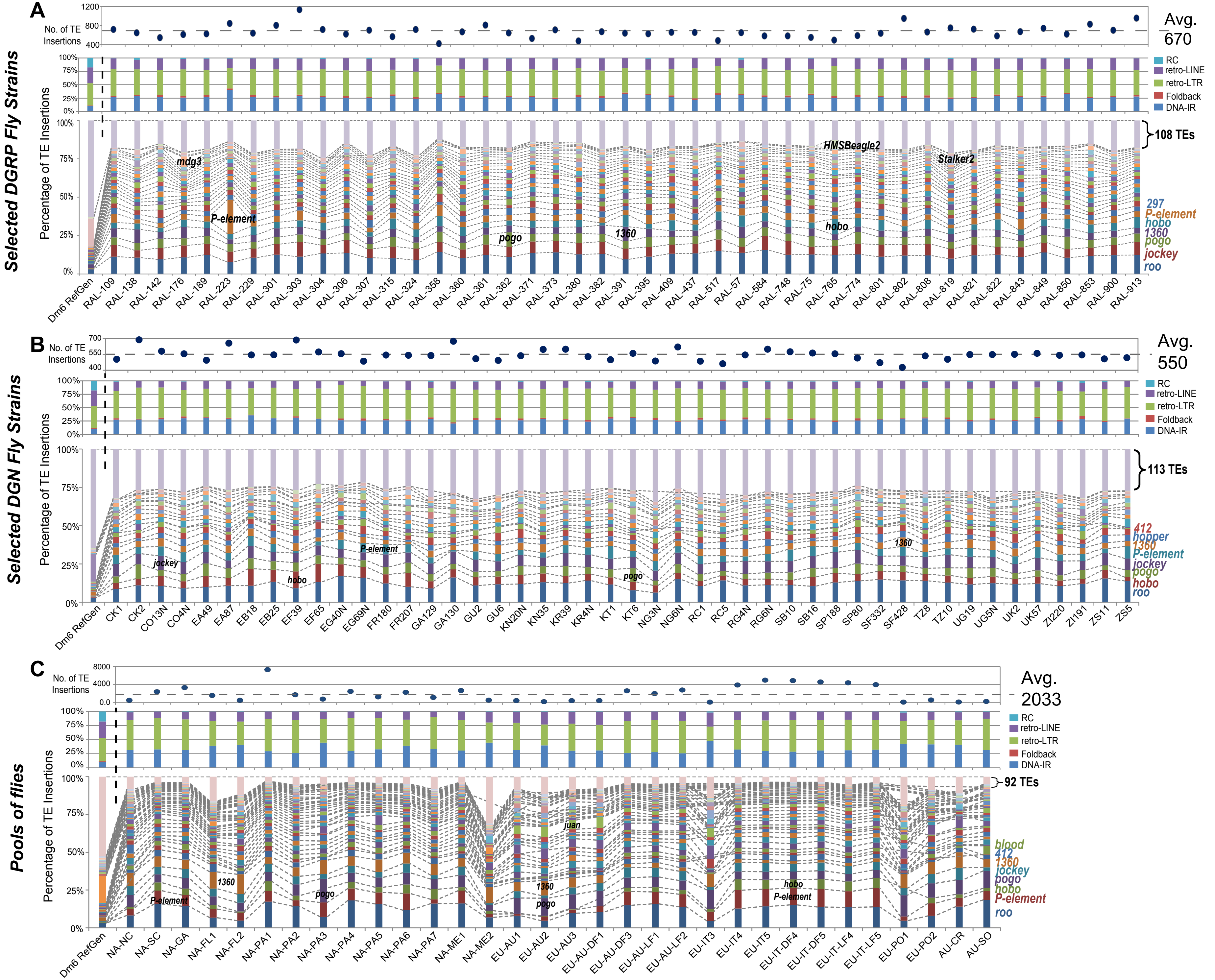

Table S1A. Drosophila Cell Lines in the TIDAL-Fly v1 database

Number	Official name	Tissue origin	Origin genotype	modENCODE Name	Library ID*	SRR Accesion	Min Library Length	Library Size (M)	Read 1 length	Read 2 length	Latest Ref		Other accessions	
1	S2_C1	Embryo	Oregon R	n/a	S2c1	SRR1983913	150	33.8	150	na	This Study			
2	S2R+	Embryo	Oregon R	modENCODE_5492	S2R+	SRR497719	100	7.7	100	100	Lee et al. Genome Bio 2014		SRR497722	2x75
3	S1	Embryo	Oregon R	modENCODE_5491	S1	SRR497713	75	31.8	75	75	Lee et al. Genome Bio 2014			
4	S3	Embryo	Oregon R	modENCODE_5493	S3	SRR497721	100	11.4	100	100	Lee et al. Genome Bio 2014			
5	Sg4	Embryo	Oregon R	modENCODE_5494	Sg4	SRR497720	100	20.5	100	100	Lee et al. Genome Bio 2014			
6	W2	Wing disc	Oregon R	modENCODE_5495	W2	SRR497730	75	30.5	75	75	Lee et al. Genome Bio 2014			
7	CME W1 Cl.8+	L3 wing disc	Oregon R	modENCODE_5480	Cl.8	SRR497726	75	18.8	75	75	Lee et al. Genome Bio 2014			
8	CME L1	L3 leg disc	Oregon R	modENCODE_5489	L1	SRR497712	100	46.9	100	100	Lee et al. Genome Bio 2014			
9	OSS_C	Ovary	?	n/a	OSS_C	SRR1185816	150	33.7	150	na	Sytnikova et al. Genome Res 2014			
10	OSS_E	Ovary	?	n/a	OSS_E	SRR1185793	150	26.6	150	na	Sytnikova et al. Genome Res 2014			
11	OSC_C	Ovary	?	n/a	OSC_C	SRR609664	100	65.7	100	na	Sienski et al. Cell 2013			
12	OSC_E	Ovary	?	n/a	OSC_C	SRR1185771	150	24.6	150	na	Sytnikova et al. Genome Res 2014			
13	ML-DmD16-c3	L3 wing disc	y^1 v^1 f^1 mal^{F1}	modENCODE_5482	D16-c3	SRR497710	100	36.6	100	100	Lee et al. Genome Bio 2014		SRR497715	2x75
14	ML-DmD17-c3	L3 haltere disc	y^1 v^1 f^1 mal^{F1}	modENCODE_5483	D17-c3	SRR497725	100	43.4	100	100	Lee et al. Genome Bio 2014			
15	ML-DmD20-c2	L3 antennal disc	y^1 v^1 f^1 mal^{F1}	modENCODE_5484	D20-c2	SRR497724	75	26.7	75	75	Lee et al. Genome Bio 2014			
16	ML-DmD20-c5	L3 antennal disc	y^1 v^1 f^1 mal^{F1}	modENCODE_5490	D20-c5	SRR497723	100	12.5	100	100	Lee et al. Genome Bio 2014		SRR497718	2x75
17	ML-DmD4-c1	L3 mixed discs	y^1 v^1 f^1 mal^{F1}	modENCODE_5486	D4-c1	SRR497716	75	36.5	75	75	Lee et al. Genome Bio 2014			
18	ML-DmD8	L3 wing disc	y^1 v^1 f^1 mal^{F1}	modENCODE_5487	D8	SRR497729	75	29.4	75	75	Lee et al. Genome Bio 2014			
19	ML-DmD9	L3 wing disc	y^1 v^1 f^1 mal^{F1}	modENCODE_5488	D9	SRR497711	100	30.2	100	100	Lee et al. Genome Bio 2014		SRR497714	2x75
20	1182-4H	Embryo	mh	modENCODE_5479	1182-4H	SRR497717	100	21.1	100	100	Lee et al. Genome Bio 2014			
21	Mbn2	L3 hemocytes	l(2)mbn	modENCODE_5496	Mbn2	SRR497728	75	18.3	75	75	Lee et al. Genome Bio 2014			

Table S1B. Drosophila Lab Strains in the TIDAL-Fly v1 database

Number	Stock ID*	Stock Location	Genome Type	Library ID*	SRA Accession	Min Read Length	PE Reads (M)	Read Length 1	Read Length 2	Data Group	
1	Oregon R	Terry Orr-Weaver	OregonR adult mix male female	OreR-TO1	SRR1538752	125	8.6	125	125	ModEncode	
2	Oregon R	Terry Orr-Weaver	OregonR adult mix male female	OreR-TO2	SRR1538753	85	36.1	85	85	ModEncode	
3	Oregon R	Sarah Elgin	OregonR adult mix male female	OreR-SE	SRR1538754	125	27.7	125	125	ModEncode	
4	Oregon R	Peter Cherbas	OregonR adult mix male female	OreR-PB1	SRR1538751	85	38	125	85	ModEncode	
5	Oregon R	Peter Cherbas	OregonR adult mix male female	OreR-PB2	SRR1538755	125	9.9	125	125	ModEncode	
6	Oregon R	modENCODE_5497_Brent Graveley	OregonR adult virgin female	OreR-BG	SRR497727	75	19.9	75	75	ModEncode	
7	Oregon-R	Dworkin lab	OregonR adult mix male female	OreR-Dw1	SRR1104304, SRR1104305	75	16.7,18	75	75	Dworkin Lab, Genetics paper	
8	DarkFly	Fuse lab	OregonR-S mix	OreR-Dark	DRR001446	48	44.5	48	48	Izutsu Fuse PlosONE paper	
9	Samarkand	Dworkin lab	Samarkand adult mix	Samar-Dw1	SRR1104160, SRR1104161	75	12.9	75	75	Dworkin Lab, Genetics paper	
10	Canton-S substrain JC	Brembs lab, Julien Colomb from Tomas Preat	20 adult females	CanS-JC	ERR744541, ERR744542, ERR744543, ERR744544	100	8.8, 8.7, 8.7, 8.7	100	100	Brembs Lab, F1000 paper and this study	
11	Canton-S substrain BS	Brembs lab, Bruno Swinderen from Ralf Greenspan	20 adult females	CanS-BS	ERR744549, ERR744550, ERR744551, ERR744552	100	7.1, 7.1, 7.2, 7.2	100	100	Brembs Lab, F1000 paper and this study	
12	Canton-S substrain HS	Brembs lab, Henrike Scholz from Ulrike Herberlein	20 adult females	CanS-HS	ERR744545, ERR744546, ERR744547, ERR744548	100	10.3, 10.2, 10.3, 10.2	100	100	Brembs Lab, F1000 paper and this study	
13	Canton-S substrain TP	Brembs lab, Tim Tully lab, Brandeis	20 adult females	CanS-TP	ERR744537, ERR744538, ERR744539, ERR744540	100	9.4, 9.4, 9.5, 9.6	100	100	Brembs Lab, F1000 paper and this study	
14	Canton-S substrain TZ	Brembs lab, Troy Zars from Martin Heisenberg	20 adult females	CanS-TZ	ERR744533, ERR744534, ERR744535, ERR744536	100	10.2, 10.1, 10.0, 10.0	100	100	Brembs Lab, F1000 paper and this study	
15	CanS-SH	Stowers Institute, Scott Hawley lab	Adult mix, iso-2,3,X	CanS-SH	SRR2044310	150	25.5	150	150	Public deposit	
16	w[1118]	Stowers Institute, Scott Hawley lab	Adult mix, iso-2,3,X	w_1118	SRR2044312	150	27.2	150	150	Public deposit	
17	ISO1 - BL	Bergman lab, ISO1 from Bloomington	10 adult males	ISO1-BL	ERR701706, ERR701707, ERR701708, ERR701709, ERR701710, ERR701711	100	15, 14.9, 14.9, 15.1, 14.8, 15.0	100	100	Gutzwiller et al, ArXiv	
18	ISO1-CL1	Langley lab ISO1, mixed adults	mixed adult	ISO1-CL1	SRR350908	75	21.8	100	100	Langley et al (Begun), Genetics 2012	
19	ISO1-CL-HEm3	Langley lab ISO1, haploid embryos	haploid embryos	ISO1-CL-HEm3	SRR306608	100	16.4	100	100	Langley et al (Begun), Genetics 2012	
20	ISO1-CL-HEm12	Langley lab ISO1, haploid embryos	haploid embryos	ISO1-CL-HEm12	SRR097732, SRR306607	75	24.2, 19.7	75	75	Langley et al (Begun), Genetics 2012	
21	ISO1-AS-Ova	Spradling lab ISO1, Ovaries	females	ISO1-AS-Ova	SRR1519054	100	71.5	100	100	Yarosh and Spradline, GenesDev 2014	
22	ISO1-AS-Emb123	Spradling lab, ISO1 embryos	0-18hr embryos	ISO1-AS-Emb123	SRR1516224	100	81.8, 69.1, 50.5	100	100	Yarosh and Spradline, GenesDev 2014	
23	ISO1-AS-LSg123	Spradling lab ISO1, L3 Salivary gland	L3 instar	ISO1-AS-LSg123	SRR1516221, SRR1516223, SRR1516225	100	62.9, 88.4, 68.6	100	100	Yarosh and Spradline, GenesDev 2014	
24	OreR-AS-FC	Spradling lab OreR, stg10-14 follicle cells	females	OreR-AS-FC	SRR1519051	100	43.1	100	100	Yarosh and Spradline, GenesDev 2014	

Table S1C. Drosophila Lab Strains in the TIDAL-Fly v1 database Genomes previously examined in McKay et al, Nature 2012, and Huang et al, Genome Res, 2014

PHASE 1: First Set of Libraries Analyzed for the paper. The remaining libraries in Phase 2 below are also listed in the TIDAL-Fly database

Library	Stock ID*	Stock Location	Genome Type	SRA Accession	Library ID*	SRA Accession	Min Read Length	Read Length 1	Read Length 2	Data Group	Reads (M)	Raw Read Length:Read Number		Freeze		Bloomington Stock	Synonym	NCBI SRA	Reads	Mapped Reads	Mapped Coverage	Strains
1	DGRP_362	Bloomington-25187	inbred line, mix of	SRX006288	RAL-362	SRR846985	125	merged, use min length	125.0	DGRP-F1 + F2	56.3	45bp:18900352, 75bp:19241828, 12	DGRP	F1 + F2	Blo	25187	RAL-362	SRX006288 and	70.4	70,371,466	39.4	1
2	DGRP_517	Bloomington-25197	inbred line, mix of	SRX024363	RAL-517	SRR933579	75	merged, use min length	75.0	DGRP-F1	59.8	45bp:75005702, 75bp:44531158	DGRP	F1	Blo	25197	RAL-517	SRX024363 and	115.8	115,773,424	37	2
3	DGRP_765	Bloomington-25204	inbred line, mix of	SRX006169	RAL-765	SRR933595	75	75.0	75.0	DGRP-F1	19.9	75bp:39893442	DGRP	F1	Blo	25204	RAL-765	SRX006169 and	38.1	38,107,800	16.3	3
4	DGRP_109	Bloomington-28140	inbred line, mix of	SRX020746	RAL-109_75b	SRR835219	75	merged, use min length	125.0	DGRP-F1	44.8	75bp:28899572, 125bp:60695744	DGRP	F1	Blo	28140	RAL-109	SRX020746 and	78.6	78,598,946	48.7	4
5	DGRP_109	Bloomington-28140	inbred line, mix of	SRX020746	RAL-109_125b	SRR835219	125	merged, use min length	125.0	DGRP-F1	44.8	75bp:28899572, 125bp:60695744	DGRP	F1	Blo	28140	RAL-109	SRX020746 and	78.6	78,598,946	48.7	4
6	DGRP_229	Bloomington-29653	inbred line, mix of	SRX021052	RAL-229_75b	SRR835221	75	merged, use min length	125.0	DGRP-F1	49.6	75bp:27785294, 125bp:71504164	DGRP	F1	Blo	29653	RAL-229	SRX021052	83.9	83,937,442	52.7	5
7	DGRP_229	Bloomington-29653	inbred line, mix of	SRX021052	RAL-229_125b	SRR835221	125	merged, use min length	125.0	DGRP-F1	49.6	75bp:27785294, 125bp:71504164	DGRP	F1	Blo	29653	RAL-229	SRX021052	83.9	83,937,442	52.7	5
8	DGRP_301	Bloomington-25175	inbred line, mix of	SRX005978	RAL-301_75b	SRR835223	75	merged, use min length	125.0	DGRP-F2	73.6	45bp:13099368, 64bp:10588312, 75	DGRP	F2	Blo	25175	RAL-301	SRX155995 and	133.5	133,504,636	71.7	6
9	DGRP_301	Bloomington-25175	inbred line, mix of	SRX005978	RAL-301_125b	SRR835223	125	merged, use min length	125.0	DGRP-F2	73.6	45bp:13099368, 64bp:10588312, 75	DGRP	F2	Blo	25175	RAL-301	SRX155995 and	133.5	133,504,636	71.7	6
10	DGRP_303	Bloomington-25176	inbred line, mix of	SRX005986	RAL-303_100b	SRR835228	100	merged, use min length	100.0	DGRP-F2	161.6	45bp:79536584, 64bp:4167822, 10	DGRP	F2	Blo	25176	RAL-303	SRX155978 and	287.4	287,358,990	148	7
11	DGRP_303	Bloomington-25176	inbred line, mix of	SRX005986	RAL-303_125b	SRR835228	125	merged, use min length	100.0	DGRP-F2	161.6	45bp:79536584, 64bp:4167822, 10	DGRP	F2	Blo	25176	RAL-303	SRX155978 and	287.4	287,358,990	148	7
12	DGRP_304	Bloomington-25177	inbred line, mix of	SRX005988	RAL-304_75b	SRR835236	75	merged, use min length	100.0	DGRP-F2	142.5	45bp:25081192, 64bp:7071260, 75b	DGRP	F2	Blo	25177	RAL-304	SRX156009 and	252.1	252,108,056	133.6	8
13	DGRP_304	Bloomington-25177	inbred line, mix of	SRX005988	RAL-304_100b	SRR835236	100	merged, use min length	100.0	DGRP-F2	142.5	45bp:25081192, 64bp:7071260, 75b	DGRP	F2	Blo	25177	RAL-304	SRX156009 and	252.1	252,108,056	133.6	8
14	DGRP_304	Bloomington-25177	inbred line, mix of	SRX005988	RAL-304_125b	SRR835236	125	merged, use min length	100.0	DGRP-F2	142.5	45bp:25081192, 64bp:7071260, 75b	DGRP	F2	Blo	25177	RAL-304	SRX156009 and	252.1	252,108,056	133.6	8
15	DGRP_306	Bloomington-37525	inbred line, mix of	SRX006140	RAL-306_75b	SRR835242	75	merged, use min length	125.0	DGRP-F2	63.2	45bp:29368112, 64bp:8510324, 75b	DGRP	F2	Blo	37525	RAL-306	SRX156007 and	100.4	100,446,332	50.7	9
16	DGRP_306	Bloomington-37525	inbred line, mix of	SRX006140	RAL-306_125b	SRR835242	125	merged, use min length	125.0	DGRP-F2	63.2	45bp:29368112, 64bp:8510324, 75b	DGRP	F2	Blo	37525	RAL-306	SRX156007 and	100.4	100,446,332	50.7	9
17	DGRP_307	Bloomington-25179	inbred line, mix of	SRX006186	RAL-307_75b	SRR835247	75	merged, use min length	75.0	DGRP-F2	144.8	36bp:9641364, 45bp:17304248, 75b	DGRP	F2	Blo	25179	RAL-307	SRX156012 and	252.2	252,236,309	142.1	10
18	DGRP_307	Bloomington-25179	inbred line, mix of	SRX006186	RAL-307_100b	SRR835247	100	merged, use min length	100.0	DGRP-F2	144.8	36bp:9641364, 45bp:17304248, 75b	DGRP	F2	Blo	25179	RAL-307	SRX156012 and	252.2	252,236,309	142.1	10
19	DGRP_307	Bloomington-25179	inbred line, mix of	SRX006186	RAL-307_125b	SRR835247	125	merged, use min length	125.0	DGRP-F2	144.8	36bp:9641364, 45bp:17304248, 75b	DGRP	F2	Blo	25179	RAL-307	SRX156012 and	252.2	252,236,309	142.1	10
20	DGRP_315	Bloomington-25181	inbred line, mix of	SRX006143	RAL-315_75b	SRR835252	75	merged, use min length	125.0	DGRP-F2	63.1	45bp:48481060, 75bp:20407822, 85	DGRP	F2	Blo	25181	RAL-315	SRX156010 and	114.4	114,404,766	51	11
21	DGRP_315	Bloomington-25181	inbred line, mix of	SRX006143	RAL-315_125b	SRR835252	125	merged, use min length	125.0	DGRP-F2	63.1	45bp:48481060, 75bp:20407822, 85	DGRP	F2	Blo	25181	RAL-315	SRX156010 and	114.4	114,404,766	51	11
22	DGRP_340	Bloomington-28174	inbred line, mix of	SRX156030	RAL-340_100b	SRR835939	100	merged, use min length	100.0	DGRP-F2	103.7	75bp:41788984, 100bp:165697712	DGRP	F2	Blo	28174	RAL-340	SRX156030	130.2	130,158,488	73.8	12
23	DGRP_371	Bloomington-28183	inbred line, mix of	SRX021257	RAL-371_75b	SRR835326	75	merged, use min length	125.0	DGRP-F1 + F2	42.6	75bp:30869276, 125bp:27187181	DGRP	F1 + F2	Blo	28183	RAL-371	SRX021257 and	78.6	78,638,404	42.5	13
24	DGRP_371	Bloomington-28183	inbred line, mix of	SRX021257	RAL-371_125b	SRR835326	125	merged, use min length	125.0	DGRP-F1 + F2	42.6	75bp:30869276, 125bp:27187181	DGRP	F1 + F2	Blo	28183	RAL-371	SRX021257 and	78.6	78,638,404	42.5	13
25	DGRP_373	Bloomington-28184	inbred line, mix of	SRX023425	RAL-373_95b	SRR835329	95	merged, use min length	125.0	DGRP-F1	42.8	95bp:40216244, 125bp:45373906	DGRP	F1	Blo	28184	RAL-373	SRX023425 and	76.7	76,690,462	48.3	14
26	DGRP_373	Bloomington-28184	inbred line, mix of	SRX023425	RAL-373_125b	SRR835329	125	merged, use min length	125.0	DGRP-F1	42.8	95bp:40216244, 125bp:45373906	DGRP	F1	Blo	28184	RAL-373	SRX023425 and	76.7	76,690,462	48.3	14
27	DGRP_409	Bloomington-28278	inbred line, mix of	SRX021243	RAL-409_95b	SRR835331	95	merged, use min length	125.0	DGRP-F1	38.7	95bp:33305170, 125bp:44122246	DGRP	F1	Blo	28278	RAL-409	SRX021243 and	55.1	55,099,676	35.7	15
28	DGRP_409	Bloomington-28278	inbred line, mix of	SRX021243	RAL-409_125b	SRR835331	125	merged, use min length	125.0	DGRP-F1	38.7	95bp:33305170, 125bp:44122246	DGRP	F1	Blo	28278	RAL-409	SRX021243 and	55.1	55,099,676	35.7	15
29	DGRP_584	Bloomington-28212	inbred line, mix of	SRX155987	RAL-584_95b	SRR835942	95	merged, use min length	125.0	DGRP-F2	70	95bp:93885100, 125bp:46038390	DGRP	F2	Blo	28212	RAL-584	SRX155987 and	79.7	79,663,962	49.4	16
30	DGRP_584	Bloomington-28212	inbred line, mix of	SRX155987	RAL-584_125b	SRR835942	125	merged, use min length	125.0	DGRP-F2	70	95bp:93885100, 125bp:46038390	DGRP	F2	Blo	28212	RAL-584	SRX155987 and	79.7	79,663,962	49.4	16
31	DGRP_748	Bloomington-28224	inbred line, mix of	SRX156019	RAL-748_95b	SRR835336	95	merged, use min length	125.0	DGRP-F2	43.7	95bp:36957084, 125bp:50455402	DGRP	F2	Blo	28224	RAL-748	SRX156019 and	57.4	57,410,828	38.3	17
32	DGRP_748	Bloomington-28224	inbred line, mix of	SRX156019	RAL-748_125b	SRR835336	125	merged, use min length	125.0	DGRP-F2	43.7	95bp:36957084, 125bp:50455402	DGRP	F2	Blo	28224	RAL-748	SRX156019 and	57.4	57,410,828	38.3	17
33	DGRP_801	Bloomington-28234	inbred line, mix of	SRX021391	RAL-801_95b	SRR835341	95	merged, use min length	125.0	DGRP-F1	43.4	95bp:32922146, 125bp:53899816	DGRP	F1	Blo	28234	RAL-801	SRX021391 and	59.3	59,280,892	38.4	18
34	DGRP_801	Bloomington-28234	inbred line, mix of	SRX021391	RAL-801_125b	SRR835341	125	merged, use min length	125.0	DGRP-F1	43.4	95bp:32922146, 125bp:53899816	DGRP	F1	Blo	28234	RAL-801	SRX021391 and	59.3	59,280,892	38.4	18
35	DGRP_802	Bloomington-28235	inbred line, mix of	SRX025318	RAL-802_95b	SRR835343	95	merged, use min length	125.0	DGRP-F1	47.1	95bp:27114342, 125bp:67142296	DGRP	F1	Blo	28235	RAL-802	SRX025318 and	78.6	78,579,930	52.4	19
36	DGRP_802	Bloomington-28235	inbred line, mix of	SRX025318	RAL-802_125b	SRR835343	125	merged, use min length	125.0	DGRP-F1	47.1	95bp:27114342, 125bp:67142296	DGRP	F1	Blo	28235	RAL-802	SRX025318 and	78.6	78,579,930	52.4	19
37	DGRP_808	Bloomington-28238	inbred line, mix of	SRX021402	RAL-808_75b	SRR835345	75	merged, use min length	125.0	DGRP-F1	53.8	75bp:44482188, 125bp:63083538	DGRP	F1	Blo	28238	RAL-808	SRX021402 and	83.0	82,954,992	49.3	20
38	DGRP_808	Bloomington-28238	inbred line, mix of	SRX021402	RAL-808_125b	SRR835345	125	merged, use min length	125.0	DGRP-F1	53.8	75bp:44482188, 125bp:63083538	DGRP	F1	Blo	28238	RAL-808	SRX021402 and	83.0	82,954,992	49.3	20
39	DGRP_821	Bloomington-28243	inbred line, mix of	SRX155991	RAL-821_100b	SRR835347	100	merged, use min length	100.0	DGRP-F2	108.2	100bp:132570414, 125bp:83747778	DGRP	F2	Blo	28243	RAL-821	SRX155990 and	179.3	179,277,520	112	21
40	DGRP_821	Bloomington-28243	inbred line, mix of	SRX155991	RAL-821_125b	SRR835347	125	merged, use min length	100.0	DGRP-F2	108.2	100bp:132570414, 125bp:83747778	DGRP	F2	Blo	28243	RAL-821	SRX155990 and	179.3	179,277,520	112	21
41	DGRP_853	Bloomington-28250	inbred line, mix of	SRX021491	RAL-853_110b	SRR835349	110	merged, use min length	125.0	DGRP-F2	55.3	110bp:42021476, 125bp:68611046	DGRP	F2	Blo	28250	RAL-853	SRX021491 and	69.8	69,801,922	48.9	22
42	DGRP_853	Bloomington-28250	inbred line, mix of	SRX021491	RAL-853_125b	SRR835349	125	merged, use min length	125.0	DGRP-F2	55.3	110bp:42021476, 125bp:68611046	DGRP	F2	Blo	28250	RAL-853	SRX021491 and	69.8	69,801,922	48.9	22
43	DGRP_900	Bloomington-28261	inbred line, mix of	SRX156023	RAL-900_95b	SRR846989	95	merged, use min length	125.0	DGRP-F2	47.6	95bp:30160598, 125bp:65136580	DGRP	F2	Blo	28261	RAL-900	SRX156023	77.4	77,355,512	52.3	23
44	DGRP_900	Bloomington-28261	inbred line, mix of	SRX156023	RAL-900_125b	SRR846989	125	merged, use min length	125.0	DGRP-F2	47.6	95bp:30160598, 125bp:65136580	DGRP	F2	Blo	28261	RAL-900	SRX156023	77.4	77,355,512	52.3	23
45	DGRP_382	Bloomington-28189	inbred line, mix of	SRX156013	RAL-382	SRR834552	125	125	125	DGRP-F2	36.9	125bp:73812254	DGRP	F2	Blo	28189	RAL-382	SRX156013	57.6	57,592,186	41.1	24
46	DGRP_223	Bloomington-28155	inbred line, mix of	SRX155994	RAL-223	SRR834527	125	125	125	DGRP-F2	35.6	125bp:71152512	DGRP	F2	Blo	28155	RAL-223	SRX155994	57.2	57,181,894	40.8	25
47	DGRP_850	Bloomington-28249	inbred line, mix of	SRX155993	RAL-850	SRR835061	125	125	125	DGRP-F2	34.8	125bp:69699750	DGRP	F2	Blo	28249	RAL-850	SRX155993	61.0	60,987,804	43.6	26
48	DGRP_913	Bloomington-28265	inbred line, mix of	SRX156024	RAL-913	SRR835077	125	125	125	DGRP-F2	34.6	125bp:69250292	DGRP	F2	Blo	28265	RAL-913	SRX156024	61.2	61,221,716	43.7	27
49	DGRP_843	Bloomington-28247	inbred line, mix of	SRX156036	RAL-843	SRR835059	125	125	125	DGRP-F2	34.3	125bp:68658714	DGRP	F2	Blo	28247	RAL-843	SRX156036	59.2	59,174,050	42.3	28
50	DGRP_361	Bloomington-28180	inbred line, mix of	SRX155984	RAL-361	SRR834553	125	125	125	DGRP-F2	34.1	125bp:68254340	DGRP	F2	Blo	28180	RAL-361	SRX155984	56.9	56,897,940	40.6	29
51	DGRP_189	Bloomington-28152	inbred line, mix of	SRX155979	RAL-189	SRR834523	125	125	125	DGRP-F2	31.6	125bp:63289120	DGRP	F2	Blo	28152	RAL-189	SRX155979	52.9	52,919,862	37.8	30
52	DGRP_849	Bloomington-28248	inbred line, mix of	SRX156035	RAL-849	SRR835060	125	125	125	DGRP-F2	30.8	125bp:61687178	DGRP	F2	Blo	28248	RAL-849	SRX156035	55.8	55,797,340	39.9	31
53	DGRP_142	Bloomington-28144	inbred line, mix of	SRX020759	RAL-142	SRR834551	100	110	110	DGRP-F1	20.6	110bp:41167794	DGRP	F1	Blo	28144	RAL-142	SRX020759	31.4	31,409,058	19.7	32
54	DGRP_822	Bloomington-28244	inbred line, mix of	SRX021476	RAL-822	SRR835055	100	110	110	DGRP-F1	20.5	110bp:41079524	DGRP	F1	Blo	28244	RAL-822	SRX021476	28.1	28,098,026	17.7	33
55	DGRP_75	Bloomington-28132	inbred line, mix of	SRX021384	RAL-75	SRR835087	100	110	110	DGRP-F1	19.1	110bp:38161744	DGRP	F1	Blo	28132	RAL-75	SRX021384	29.5	29,460,700	18.5	34
56	DGRP_395	Bloomington-28194	inbred line, mix of	SRX156015	RAL-395	SRR834521	100	101	101	DGRP-F2	43.6	101bp:87233368	DGRP	F2	Blo	28194	RAL-395	SRX156015	81.5	81,549,134	47.1	35
57	DGRP_819	Bloomington-28242	inbred line, mix of	SRX156006	RAL-819	SRR835054	100	100	100	DGRP-F2	75.4	100bp:150745358	DGRP	F2	Blo	28242	RAL-819	SRX156006	127.8	127,803,094	73	36
58	DGRP_57	Bloomington-29652	inbred line, mix of	SRX021296	RAL-57	SRR933581	100	100	100	DGRP-F1	32.5	100bp:64966990	DGRP	F1	Blo	29652	RAL-57	SRX021296	57.0	57,017,248	32.6	37
59	DGRP_138	Bloomington-28143	inbred line, mix of	SRX021008	RAL-138	SRR932121	100	100	100	DGRP-F1	30.8	100bp:61689820	DGRP	F1	Blo	28143	RAL-138	SRX021008	52.6	52,639,192	30.1	38
60	DGRP_324	Bloomington-25182	inbred line, mix of	SRX006145	RAL-324_100	SRR835256	100	merged, use min length	100	DGRP-F2	127.3	45bp:79789940, 100bp:174874844	DGRP	F2	Blo	25182	RAL-324	SRX006144 and	233.9	233,891,168	109.2	39
61	DGRP_360	Bloomington-25186	inbred line, mix of	SRX155999	RAL-360_125	SRR835260	125	merged, use min length	125	DGRP-F2	87.3	36bp:59319927, 125bp:55959646	DGRP	F2	Blo	25186	RAL-360	SRX155999 and	85.6	85,563,461	38.2	40
62	DGRP_437	Bloomington-25194	inbred line, mix of	SRX006156	RAL-437_125	SRR835333	125	merged, use min length	125	DGRP-F1	46.9	45bp:43208064, 125bp:50502476	DGRP	F1	Blo	25194	RAL-437	SRX006156 and	83.9	83,861,754	40.4	41
63	DGRP_774	Bloomington-25205	inbred line, mix of	SRX006170	RAL-774_125	SRR835338	125	merged, use min length	125	DGRP-F1	38.2	45bp:42434168, 125bp:33991884	DGRP	F1	Blo	25205	RAL-774	SRX006170 and	68.9	68,894,652	30	42
64	DGRP_391A	Bloomington-25191	inbred line, mix of	SRX006152	RAL-391_75b	SRR018294	75	merged, use min length	75	DGRP-F1 + F2	9.5	45bp:40955170, 75bp:18124720, 95	DGRP	F1 + F2	Blo	25191	RAL-391	SRX023452,SR	95.3	95,313,294	38	43
65	DGRP_391B	Bloomington-25191	inbred line, mix of	SRX023452	RAL-391_95b	SRR060098	95	merged, use min length	95	DGRP-F1 + F2	19	45bp:40955170, 75bp:18124720, 95	DGRP	F1 + F2	Blo	25191	RAL-391	SRX023452,SR	95.3	95,313,294	38	44
66	DGRP_380	Bloomington-25190	inbred line, mix of	SRX006300	RAL-380	SRR018591, SR	75	merged, use min length	75	DGRP-F1 + F2	10.2, 10.3	45bp:505064, 74bp:20092700, 75bp	DGRP	F1 + F2	Blo	25190	RAL-380	SRX006303,SR	69.2	69,152,136	29.5	45
67	DGRP_176	Bloomington-28149	inbred line, mix of	SRX020762, SR	RAL-176	SRR048925, SR	75	75	75	DGRP-F1	19.7, 20.3	75bp:79996860	DGRP	F1	Blo	28149	RAL-176	SRX020762, SR	73.7	73,734,058	31.6	46

69	DGRP_358	Bloomington-25185	inbred line, mix of	SRX006282, SR	RAL-358	SRR018574, SR	75.0	merged, use min length	75.0	DGRP-F1	6.8, 14.1	45bp:18011532, 75bp:27739857	DGRI	F1	Blo	25185	RAL-358	SRX006283 and	42.5	42,499,891	15.2	47	
70	DGRP_399	Bloomington-25192	inbred line, mix of	SRX006153	RAL-399	SRR933575	75.0	merged, use min length	75.0	DGRP-F1	27.8	45bp:41011932, 75bp:14665138	DGRI	F1	Blo	25192	RAL-399	SRX006154 and	51.6	51,632,558	15.5	48	
71	DGRP_313	Bloomington-25180	inbred line, mix of	SRX006277	RAL-313	SRR018519	75.0	single end only	75.0	DGRP-F1 + F2	14	36bp:9477876, 45bp:18165034, 75b	DGRI	F1 + F2	Blo	25180	RAL-313	SRX006277,SR	39.1	39,060,649	11.7	49	
72	DGRP_375	Bloomington-25188	inbred line, mix of	SRX006148	RAL-375	SRR933572	75.0	merged, use min length	75.0	DGRP-F1	51.8	45bp:52106222, 75bp:51413814	DGRI	F1	Blo	25188	RAL-375	SRX006150,SR	99.3	99,258,956	33.8	50	
73	DGRP_555	Bloomington-25198	inbred line, mix of	SRX006159	RAL-555	SRR933580	75	75	75	DGRP-F1	25.1	75bp:50103810	DGRI	F1	Blo	25198	RAL-555	SRX006159	44.9	44,883,398	19.2	51	
74	DGRP_705	Bloomington-25744	inbred line, mix of	SRX006162	RAL-705	SRR933585	75	75	75	DGRP-F1	23.5	75bp:47006608	DGRI	F1	Blo	25744	RAL-705	SRX006162	38.9	38,920,070	16.7	52	
75	DGRP_707	Bloomington-25200	inbred line, mix of	SRX006163	RAL-707	SRR933586	75	75	75	DGRP-F1	23.3	75bp:46657404	DGRI	F1	Blo	25200	RAL-707	SRX006163	41.6	41,568,952	17.8	53	
76	DGRP_712	Bloomington-25201	inbred line, mix of	SRX006164	RAL-712	SRR933587	75	75	75	DGRP-F1	22.3	75bp:44687868	DGRI	F1	Blo	25201	RAL-712	SRX006164	37.9	37,949,898	16.3	54	
77	DGRP_714	Bloomington-25745	inbred line, mix of	SRX006166	RAL-714	SRR933588	75.0	75.0	75.0	DGRP-F1	21	75bp:42090922	DGRI	F1	Blo	25745	RAL-714	SRX006166 and	40.0	39,955,902	17.1	55	
78	DGRP_732	Bloomington-25203	inbred line, mix of	SRX006167	RAL-732	SRR933591	75	75	75	DGRP-F1	21.1	75bp:42170344	DGRI	F1	Blo	25203	RAL-732	SRX006167	38.1	38,082,012	16.3	56	
79	DGRP_379	Bloomington-25189	inbred line, mix of	SRX006293	RAL-379	SRR018583, SR	75.0	single end only	75.0	DGRP-F1	6.1, 12.4	45bp:19006960, 75bp:18496338	DGRI	F1	Blo	25189	RAL-379	SRX006293 and	34.0	34,014,707	11.4	57	
80	DGRP_340	Bloomington-28174	inbred line, mix of	SRX156030	RAL-340_75b	SRR835939	75	merged, use min length	100.0	DGRP-F2	103.7	75bp:41788984, 100bp:165697712	DGRI	F2	Blo	28174	RAL-340	SRX156030	130.2	130,158,488	73.8	12	This entry repeatedly gen

PHASE 2: Additional DGRP Illumina-sequenced libaries compatible for TIDAL analysis loaded into TIDAL-Fly database but not part of analysis in paper.

Library	Stock ID*	Stock Location	Genome Type	SRA Accession	Library ID*	SRA Accession	Min Read Length	Read Length 1	Read Length 2	Data Group	Reads (M)	Raw Read Length:Read Number		Freeze		Bloomington Stock	Synonym	NCBI SRA	Reads	Mapped Reads	Mapped Coverage	Strains
1	DGRP_365	Bloomington-25445	inbred line, mix of	SRX006290, SR	RAL-365	SRR018580, SRR	75	75.0	75.0	DGRP-F1	10.1	45bp:18394996, 75bp:32783196	DGRI	F1	Blo	25445	RAL-365	SRX006291,SR	42.9	42,859,487	15.6	58
3	DGRP_852	Bloomington-25209	inbred line, mix of	SRX006304, SR	RAL-852	SRR018600	75	75.0	75.0	DGRP-F1	11.7	45bp:41788724, 75bp:59375261	DGRI	F1	Blo	25209	RAL-852	SRX006304 and	90.6	90,552,271	32.2	59
7	DGRP_730	Bloomington-25202	inbred line, mix of	SRX006308	RAL-730	SRR933590	75	75	75	DGRP-F1	38	75bp:38019145	DGRI	F1	Blo	25202	RAL-730	SRX006308	30.9	30,891,608	13.2	60
8	DGRP_738	Bloomington-28223	inbred line, mix of	SRX021383	RAL-738	SRR933593	75	75	75	DGRP-F1	37.9	75bp:75804508	DGRI	F1	Blo	28223	RAL-738	SRX021383	63.1	63,119,910	27.1	61
9	DGRP_737	Bloomington-28222	inbred line, mix of	SRX023451	RAL-737	SRR933592	75	75	75	DGRP-F1	37.4	75bp:74740132	DGRI	F1	Blo	28222	RAL-737	SRX023451	58.5	58,531,732	25.1	62
10	DGRP_757	Bloomington-28226	inbred line, mix of	SRX021385	RAL-757	SRR933594	75	75	75	DGRP-F1	37.2	75bp:74326240	DGRI	F1	Blo	28226	RAL-757	SRX021385	66.2	66,207,766	28.4	63
11	DGRP_727	Bloomington-28221	inbred line, mix of	SRX021382	RAL-727	SRR933589	75	75	75	DGRP-F1	36.9	75bp:73781476	DGRI	F1	Blo	28221	RAL-727	SRX021382	64.2	64,202,850	27.5	64
12	DGRP_897	Bloomington-28260	inbred line, mix of	SRX023457	RAL-897	SRR933601	75	75	75	DGRP-F1	35.4	75bp:70892788	DGRI	F1	Blo	28260	RAL-897	SRX023457	63.0	63,033,690	27	65
13	DGRP_332	Bloomington-28171	inbred line, mix of	SRX021095	RAL-332	SRR933569	75	75	75	DGRP-F1	32.8	75bp:65583082	DGRI	F1	Blo	28171	RAL-332	SRX021095	60.0	59,985,078	25.7	66
14	DGRP_181	Bloomington-28151	inbred line, mix of	SRX020912	RAL-181	SRR933563	75	75	75	DGRP-F1	32	75bp:64093862	DGRI	F1	Blo	28151	RAL-181	SRX020912	57.6	57,566,276	24.7	67
15	DGRP_325	Bloomington-28170	inbred line, mix of	SRX021793	RAL-325	SRR933568	75	75	75	DGRP-F1	31.5	75bp:31457046	DGRI	F1	Blo	28170	RAL-325	SRX021793	26.3	26,299,389	11.3	68
16	DGRP_381	Bloomington-28188	inbred line, mix of	SRX021112	RAL-381	SRR933573	75	75	75	DGRP-F1	27.2	75bp:54335852	DGRI	F1	Blo	28188	RAL-381	SRX021112	48.7	48,708,464	20.9	69
17	DGRP_153	Bloomington-28146	inbred line, mix of	SRX021514	RAL-153	SRR933562	75	75	75	DGRP-F1	26.8	75bp:26821499	DGRI	F1	Blo	28146	RAL-153	SRX021514	21.8	21,768,014	9.3	70
18	DGRP_352	Bloomington-28177	inbred line, mix of	SRX021101	RAL-352	SRR834516	75	75	75	DGRP-F1	22.5	75bp:44982388	DGRI	F1	Blo	28177	RAL-352	SRX021101	36.3	36,302,672	15.6	71
19	DGRP_882	Bloomington-28255	inbred line, mix of	SRX021496	RAL-882	SRR835067	75	75	75	DGRP-F1	22.4	75bp:44722234	DGRI	F1	Blo	28255	RAL-882	SRX021496	40.6	40,585,544	17.4	72
20	DGRP_879	Bloomington-28254	inbred line, mix of	SRX021494	RAL-879	SRR835066	75	75	75	DGRP-F1	21.7	75bp:43485194	DGRI	F1	Blo	28254	RAL-879	SRX021494	30.5	30,475,860	13.1	73
21	DGRP_805	Bloomington-28237	inbred line, mix of	SRX021400	RAL-805	SRR835095	75	75	75	DGRP-F1	21.6	75bp:43182102	DGRI	F1	Blo	28237	RAL-805	SRX021400	37.7	37,672,090	16.1	74
22	DGRP_356	Bloomington-28178	inbred line, mix of	SRX023833	RAL-356	SRR834537	75	75	75	DGRP-F1	21.5	75bp:42903612	DGRI	F1	Blo	28178	RAL-356	SRX023833	36.1	36,113,766	15.5	75
23	DGRP_884	Bloomington-28256	inbred line, mix of	SRX021498	RAL-884	SRR835068	75	75	75	DGRP-F1	21.3	75bp:42658114	DGRI	F1	Blo	28256	RAL-884	SRX021498	32.6	32,585,778	14	76
24	DGRP_890	Bloomington-28257	inbred line, mix of	SRX021499	RAL-890	SRR835071	75	75	75	DGRP-F1	21.0	75bp:41954706	DGRI	F1	Blo	28257	RAL-890	SRX021499	37.1	37,101,876	15.9	77
25	DGRP_861	Bloomington-28253	inbred line, mix of	SRX021493	RAL-861	SRR835065	75	75	75	DGRP-F1	20.6	75bp:41295320	DGRI	F1	Blo	28253	RAL-861	SRX021493	29.5	29,478,054	12.6	78
26	DGRP_83	Bloomington-28134	inbred line, mix of	SRX023456	RAL-83	SRR835058	75	75	75	DGRP-F1	20.5	75bp:41070470	DGRI	F1	Blo	28134	RAL-83	SRX023456	38.0	37,982,090	16.3	79
27	DGRP_491	Bloomington-28202	inbred line, mix of	SRX021268	RAL-491	SRR835035	75	75	75	DGRP-F1	20.5	75bp:40944392	DGRI	F1	Blo	28202	RAL-491	SRX021268	35.3	35,336,140	15.1	80
28	DGRP_761	Bloomington-28227	inbred line, mix of	SRX021386	RAL-761	SRR835088	75	75	75	DGRP-F1	20.4	75bp:40867250	DGRI	F1	Blo	28227	RAL-761	SRX021386	35.6	35,577,354	15.2	81
29	DGRP_149	Bloomington-28145	inbred line, mix of	SRX020760	RAL-149	SRR834550	75	75	75	DGRP-F1	20.3	75bp:40637646	DGRI	F1	Blo	28145	RAL-149	SRX020760	33.9	33,914,600	14.5	82
30	DGRP_535	Bloomington-28208	inbred line, mix of	SRX021293	RAL-535	SRR835046	75	75	75	DGRP-F1	20.1	75bp:40234802	DGRI	F1	Blo	28208	RAL-535	SRX021293	35.4	35,362,252	15.2	83
31	DGRP_776	Bloomington-28229	inbred line, mix of	SRX021387	RAL-776	SRR835089	75	75	75	DGRP-F1	19.9	75bp:39890986	DGRI	F1	Blo	28229	RAL-776	SRX021387	36.4	36,432,512	15.6	84
32	DGRP_787	Bloomington-28231	inbred line, mix of	SRX021388	RAL-787	SRR835091	75	75	75	DGRP-F1	19.9	75bp:39795416	DGRI	F1	Blo	28231	RAL-787	SRX021388	35.9	35,896,250	15.4	85
33	DGRP_136	Bloomington-28142	inbred line, mix of	SRX020753	RAL-136	SRR834542	75	75	75	DGRP-F1	19.5	75bp:38970964	DGRI	F1	Blo	28142	RAL-136	SRX020753	33.9	33,947,560	14.5	86
34	DGRP_318	Bloomington-28168	inbred line, mix of	SRX021082	RAL-318	SRR834507	75	75	75	DGRP-F1	19.5	75bp:39068236	DGRI	F1	Blo	28168	RAL-318	SRX021082	35.4	35,366,996	15.2	87
35	DGRP_804	Bloomington-28236	inbred line, mix of	SRX021399	RAL-804	SRR835094	75	75	75	DGRP-F1	19.5	75bp:38910810	DGRI	F1	Blo	28236	RAL-804	SRX021399	34.5	34,482,650	14.8	88
36	DGRP_812	Bloomington-28240	inbred line, mix of	SRX021419	RAL-812	SRR835052	75	75	75	DGRP-F1	19.4	75bp:38719004	DGRI	F1	Blo	28240	RAL-812	SRX021419	37.5	37,540,214	16.1	89
37	DGRP_796	Bloomington-28233	inbred line, mix of	SRX021390	RAL-796	SRR835093	75	75	75	DGRP-F1	19.3	75bp:38662002	DGRI	F1	Blo	28233	RAL-796	SRX021390	34.7	34,681,678	14.9	90
38	DGRP_509	Bloomington-28206	inbred line, mix of	SRX021273	RAL-509	SRR835041	75	75	75	DGRP-F1	19.0	75bp:38095912	DGRI	F1	Blo	28206	RAL-509	SRX021273	35.6	35,615,726	15.3	91
39	DGRP_818	Bloomington-28241	inbred line, mix of	SRX021478	RAL-818	SRR835053	75	75	75	DGRP-F1	18.8	75bp:37531016	DGRI	F1	Blo	28241	RAL-818	SRX021478	34.2	34,210,842	14.7	92
40	DGRP_859	Bloomington-25210	inbred line, mix of	SRX006176	RAL-859	SRR933600	75	75	75	DGRP-F1	18.5	75bp:36919488	DGRI	F1	Blo	25210	RAL-859	SRX006176	34.0	33,950,076	14.6	93
41	DGRP_810	Bloomington-28239	inbred line, mix of	SRX021418	RAL-810	SRR835051	75	75	75	DGRP-F1	18.5	75bp:36972402	DGRI	F1	Blo	28239	RAL-810	SRX021418	36.1	36,075,996	15.5	94
42	DGRP_783	Bloomington-28230	inbred line, mix of	SRX023455	RAL-783	SRR835090	75	75	75	DGRP-F1	18.3	75bp:36692434	DGRI	F1	Blo	28230	RAL-783	SRX023455	31.4	31,444,902	13.5	95
43	DGRP_45	Bloomington-28128	inbred line, mix of	SRX021261	RAL-45	SRR835032	75	75	75	DGRP-F1	18.2	75bp:36472946	DGRI	F1	Blo	28128	RAL-45	SRX021261	33.0	32,984,934	14.1	96
44	DGRP_59	Bloomington-28129	inbred line, mix of	SRX021327	RAL-59	SRR835081	75	75	75	DGRP-F1	18.2	75bp:36484216	DGRI	F1	Blo	28129	RAL-59	SRX021327	34.9	34,871,104	14.9	97
45	DGRP_280	Bloomington-28164	inbred line, mix of	SRX021058	RAL-280	SRR834534	75	75	75	DGRP-F1	17.8	75bp:35581218	DGRI	F1	Blo	28164	RAL-280	SRX021058	33.0	32,968,684	14.1	98
46	DGRP_595	Bloomington-28215	inbred line, mix of	SRX021328	RAL-595	SRR835101	75	75	75	DGRP-F1	17.8	75bp:35536106	DGRI	F1	Blo	28215	RAL-595	SRX021328	29.2	29,176,032	12.5	99
47	DGRP_93	Bloomington-28137	inbred line, mix of	SRX021504	RAL-93	SRR835080	75	75	75	DGRP-F1	17.6	75bp:35127632	DGRI	F1	Blo	28137	RAL-93	SRX021504	33.9	33,871,000	14.5	100
48	DGRP_195	Bloomington-28153	inbred line, mix of	SRX021039	RAL-195	SRR834524	75	75	75	DGRP-F1	17.6	75bp:35114464	DGRI	F1	Blo	28153	RAL-195	SRX021039	33.4	33,435,584	14.3	101
49	DGRP_287	Bloomington-28165	inbred line, mix of	SRX021059	RAL-287	SRR834535	75	75	75	DGRP-F1	17.6	75bp:35285772	DGRI	F1	Blo	28165	RAL-287	SRX021059	22.8	22,778,884	9.8	102
50	DGRP_101	Bloomington-28138	inbred line, mix of	SRX020747	RAL-101	SRR834536	75	75	75	DGRP-F1	17.4	75bp:34790704	DGRI	F1	Blo	28138	RAL-101	SRX020747	28.6	28,625,600	12.3	103
51	DGRP_256	Bloomington-28162	inbred line, mix of	SRX021055	RAL-256	SRR834533	75	75	75	DGRP-F1	17.4	75bp:34717354	DGRI	F1	Blo	28162	RAL-256	SRX021055	32.6	32,570,608	14	104
52	DGRP_239	Bloomington-28161	inbred line, mix of	SRX021054	RAL-239	SRR834532	75	75	75	DGRP-F1	17.2	75bp:34456900	DGRI	F1	Blo	28161	RAL-239	SRX021054	33.0	32,977,516	14.1	105
53	DGRP_642	Bloomington-28216	inbred line, mix of	SRX021331	RAL-642	SRR835082	75	75	75	DGRP-F1	17.1	75bp:34225882	DGRI	F1	Blo	28216	RAL-642	SRX021331	32.7	32,678,408	14	106
54	DGRP_85	Bloomington-28274	inbred line, mix of	SRX021490	RAL-85	SRR835064	75	75	75	DGRP-F1	17.1	75bp:34278668	DGRI	F1	Blo	28274	RAL-85	SRX021490	32.8	32,816,544	14.1	107
55	DGRP_309	Bloomington-28166	inbred line, mix of	SRX021060	RAL-309	SRR834504	75	75	75	DGRP-F1	17.0	75bp:34062874	DGRI	F1	Blo	28166	RAL-309	SRX021060	31.0	30,967,892	13.3	108
56	DGRP_563	Bloomington-28211	inbred line, mix of	SRX023836	RAL-563	SRR835049	75	75	75	DGRP-F1	17.0	75bp:33949618	DGRI	F1	Blo	28211	RAL-563	SRX023836	32.7	32,699,510	14	109
57	DGRP_69	Bloomington-28130	inbred line, mix of	SRX023449	RAL-69	SRR933583	75	75	75	DGRP-F1	16.9	75bp:33850154	DGRI	F1	Blo	28130	RAL-69	SRX023449	31.3	31,330,920	13.4	110
58	DGRP_161	Bloomington-28148	inbred line, mix of	SRX020761	RAL-161	SRR834548	75	75	75	DGRP-F1	16.8	75bp:33569140	DGRI	F1	Blo	28148	RAL-161	SRX020761	31.9	31,889,608	13.7	111
59	DGRP_721	Bloomington-28220	inbred line, mix of	SRX021381	RAL-721	SRR835099	75	75	75	DGRP-F1	16.8	75bp:33513644	DGRI	F1	Blo	28220	RAL-721	SRX021381	29.3	29,343,228	12.6	112
60	DGRP_310	Bloomington-28276	inbred line, mix of	SRX021080	RAL-310	SRR834505	75	75	75	DGRP-F1	16.8	75bp:33591214,	DGRI	F1	Blo	28276	RAL-310	SRX021080	29.3	29,276,428	12.5	113
61	DGRP_73	Bloomington-28131	inbred line, mix of	SRX023450	RAL-73	SRR835100	75	75	75	DGRP-F1	16.6	75bp:33134294	DGRI	F1	Blo	28131	RAL-73	SRX023450	31.7	31,685,126	13.6	114
62	DGRP_91	Bloomington-28136	inbred line, mix of	SRX021503	RAL-91	SRR835078	75	75	75	DGRP-F1	16.6	75bp:33211530	DGRI	F1	Blo	28136	RAL-91	SRX021503	31.5	31,458,484	13.5	115
63	DGRP_367	Bloomington-28181	inbred line, mix of	SRX021103	RAL-367	SRR834544	75	75	75	DGRP-F1	16.4	75bp:32780338	DGRI	F1	Blo	28181	RAL-367	SRX021103	30.6	30,576,784	13.1	116
64	DGRP_336	Bloomington-28172	inbred line, mix of	SRX021096	RAL-336	SRR933570	75	75	75	DGRP-F2	16.3	75bp:32559800	DGRI	F2	Blo	28172	RAL-336	SRX021096	26.5	26,524,196	11.4	117
65	DGRP_646	Bloomington-28217	inbred line, mix of	SRX021332	RAL-646	SRR835083	75	75	75	DGRP-F1	16.2	75bp:32350764	DGRI	F1	Blo	28217	RAL-646	SRX021332	28.7	28,739,440	12.3	118
66	DGRP_88	Bloomington-28135	inbred line, mix of	SRX021495	RAL-88	SRR835070	75	75	75	DGRP-F1	16.0	75bp:32012994	DGRI	F1	Blo	28135	RAL-88	SRX021495	30.9	30,899,746	13.2	119
67	DGRP_105	Bloomington-28139	inbred line, mix of	SRX020745	RAL-105	SRR834538	75	75	75	DGRP-F1	15.9	75bp:31797740	DGRI	F1	Blo	28139	RAL-105	SRX020745	30.8	30,788,030	13.2	120
68	DGRP_716	Bloomington-28219	inbred line, mix of	SRX021380	RAL-716	SRR835085	75	75	75	DGRP-F1	15.8	75bp:31572156	DGRI	F1	Blo	28219	RAL-716	SRX021380	29.2	29,244,674	12.5	121
69	DGRP_217	Bloomington-28154	inbred line, mix of	SRX021041	RAL-217	SRR834525	75	75	75	DGRP-F1	15.7	75bp:31354632	DGRI	F1	Blo	28154	RAL-217	SRX021041	29.9	29,857,218	12.8	122
70	DGRP_158	Bloomington-28147	inbred line, mix of	SRX021009	RAL-158	SRR834549	75	75	75	DGRP-F1	15.5	75bp:31050122	DGRI	F1	Blo	28147	RAL-158	SRX021009	27.6	27,629,766	11.8	123
71	DGRP_227	Bloomington-28156	inbred line, mix of	SRX021042	RAL-227	SRR834528	75	75	75	DGRP-F1	15.5	75bp:30993282	DGRI	F1	Blo	28156	RAL-227	SRX021042	27.8	27,790,122	11.9	124
72	DGRP_233	Bloomington-28159	inbred line, mix of	SRX021061	RAL-233	SRR834530	75	75	75	DGRP-F1	14.9	75bp:29795450	DGRI	F1	Blo	28159	RAL-233	SRX021061	28.4	28,358,088	12.2	125

73	DGRP_338	Bloomington-28173	inbred line, mix of	SRX021097	RAL-338	SRR834513	75	75	75	DGRP-F1	14.5	75bp:28907664	DGRI	F1	Blo	28173	RAL-338	SRX021097	26.0	25,964,076	11.1	126
74	DGRP_228	Bloomington-28157	inbred line, mix of	SRX021043	RAL-228	SRR834529	75	75	75	DGRP-F1	14.4	75bp:28874696	DGRI	F1	Blo	28157	RAL-228	SRX021043	27.3	27,339,700	11.7	127
75	DGRP_350	Bloomington-28176	inbred line, mix of	SRX021100	RAL-350	SRR834515	75	75	75	DGRP-F1	14.0	75bp:27970002	DGRI	F1	Blo	28176	RAL-350	SRX021100	25.4	25,355,594	10.9	128
76	DGRP_317	Bloomington-28167	inbred line, mix of	SRX021081	RAL-317	SRR834506	75	75	75	DGRP-F1	13.6	75bp:27246088	DGRI	F1	Blo	28167	RAL-317	SRX021081	23.3	23,252,944	10	129
77	DGRP_129	Bloomington-28141	inbred line, mix of	SRX020748	RAL-129	SRR834540	75	75	75	DGRP-F1	13.4	75bp:26898800	DGRI	F1	Blo	28141	RAL-129	SRX020748	25.8	25,797,252	11.1	130
78	DGRP_26	Bloomington-28123	inbred line, mix of	SRX021056	RAL-26	SRR933566	75	merged, use min length	75.0	DGRP-F1	18.4	44bp:4452211, 75bp:32432947	DGRI	F1	Blo	28123	RAL-26	SRX021056	35.6	35,642,606	14.5	131
79	DGRP_208	Bloomington-25174	inbred line, mix of	SRX005977	RAL-208	SRR933564	75	merged, use min length	75.0	DGRP-F1 + F2	36.3	45bp:11112096, 64bp:8693462, 75l	DGRI	F1 + F2	Blo	25174	RAL-208	SRX005977	69.8	69,801,276	27.5	132
80	DGRP_237	Bloomington-28160	inbred line, mix of	SRX023423	RAL-237	SRR933565	75	merged, use min length	75.0	DGRP-F1	29.1	44bp:15879498, 75bp:34500267	DGRI	F1	Blo	28160	RAL-237	SRX023423 and	45.0	44,995,628	16.7	133
81	DGRP_28	Bloomington-28124	inbred line, mix of	SRX021783	RAL-28	SRR933567	75	merged, use min length	75.0	DGRP-F1	26.4	44bp:15469056, 75bp:26422488	DGRI	F1	Blo	28124	RAL-28	SRX021783 and	39.1	39,097,863	14.1	134
82	DGRP_41	Bloomington-28126	inbred line, mix of	SRX021791	RAL-41	SRR933576	75	merged, use min length	75.0	DGRP-F1	24.4	44bp:14055721, 75bp:24438432	DGRI	F1	Blo	28126	RAL-41	SRX021791 and	36.1	36,082,067	13.1	135
83	DGRP_639	Bloomington-25199	inbred line, mix of	SRX006161	RAL-639	SRR933582	75	75.0	75.0	DGRP-F1	21.1	75bp:42120400	DGRI	F1	Blo	25199	RAL-639	SRX006161 and	39.0	39,017,668	16.7	136
84	DGRP_799	Bloomington-25207	inbred line, mix of	SRX006173	RAL-799	SRR933597	75	75.0	75.0	DGRP-F1	20.7	75bp:41386962	DGRI	F1	Blo	25207	RAL-799	SRX006172 and	38.9	38,942,298	16.7	137
85	DGRP_386	Bloomington-28192	inbred line, mix of	SRX021798	RAL-386	SRR933574	75	merged, use min length	75.0	DGRP-F1	20.5	44bp:11122819, 75bp:20778304	DGRI	F1	Blo	28192	RAL-386	SRX021798 and	29.6	29,626,175	10.8	138
86	DGRP_820	Bloomington-25208	inbred line, mix of	SRX006174	RAL-820	SRR933598	75	75.0	75.0	DGRP-F1	18.8	75bp:37666476	DGRI	F1	Blo	25208	RAL-820	SRX006174 and	33.9	33,910,170	14.5	139

PHASE 3: Remaining DGRP Illumina-sequenced libaries compatible for TIDAL analysis to be loaded into TIDAL-Fly database.

87	DGRP_359	Bloomington-28179	inbred line, mix of	SRX023424	RAL-359	SRR834546	95	95.0	95.0	DGRP-F1	18.6	95bp:37271884	DGRI	F1	Blo	28179	RAL-359	SRX023424	37.3	37,271,884	20.2
88	DGRP_589	Bloomington-28213	inbred line, mix of	SRX023837	RAL-589	SRR060821	75	75.0	75.0	DGRP-F1	16.5	75bp:32998374	DGRI	F1	Blo	28213	RAL-589	SRX023837	190.1	190,072,852	104.4
89	DGRP_40	Bloomington-29651	inbred line, mix of	SRX021235	RAL-40	SRR835025	95	95	95	DGRP-F1	34.5	95bp:69063428	DGRI	F1	Blo	29651	RAL-40	SRX021235	61.3	61,345,170	33.3
90	DGRP_321	Bloomington-29655	inbred line, mix of	SRX021094	RAL-321	SRR834511	95	95	95	DGRP-F1	33.7	95bp:67314152	DGRI	F1	Blo	29655	RAL-321	SRX021094	61.7	61,687,556	33.5
91	DGRP_443	Bloomington-28199	inbred line, mix of	SRX021260	RAL-443	SRR835031	95	95	95	DGRP-F1	28.8	95bp:57567568	DGRI	F1	Blo	28199	RAL-443	SRX021260	52.5	52,492,412	28.5
92	DGRP_38	Bloomington-28125	inbred line, mix of	SRX025317	RAL-38	SRR834541	95	95	95	DGRP-F1	28.1	95bp:56154204	DGRI	F1	Blo	28125	RAL-38	SRX025317	51.6	51,612,838	28
93	DGRP_320	Bloomington-29654	inbred line, mix of	SRX021063	RAL-320	SRR834510	95	95	95	DGRP-F1	25.9	95bp:51875680	DGRI	F1	Blo	29654	RAL-320	SRX021063	44.5	44,510,214	24.2
94	DGRP_406	Bloomington-29657	inbred line, mix of	SRX021254	RAL-406	SRR835024	95	95	95	DGRP-F1	25.9	95bp:51821248	DGRI	F1	Blo	29657	RAL-406	SRX021254	46.0	46,001,604	25
95	DGRP_392	Bloomington-28194	inbred line, mix of	SRX021157	RAL-392	SRR834520	95	95	95	DGRP-F1	25.6	95bp:51156860	DGRI	F1	Blo	28194	RAL-392	SRX021157	42.8	42,803,392	23.2
96	DGRP_405	Bloomington-29656	inbred line, mix of	SRX021242	RAL-405	SRR835023	95	95	95	DGRP-F1	25.0	95bp:50080536	DGRI	F1	Blo	29656	RAL-405	SRX021242	42.2	42,196,622	22.9
97	DGRP_461	Bloomington-28200	inbred line, mix of	SRX021262	RAL-461	SRR835033	95	95	95	DGRP-F1	24.7	95bp:49324528	DGRI	F1	Blo	28200	RAL-461	SRX021262	40.4	40,407,678	21.9
98	DGRP_177	Bloomington-28150	inbred line, mix of	SRX021026	RAL-177	SRR834547	95	95	95	DGRP-F1	24.6	95bp:49114764	DGRI	F1	Blo	28150	RAL-177	SRX021026	45.3	45,259,994	24.6
99	DGRP_837	Bloomington-28246	inbred line, mix of	SRX021479	RAL-837	SRR933599	95	95	95	DGRP-F1	23.2	95bp:46411538	DGRI	F1	Blo	28246	RAL-837	SRX021479	38.0	38,040,748	20.7
100	DGRP_892	Bloomington-28258	inbred line, mix of	SRX023838	RAL-892	SRR835072	95	95	95	DGRP-F1	22.9	95bp:45702226	DGRI	F1	Blo	28258	RAL-892	SRX023838	37.8	37,797,872	20.5
101	DGRP_439	Bloomington-29658	inbred line, mix of	SRX021244	RAL-439	SRR835028	95	95	95	DGRP-F1	22.4	95bp:44762436	DGRI	F1	Blo	29658	RAL-439	SRX021244	37.5	37,522,394	20.4
102	DGRP_492	Bloomington-28203	inbred line, mix of	SRX021270	RAL-492	SRR835036	95	95	95	DGRP-F1	22.3	95bp:44580310	DGRI	F1	Blo	28203	RAL-492	SRX021270	40.7	40,704,078	22.1
103	DGRP_502	Bloomington-28204	inbred line, mix of	SRX021271	RAL-502	SRR835038	95	95	95	DGRP-F1	22.2	95bp:44336646	DGRI	F1	Blo	28204	RAL-502	SRX021271	40.0	39,968,920	21.7
104	DGRP_370	Bloomington-28182	inbred line, mix of	SRX021104	RAL-370	SRR834539	95	95	95	DGRP-F1	21.9	95bp:43793604	DGRI	F1	Blo	28182	RAL-370	SRX021104	38.4	38,408,446	20.9
105	DGRP_377	Bloomington-28186	inbred line, mix of	SRX023834	RAL-377	SRR834543	95	95	95	DGRP-F1	21.9	95bp:43796182	DGRI	F1	Blo	28186	RAL-377	SRX023834	40.2	40,225,994	21.8
106	DGRP_426	Bloomington-28196	inbred line, mix of	SRX021245	RAL-426	SRR835026	95	95	95	DGRP-F1	21.9	95bp:43746634	DGRI	F1	Blo	28196	RAL-426	SRX021245	38.9	38,928,098	21.1
107	DGRP_887	Bloomington-28279	inbred line, mix of	SRX021527	RAL-887	SRR835069	95	95	95	DGRP-F1	21.8	95bp:43595728	DGRI	F1	Blo	28279	RAL-887	SRX021527	35.9	35,939,248	19.5
108	DGRP_440	Bloomington-28197	inbred line, mix of	SRX021246	RAL-440	SRR835029	95	95	95	DGRP-F1	21.6	95bp:43161850	DGRI	F1	Blo	28197	RAL-440	SRX021246	31.7	31,659,420	17.2
109	DGRP_513	Bloomington-29659	inbred line, mix of	SRX021282	RAL-513	SRR835042	95	95	95	DGRP-F1	21.3	95bp:42640722	DGRI	F1	Blo	29659	RAL-513	SRX021282	36.0	36,025,830	19.6
110	DGRP_508	Bloomington-28205	inbred line, mix of	SRX021272	RAL-508	SRR835040	95	95	95	DGRP-F1	21.2	95bp:42338556	DGRI	F1	Blo	28205	RAL-508	SRX021272	39.1	39,078,690	21.2
111	DGRP_441	Bloomington-28198	inbred line, mix of	SRX023835	RAL-441	SRR835030	95	95	95	DGRP-F1	21.1	95bp:42278010	DGRI	F1	Blo	28198	RAL-441	SRX023835	34.4	34,448,902	18.7
112	DGRP_531	Bloomington-28207	inbred line, mix of	SRX021290	RAL-531	SRR835045	95	95	95	DGRP-F1	20.8	95bp:41560152	DGRI	F1	Blo	28207	RAL-531	SRX021290	32.9	32,916,204	17.9
113	DGRP_383	Bloomington-28190	inbred line, mix of	SRX021113	RAL-383	SRR834554	95	95	95	DGRP-F1	19.9	95bp:39897030	DGRI	F1	Blo	28190	RAL-383	SRX021113	35.1	35,149,446	19.1
114	DGRP_235	Bloomington-28275	inbred line, mix of	SRX021053	RAL-235	SRR834531	95	95	95	DGRP-F1	19.1	95bp:38296004	DGRI	F1	Blo	28275	RAL-235	SRX021053	33.8	33,829,434	18.4
115	DGRP_42	Bloomington-28127	inbred line, mix of	SRX021255	RAL-42	SRR835027	95	95	95	DGRP-F1	18.6	95bp:37186556	DGRI	F1	Blo	28127	RAL-42	SRX021255	37.2	37,186,556	20.2
116	DGRP_21	Bloomington-28122	inbred line, mix of	SRX021040	RAL-21	SRR834526	95	95	95	DGRP-F1	18.5	95bp:37046984	DGRI	F1	Blo	28122	RAL-21	SRX021040	29.2	29,159,002	15.8
117	DGRP_907	Bloomington-28262	inbred line, mix of	SRX021500	RAL-907	SRR835074	95	95	95	DGRP-F1	18.2	95bp:36385056	DGRI	F1	Blo	28262	RAL-907	SRX021500	32.3	32,273,788	17.5
118	DGRP_790	Bloomington-28232	inbred line, mix of	SRX021389	RAL-790	SRR835092	95	95	95	DGRP-F1	17.8	95bp:35620658	DGRI	F1	Blo	28232	RAL-790	SRX021389	31.3	31,287,054	17
119	DGRP_894	Bloomington-28259	inbred line, mix of	SRX021528	RAL-894	SRR835073	95	95	95	DGRP-F1	17.6	95bp:35128536	DGRI	F1	Blo	28259	RAL-894	SRX021528	30.9	30,860,088	16.8
120	DGRP_832	Bloomington-28245	inbred line, mix of	SRX021477	RAL-832	SRR835057	95	95	95	DGRP-F1	16.9	95bp:33770198	DGRI	F1	Blo	28245	RAL-832	SRX021477	26.0	26,008,378	14.1
121	DGRP_908	Bloomington-28263	inbred line, mix of	SRX021501	RAL-908	SRR835075	95	95	95	DGRP-F1	16.8	95bp:39111536	DGRI	F1	Blo	28263	RAL-908	SRX021501	36.7	36,702,788	19.9
122	DGRP_911	Bloomington-28264	inbred line, mix of	SRX021502	RAL-911	SRR835076	95	95	95	DGRP-F1	16.8	95bp:33571736	DGRI	F1	Blo	28264	RAL-911	SRX021502	26.3	26,331,524	14.3
123	DGRP_703	Bloomington-28218	inbred line, mix of	SRX021508	RAL-703	SRR835084	95	95	95	DGRP-F1	16.5	95bp:32953564	DGRI	F1	Blo	28218	RAL-703	SRX021508	26.3	26,272,930	14.3
124	DGRP_385	Bloomington-28191	inbred line, mix of	SRX159098	RAL-385	SRR834518	95	95	95	DGRP-F2	14.2	95bp:28408672	DGRI	F2	Blo	28191	RAL-385	SRX159098	21.0	20,956,926	11.4
125	DGRP_374	Bloomington-28185	inbred line, mix of	SRX023427	RAL-374	SRR835103	95	95	95	DGRP-F1	13.9	95bp:27645755	DGRI	F1	Blo	28185	RAL-374	SRX023427	24.7	24,704,590	13.4

Table S1D. Drosophila Genome Nexus Strains in the TIDAL-Fly v1 database

Genomes previously examined in Lack et al (Pool lab), Genetics 2015

PHASE 1: First Set of Libraries Analyzed for the paper. The remaining libraries in Phase 2 below are also listed in the TIDAL-Fly database

	Stock ID*		Stock Location	Genome Type	SRA Accession	Library ID*	SRA Accession	Min Read Length	Reads (M)	Read Length 1	Read Length 2	Data Group	Focal Genome Repre	Coverage**	Mean Depth**
1	CK1		Langley lab	haploid_embryo	SRX058145	CK1_1-HE	SRR189038	75	35.0	76	76	DPGP2	X,2L,2R,3L,3R	110,870,215	39.4
2	CK2		Langley lab	haploid_embryo	SRX058146	CK2_1-HE	SRR189040	145	20.6	146	146	DPGP2	X,2L,2R,3L,3R	111,567,582	44.7
3	CO13N		Pool Lab	haploid_embryo	SRX058151	CO13N_1-HE	SRR189045	145	18.1	146	146	DPGP2	X,2L,2R,3L,3R	111,264,202	39.8
4	CO15N		Pool Lab	haploid_embryo	SRX058153	CO15N_1-HE	SRR189047	75	38.2	76	76	DPGP2	X,2L,2R,3L,3R	111,058,052	44.2
5	CO4N		Pool Lab	haploid_embryo	SRX058156	CO4N_1-HE	SRR189050	75	32.8	76	76	DPGP2	X,2L,2R,3L,3R	111,025,671	37.7
6	EA119		Pool Lab	haploid_embryo	SRX791698	EA119_1-HE	SRR1686794	100	16.6	100	100	AGES	X,2L,2R,3L,3R	110,802,511	23.9
7	EA49		Pool Lab	haploid_embryo	SRX791713	EA49_1-HE	SRR1686797	100	13.0	100	100	AGES	X,2L,2R,3L,3R	110,446,851	18.2
8	EA87		Pool Lab	haploid_embryo	SRX791850	EA87_1-HE	SRR1686966	100	27.6	100	100	AGES	X,2L,2R,3L,3R	111,259,464	38.9
9	EB148		Langley lab	haploid_embryo	SRX791858	EB148_1-HE	SRR1686971	100	19.1	100	100	AGES	X,2L,2R,3L,3R	110,799,356	27.4
10	EB18		Langley lab	haploid_embryo	SRX792309	EB18_1-HE	SRR1687447	100	16.5	100	100	AGES	X,2L,2R,3L,3R	110,159,072	22.7
11	EB25		Langley lab	haploid_embryo	SRX792329	EB25_1-HE	SRR1687462	100	15.6	100	100	AGES	X,2L,2R,3L,3R	103,098,821	19.4
12	EF39		Pool lab	haploid_embryo	SRX792510	EF39_1-HE	SRR1687668	100	17.7	100	100	AGES	X,2L,2R,3L,3R	110,937,745	24.9
13	EF65		Pool lab	haploid_embryo	SRX792606	EF65_1-HE	SRR1687770	100	12.2	100	100	AGES	X,2L,2R,3L,3R	107,539,051	17.1
14	EF78		Pool lab	haploid_embryo	SRX792639	EF78_1-HE	SRR1687793	100	16.8	100	100	AGES	X,2L,2R,3L,3R	110,871,321	23.8
15	EG40N		Pool lab	inbred_line	SRX792675	EG40N	SRR1687832	100	10.8	100	100	AGES	X,2R	25,659,426	14.3
16	EG69N		Pool lab	inbred_line	SRX793022	EG69N	SRR1688188	100	10.2	100	100	AGES	X,2L,2R,3R	73,335,770	13.6
17	EG73N		Pool lab	inbred_line	SRX793046	EG73N	SRR1688222	100	10.3	100	100	AGES	X,2L,2R,3L,3R	105,554,844	13.8
18	FR180		Pool Lab	haploid_embryo	SRX058184	FR180_2-HE	SRR189090	145	19.1	146	146	DPGP2	X,2L,2R,3L,3R	112,084,373	42.3
19	FR207		Pool Lab	haploid_embryo	SRX058185	FR207_2-HE	SRR189091	145	18.1	146	146	DPGP2	X,2L,2R,3L,3R	112,168,144	38.9
20	FR229		Pool Lab	haploid_embryo	SRX058187	FR229_2-HE	SRR189093	145	19.1	146	146	DPGP2	X,2L,2R,3L,3R	112,159,960	40.6
21	GA129		Pool lab	haploid_embryo	SRX058193	GA129_1-HE	SRR189101	75	41.4	76	76	DPGP2	X,2L,2R,3L,3R	111,099,834	47.9
22	GA130		Pool lab	haploid_embryo	SRX058194	GA130_1-HE	SRR189102	75	43.4	76	76	DPGP2	X,2L,2R,3L,3R	111,771,726	47.6
23	GA141		Pool lab	haploid_embryo	SRX058196	GA141_1-HE	SRR189105	75	40.7	76	76	DPGP2	X,2L,2R,3L,3R	111,146,499	45.1
24	GU10		Langley lab	haploid_embryo	SRX058205	GU10_1-HE	SRR189114	75	33.2	76	76	DPGP2	X,2L,2R,3L,3R	110,887,105	38.9
25	GU2		Langley lab	haploid_embryo	SRX058207	GU2_1-HE	SRR189117	75	33.0	76	76	DPGP2	X,2L,2R,3L,3R	110,886,266	38.4
26	GU6		Langley lab	haploid_embryo	SRX058209	GU6_1-HE	SRR189120	75	33.0	76	76	DPGP2	X,2L,2R,3L,3R	110,933,340	38.1
27	KN133N		Langley lab	haploid_embryo	SRX058256	KN133N_1-HE	SRR189242	75	30.1	76	76	DPGP2	X,2L,2R,3L,3R	111,123,747	35.0
28	KN20N		Langley lab	haploid_embryo	SRX058253	KN20N_1-HE	SRR189239	75	32.0	76	76	DPGP2	X,2L,2R,3L,3R	110,974,588	37.6
29	KN35		Langley lab	haploid_embryo	SRX058258	KN35_1-HE	SRR189245	75	30.2	76	76	DPGP2	X,2L,2R,3L,3R	111,066,184	34.3
30	KR39		Langley lab	haploid_embryo	SRX058267	KR39_1-HE	SRR189254	75	30.2	76	76	DPGP2	X,2L,2R,3L,3R	111,101,981	34.7
31	KR42		Langley lab	haploid_embryo	SRX058268	KR42_1-HE	SRR189255	75	28.5	76	76	DPGP2	X,2L,2R,3L,3R	110,823,311	32.9
32	KR4N		Langley lab	haploid_embryo	SRX058269	KR4N_1-HE	SRR189256	75	29.5	76	76	DPGP2	X,2L,2R,3L,3R	111,045,036	29.6
33	KT1		Langley lab	haploid_embryo	SRX058273	KT1_1-HE	SRR189260	75	25.5	76	76	DPGP2	X,2L,2R,3L,3R	111,229,668	37.6
34	KT6		Langley lab	haploid_embryo	SRX058274	KT6_1-HE	SRR189262	75	27.6	76	76	DPGP2	X,2L,2R,3L,3R	111,074,001	29.6
35	NG10N		Langley lab	haploid_embryo	SRX058275	NG10N_1-HE	SRR189263	75	33.5	76	76	DPGP2	X,2L,2R,3L,3R	111,001,728	38.9
36	NG3N		Pool lab	haploid_embryo	SRX058378	NG3N_1-HE	SRR189414	75	32.3	76	76	DPGP2	X,2L,2R,3L,3R	111,022,313	36.4
37	NG6N		Pool lab	haploid_embryo	SRX058278	NG6N_1-HE	SRR189266	75	33.9	76	76	DPGP2	X,2L,2R,3L,3R	111,483,632	38.4
38	RC1		Langley lab	haploid_embryo	SRX058281	RC1_1-HE	SRR189269	75	26.2	76	76	DPGP2	X,2L,2R,3L,3R	110,659,330	30.3
39	RC5		Langley lab	haploid_embryo	SRX058282	RC5_1-HE	SRR189270	75	29.6	76	76	DPGP2	X,2L,2R,3L,3R	110,723,230	24.9
40	RG33		Pool lab	haploid_embryo	SRX058353	RG33_1-HE	SRR189389	145	37.0	146	146	DPGP2	X,2L,2R,3L,3R	111,689,097	69.8
41	RG4N		Pool lab	haploid_embryo	SRX058362	RG4N_1-HE	SRR306629	145	24.9	146	146	DPGP2	X,2L,2R,3L,3R	111,396,200	54.2
42	RG6N		Langley lab	haploid_embryo	SRX058368	RG6N_1-HE	SRR306624	145	22.0	146	146	DPGP2	X,2L,2R,3L,3R	111,216,270	47.7
43	SB10		Pool lab	haploid_embryo	SRX799453	SB10_1-HE	SRR1696817	100	23.7	100	100	AGES	X,2L,2R,3L,3R	111,238,977	34.2

44	SB16		Pool lab	haploid_embryo	SRX799458	SB16_1-HE	SRR1696818	100	24.1	100	100	AGES	X,2L,2R,3L,3R	111,438,387	32.4
45	SB31		Pool lab	haploid_embryo	SRX799471	SB31_1-HE	SRR1696822	100	21.4	100	100	AGES	X,2L,2R,3L,3R	110,110,096	30.2
46	SF332		Langley lab	haploid_embryo	SRX799661	SF332_1-HE	SRR1696987	100	13.7	100	100	AGES	X,2L,2R,3L,3R	110,582,475	19.9
47	SF428		Langley lab	haploid_embryo	SRX799663	SF428_1-HE	SRR1696988	100	12.6	100	100	AGES	X,2L,2R,3L,3R	110,495,354	16.5
48	SF447		Pool lab	haploid_embryo	SRX799664	SF447_1-HE	SRR1696989	100	15.3	100	100	AGES	X,2L,2R,3L,3R	110,719,070	20.7
49	SP188		Langley lab	haploid_embryo	SRX058287	SP188_1-HE	SRR306632	145	18.4	146	146	DPGP2	X,2L,2R,3L,3R	111,309,908	38.6
50	SP221		Pool lab	haploid_embryo	SRX058288	SP221_1-HE	SRR189277	145	18.9	146	146	DPGP2	X,2L,2R,3L,3R	111,199,509	40.7
51	SP80		Langley lab	haploid_embryo	SRX058292	SP80_1-HE	RR189281	145	18.3	146	146	DPGP2	X,2L,2R,3L,3R	111,128,089	41.6
52	TZ10		Langley lab	haploid_embryo	SRX058283	TZ10_1-HE	SRR189272	75	23.4	76	76	DPGP2	X,2L,2R,3L,3R	110,737,417	26.9
53	TZ14		Langley lab	haploid_embryo	SRX058284	TZ14_1-HE	SRR189273	75	31.7	76	76	DPGP2	X,2L,2R,3L,3R	111,045,117	35.3
54	TZ8		Langley lab	haploid_embryo	SRX058285	TZ8_1-HE	SRR189274	75	31.2	76	76	DPGP2	X,2L,2R,3L,3R	110,920,135	35.9
55	UG19		Pool lab	haploid_embryo	SRX058376	UG19_1-HE	SRR189412	75	33.9	76	76	DPGP2	X,2L,2R,3L,3R	110,998,446	37.2
56	UG28N		Pool lab	haploid_embryo	SRX058277	UG28N_1-HE	SRR189265	75	33.1	76	76	DPGP2	X,2L,2R,3L,3R	110,922,524	38.8
57	UG5N		Pool lab	haploid_embryo	SRX058379	UG5N_1-HE	SRR189415	75	32.1	76	76	DPGP2	X,2L,2R,3L,3R	111,110,172	34.2
58	UK120		Langley lab	haploid_embryo	SRX799667	UK120_1-HE	SRR1696992	100	15.6	100	100	AGES	X,2L,2R,3L,3R	108,541,523	23.2
59	UK2		Langley lab	haploid_embryo	SRX799668	UK2_1-HE	SRR1696993	100	18.3	100	100	AGES	X,2L,2R,3L,3R	111,035,774	25.2
60	UK57		Langley lab	haploid_embryo	SRX799669	UK57_1-HE	SRR1696994	100	20.7	100	100	AGES	X,2L,2R,3L,3R	110,904,117	29.9
61	ZI191		Langley/Pool Lab	haploid_embryo	SRR202128	ZI191-HE	SRR202128	145	23.3	146	146	DPGP3	X,2L,2R,3L,3R	110,845,648	49.2
62	ZI216N		Langley/Pool Lab	haploid_embryo	SRR203328	ZI216N-HE	SRR203328	145	20.4	146	146	DPGP3	X,2L,2R,3L,3R	111,176,278	44.2
63	ZI220		Langley/Pool Lab	haploid_embryo	SRR203067	ZI220-HE	SRR203067	145	23.2	146	146	DPGP3	X,2L,2R,3L,3R	111,180,528	51.3
64	ZI227		Langley/Pool Lab	haploid_embryo	SRR202126	ZI227-HE	SRR202126	145	25.7	146	146	DPGP3	X,2L,2R,3L,3R	111,269,290	54.7
65	ZI254N		Langley/Pool Lab	haploid_embryo	SRR203335	ZI254N-HE	SRR203335	145	18.1	146	146	DPGP3	X,2L,2R,3L,3R	111,099,774	41.2
66	ZI273N		Langley/Pool Lab	haploid_embryo	SRR210786	ZI273N-HE	SRR210786	145	21.1	146	146	DPGP3	X,2L,2R,3L,3R	111,341,925	43.2
67	ZS11		Langley lab	haploid_embryo	SRX058372	ZS11_1-HE	SRR189408	75	32.4	76	76	DPGP2	X,2L,2R,3L,3R	110,736,467	37.7
68	ZS37		Langley lab	haploid_embryo	SRX058373	ZS37_1-HE	SRR189409	75	36.0	76	76	DPGP2	X,2L,2R,3L,3R	110,877,107	41.2
69	ZS5		Langley lab	haploid_embryo	SRX058374	ZS5_1-HE	SRR900425	75	33.4	76	76	DPGP2	X,2L,2R,3L,3R	110,504,765	38.2
70	ZS56		Langley lab	haploid_embryo	SRX058375	ZS56_1-HE	SRR189411	75	30.7	76	76	DPGP2	X,2L,2R,3L,3R	110,737,628	36.0

Table S1E. Key for Two-letter code for the demography of the Drosophila Genome Nexus Strains in the TIDAL-Fly v1 database

									Number of genomes for each euchromatic chromosome			
	**Populatio**	**Country**	**Locality**	**Date**	**Collector(s)**	**Latitude (**	**Longitude**	**Elevation**	**X**	**2**	**3**	**Comments**
1	**CK**	Congo	Kisangani	8/2010	J. Kennis	0.51	25.19	400	2	2	2	
2	**CO**	Cameroon	Oku	4/2004	J. Pool	6.25	10.43	2169	10	10	10	3 additional chromosome 3 extractions exist, but derive from str
3	**EA**	Ethiopia	Gambella	12/2011	R. Corbett-Detig	8.25	34.59	525	4	4	4	
4	**EB**	Ethiopia	Bonga	12/2011	R. Corbett-Detig	7.26	36.25	1725	5	5	5	
5	**ED**	Ethiopia	Dodola	12/2008	J. Pool	6.98	39.18	2492	6	7	6	
6	**EF**	Ethiopia	Fiche	12/2011	R. Corbett-Detig	9.81	38.63	3070	5	5	5	
7	**EG**	Egypt	Cairo	1/2011	J. Atallah	30.10	31.32	25	3	2	2	contains heterozygous intervals (masked in consensus sequenc
8	**EM**	Ethiopia	Masha	12/2011	R. Corbett-Detig	7.74	35.48	2260	3	3	3	
9	**ER**	Ethiopia	Debre Birha	12/2011	R. Corbett-Detig	9.68	39.53	2840	5	5	5	
10	**EZ**	Ethiopia	Ziway	12/2008	J. Pool	7.93	38.72	1642	5	4	5	
11	**FR**	France	Lyon	7/2010	J. Pool	45.77	4.86	175	9	9	9	
12	**GA**	Gabon	Franceville	3/2002	B. Ballard & S. Charlat	-1.65	13.60	332	10	9	10	GA191 is missing chromosome 2 data due to heterozygosity
13	**GU**	Guinea	Dondé	6/2005	B. B. Sow	10.70	-12.25	801	5	5	7	
14	**KM**	Kenya	Malindi	7/2002	B. Ballard	-1.43	40.03	78	4	3	1	contains heterozygous intervals (masked in consensus sequenc
15	**KN**	Kenya	Nyahururu	1/2009	J. Pool	0.04	36.36	2303	6	5	6	
16	**KO**	Kenya	Molo	1/2009	J. Pool	-0.25	35.73	2506	4	0	4	
17	**KR**	Kenya	Marigat	1/2009	J. Pool	0.47	35.98	1062	6	4	6	
18	**KT**	Kenya	Thika	1/2009	J. Pool	-1.04	37.08	1531	2	2	2	
19	**NG**	Nigeria	Maiduguri	9/2004	D. Gwary & B. Sastawa	11.85	13.16	295	6	6	6	
20	**RAL**	United State	Raleigh NC	2003	T. Mackay	35.76	-78.66	91	205	205	205	(sample sizes before heterozygosity and IBD masking)
21	**RC**	Rwanda	Cyangugu	12/2008	J. Pool	-2.29	28.55	1602	2	2	2	
22	**RG**	Rwanda	Gikongoro	12/2008	J. Pool	-2.49	28.92	1927	27	27	27	
23	**SB**	South Africa	Barkly East	12/2011	J. Pool	-30.97	27.59	1800	5	5	5	
24	**SD**	South Africa	Dullstroom	12/2011	J. Pool	-25.42	30.10	2000	5	4	5	SD82 is missing chromosome 2 data due to heterozygosity
25	**SE**	South Africa	Port Edward	12/2011	J. Pool	-31.06	30.22	50	3	3	3	
26	**SF**	South Africa	Fouriesburg	12/2011	J. Pool	-28.60	28.05	1800	5	4	5	SF7 is missing chromosome 2 data due to heterozygosity
27	**SP**	South Africa	Phalaborwa	7/2010	R. Corbett-Detig	-23.94	31.14	350	7	7	7	
28	**TZ**	Tanzania	Uyole	12/2009	L. Nsemwa	-8.89	33.44	1800	3	3	3	
29	**UG**	Uganda	Namulonge	4/2005	J. Ogwang	0.53	32.60	1134	4	4	6	
30	**UK**	Uganda	Kisoro	1/2012	R. Corbett-Detig	-1.28	29.69	1925	5	4	5	UK120 is missing chromosome 2 data due to heterozygosity
31	**UM**	Uganda	Masindi	7/2010	J. Pool	1.68	31.72	1170	3	3	3	
32	**ZI**	Zambia	Siavonga	7/2010	R. Corbett-Detig	-16.54	28.72	530	196	197	197	ZI382 is missing chromosome X due to heterozygosity
33	**ZK**	Zimbabwe	Lake Kariba	5/1994	T. Mutangadura	-16.52	28.80	619	3	0	2	contains heterozygous intervals (masked in consensus sequenc
34	**ZL**	Zambia	Livingstone	7/2010	R. Corbett-Detig	-17.86	25.86	900	1	1	1	
35	**ZO**	Zambia	Solwezi	7/2010	R. Corbett-Detig	-12.18	26.40	1380	2	2	2	
36	**ZS**	Zimbabwe	Sengwa	9/1990	R. Ramey	-18.16	28.22	865	4	4	4	

Table S1F.Pools of flies analyzed in the TIDAL-Fly v1 database

	Fly Pool ID	Fly Pool Location	Genome Type	SRA Accession	Library ID*	SRA Accession	Min Read Length	Reads (M)	Read Length 1	Read Length 2	Reference
1	AU-CI	Australia, Cairns	Pool	SRR1177951	pool-AU-CI	SRR1177951	75	26M	75	0	Reinhardt et al.,2014
2	AU-CR	Australia, Cardwell	Pool	SRR1177952	pool-AU-CR	SRR1177952	75	36M	75	0	Reinhardt et al.,2014
3	AU-MO	Australia, Miller's Orchard	Pool	SRR1177953	pool-AU-MO	SRR1177953	75	24M	75	0	Reinhardt et al.,2014
4	AU-SO	Australia, Sorell	Pool	SRR1177955	pool-AU-SO	SRR1177955	75	38M	75	0	Reinhardt et al.,2014
5	EU-PO1	Europe, Portugal	Pool	SRR188217	pool-EU-PO1	SRR188217	75	19.5M	75	75	Kofler et al., 2012
6	EU-PO2	Europe, Portugal	Pool	SRR189066	pool-EU-PO2	SRR189066	73	60.9M	74	74	Kofler et al., 2012
7	NA-SC	North America, Eutawville, SC	50-100 pooled individuals	SRX661835	pool-NA-SC	SRR1525696	100	82M	100	100	Bergland et al.,2014
8	NA-GA	North America, Hahira, GA	50-100 pooled individuals	SRX661834	pool-NA-GA	SRR1525695	100	97M	100	100	Bergland et al.,2014
9	NA-NC	North America, 90 of the DGRP fly stra	Pool	SRX661836	pool-NA-NC	SRR1525697	90	42M	90	90	Bergland et al.,2014
10	NA-PA1	North America, Linvilla, PA, spring 200	50-100 pooled individuals	SRX661837	pool-NA-PA1	SRR1525768	100	181M,	100	100	Bergland et al.,2014
11	NA-PA2	North America,Linvilla, PA, fall 2009	50-100 pooled individuals	SRX661838	pool-NA-PA2	SRR1525769	100	73.7M	100	100	Bergland et al.,2014
12	NA-PA3	North America,Linvilla, PA, spring 201(	50-100 pooled individuals	SRX661839	pool-NA-PA3	SRR1525770	100	35M	100	100	Bergland et al.,2014
13	NA-PA4	North America,Linvilla, PA, fall 2010	50-100 pooled individuals	SRX661840	pool-NA-PA4	SRR1525771	100	70M	100	100	Bergland et al.,2014
14	NA-PA5	North America,Linvilla, PA, spring 201	50-100 pooled individuals	SRX661841	pool-NA-PA5	SRR1525772	90	78.1M	90	90	Bergland et al.,2014
15	NA-PA6	North America,Linvilla, PA, fall 2011 (p	50-100 pooled individuals	SRX661842	pool-NA-PA6	SRR1525773	100	73.2M	100	100	Bergland et al.,2014
16	NA-PA7	North America,Linvilla, PA, fall 2011 (p	50-100 pooled individuals	SRX661843	pool-NA-PA7	SRR1525774	90	82.1M	90	90	Bergland et al.,2014
17	NA-ME1	North America,Bowdoinham, ME	50-100 pooled individuals	SRX661844	pool-NA-ME1	SRR1525698,	100	95M,	100	100	Bergland et al.,2014
18	NA-ME2	North America,Bowdoinham, ME	50-100 pooled individuals	SRX661845	pool-NA-ME2	SRR1525699, SRR2006283	100	32M, 32M	100	100	Bergland et al.,2014
19	NA-FL1	North America,Homestead, FL	50-100 pooled individuals	SRX661832	pool-NA-FL1	SRR1525685	100	64M,	100	100	Bergland et al.,2014
20	NA-FL2	North America,Homestead, FL	50-100 pooled individuals	SRX661833	pool-NA-FL2	SRR1525694	100	41.6M	100	100	Bergland et al.,2014
21	EU-AU-LF1	Austria, lightest females	Pool, 100 females	ERX149369	pool-EU-AU-LF1	ERR173225	100	86.1	100	100	Bastide et al., 2013
22	EU-AU-LF2	Austria, lightest females	Pool, 100 females	ERX149370	pool-EU-AU-LF2	ERR173226	100	95.0	100	100	Bastide et al., 2013
23	EU-AU-DF1	Austria, darkest females	Pool, 100 females	ERX149371	pool-EU-AU-DF1	ERR173227	100	34.5	100	100	Bastide et al., 2013
24	EU-AU-DF2	Austria, darkest females	Pool, 100 females	ERX149372	pool-EU-AU-DF2	ERR173228	150	39.7	150	85	Bastide et al., 2013
25	EU-AU-DF3	Austria, darkest females	Pool, 100 females	ERX149373	pool-EU-AU-DF3	ERR173229	100	95.0	100	100	Bastide et al., 2013
26	EU-AU1	Austria/Vienna reference	Pool, 100-150 females	ERX149374	pool-EU-AU1	ERR173230	100	41.0	100	100	Bastide et al., 2013
27	EU-AU2	Austria/Vienna reference	Pool, 100-150 females	ERX149375	pool-EU-AU2	ERR173231	150	35.3	150	80	Bastide et al., 2013
28	EU-AU3	Austria/Vienna reference	Pool, 100-150 females	ERX149376	pool-EU-AU3	ERR173232	100	38.9M	100	100	Bastide et al., 2013
29	EU-AU4	Austria/Vienna reference	Pool, 100-150 females	ERX149377	pool-EU-AU4	ERR173233	150	40.7	150	80	Bastide et al., 2013
30	EU-AU5	Austria/Vienna reference	Pool, 100-150 females	ERX149378	pool-EU-AU5	ERR173234	100	41.7	100	100	Bastide et al., 2013
31	EU-AU6	Austria/Vienna reference	Pool, 100-150 females	ERX149379	pool-EU-AU6	ERR173235	100	41.9	100	100	Bastide et al., 2013
32	EU-AU7	Austria/Vienna reference	Pool, 100-150 females	ERX149380	pool-EU-AU7	ERR173236	100	40.8	100	100	Bastide et al., 2013
33	EU-AU8	Austria/Vienna reference	Pool, 100-150 females	ERX149381	pool-EU-AU8	ERR173237	100	40.2	100	100	Bastide et al., 2013
34	EU-AU9	Austria/Vienna reference	Pool, 100-150 females	ERX149382	pool-EU-AU9	ERR173238	100	40.6M	100	100	Bastide et al., 2013
35	EU-AU10	Austria/Vienna reference	Pool, 100-150 females	ERX149383	pool-EU-AU10	ERR173239	150	40.9M	150	80	Bastide et al., 2013
36	EU-AU11	Austria/Vienna reference	Pool, 100-150 females	ERX149384	pool-EU-AU11	ERR173240	100	40	100	100	Bastide et al., 2013
37	EU-AU12	Austria/Vienna reference	Pool, 100-150 females	ERX149385	pool-EU-AU12	ERR173241	150	40.1	150	80	Bastide et al., 2013
38	EU-IT-LF1	Italy, lightest females	Pool, 100 females	ERX149386	pool-EU-IT-LF1	ERR173242	90	14.6	90	90	Bastide et al., 2013
39	EU-IT-LF2	Italy, lightest females	Pool, 100 females	ERX149387	pool-EU-IT-LF2	ERR173243	90	15.6	90	90	Bastide et al., 2013
40	EU-IT-LF3	Italy, lightest females	Pool, 100 females	ERX149388	pool-EU-IT-LF3	ERR173244	90	15.6	90	90	Bastide et al., 2013
41	EU-IT-LF4	Italy, lightest females	Pool, 100 females	ERX149389	pool-EU-IT-LF4	ERR173245	100	138.8	100	100	Bastide et al., 2013
42	EU-IT-LF5	Italy, lightest females	Pool, 100 females	ERX149390	pool-EU-IT-LF5	ERR173246	100	131.7	100	100	Bastide et al., 2013
43	EU-IT-DF1	Italy, darkest females	Pool, 100 females	ERX149391	pool-EU-IT-DF1	ERR173247	92	11.1	92	92	Bastide et al., 2013
44	EU-IT-DF2	Italy, darkest females	Pool, 100 females	ERX149392	pool-EU-IT-DF2	ERR173248	92	10.3	92	92	Bastide et al., 2013
45	EU-IT-DF3	Italy, darkest females	Pool, 100 females	ERX149393	pool-EU-IT-DF3	ERR173249	92	9.5	92	92	Bastide et al., 2013
46	EU-IT-DF4	Italy, darkest females	Pool, 100 females	ERX149394	pool-EU-IT-DF4	ERR173250	100	138.4	100	100	Bastide et al., 2013
47	EU-IT-DF5	Italy, darkest females	Pool, 100 females	ERX149395	pool-EU-IT-DF5	ERR173251	100	138.4	100	100	Bastide et al., 2013
48	EU-IT1	Italy, reference	Pool, 100-150 females	ERX149396	pool-EU-IT1	ERR173252	94	21.6	94	94	Bastide et al., 2013
49	EU-IT2	Italy, reference	Pool, 100-150 females	ERX149397	pool-EU-IT2	ERR173253	94	17.4	94	94	Bastide et al., 2013
50	EU-IT3	Italy, reference	Pool, 100-150 females	ERX149398	pool-EU-IT3	ERR173254	94	15.5	94	94	Bastide et al., 2013
51	EU-IT4	Italy, reference	Pool, 100-150 females	ERX149399	pool-EU-IT4	ERR173255	150	132M	150	150	Bastide et al., 2013
52	EU-IT5	Italy, reference	Pool, 100-150 females	ERX149400	pool-EU-IT5	ERR173256	150	153M	150	150	Bastide et al., 2013

Table S2 Primer Lists

Oligos for gDNA libraries construction	PE_Tdot_common_C* linker	AATGATACGGCGACCACCGAGATCTACACTCTTTCCCTACACGACGCTCTTCCGATC*T
	Barcoded linker PE_tdot_CAGCACTA	/5Phos/GATCGGAAGAGCGGTTCAGCAGGAATGCCGAGACCGcagcactaATCTCGTATGCCGTCTTCTGCTTG
	Barcode linker PE_tdot_AGAGATGC	/5Phos/GATCGGAAGAGCGGTTCAGCAGGAATGCCGAGACCGagagatgcATCTCGTATGCCGTCTTCTGCTTG
	Barcode linker PE_tdot_TCACGTGT	/5Phos/GATCGGAAGAGCGGTTCAGCAGGAATGCCGAGACCGtcacgtgtATCTCGTATGCCGTCTTCTGCTTG
	PE-POSTPCR_1	AATGATACGGCGACCACCGAGA
	PE-POSTPCR_2	CAAGCAGAAGACGGCATACGAG

Table S2 Primer Lists

PRIMER SEQUENCE FOR S2c1 INSERTS

Sr. no.	SV#	Chr	Chr_coord_	Chr_coord_	TE	Forward Prmer Sequence	Forward primer Length	Reverse Primer Sequence	Reverse Primer length	Length w/c	Length with TE
1	14	chr2L	312233	312444	Stalker2	ACTGGCAAGACCTCGATTGTC	21	CGGATTGTCCGACGTATTAGA	21	411	8661
2	5	chr2L	87055	87258	1731	GGTGCCGCACAATTCGCAGGC	21	GCTCCGGTCAGAAGATTTTAC	22	404	5053
3	621	chr2R	544728	544940	HMS-Beagle	ATGGGGAACACAGTCAAAATG	21	CATATTATTCAAAGATTGGAT	21	353	6882
4	630	chr2R	2675584	2675743	opus	GAAGAATGAGACGGGCTACCG	21	CTGTAATTGTGTACACAGTCA	21	332	8891
5	1123	chr3L	516184	516383	17.6	AGTCCTCCCAACCGAGATGG	20	GATTGAATTTCAGTTGGCCGG	21	349	7788
6	1131	chr3L	552782	552981	Juan	TTTATAATAATGTTGCTGGAC	21	GGCATTTCTATAGCCCAACCC	21	344	4580
7	1743	chr3R	6430955	6431163	3S18	ATTGAGGCTTTTATTAATCAG	21	GTTCCTATATGCTTGTGATGA	21	293	6126
8	1762	chr3R	7492347	7492543	1360	AATACAAGATATTGATACATC	21	CGAAAGTTCATACAAATGTATA	22	374	4480
9	2406	chrX	141518	141720	blood	TTAATGAAGCCGTTGGGCTAGG	22	CTGTGGTGAATTTGCAGCCTT	21	379	7790
10	2413	chrX	325934	326139	1731	TTTTACACTAAGAGTACCGGC	21	CGACCTAATAGTGCGGAAGCT	21	349	4998
11	2427	chrX	1082310	1082488	297	AGATAAGACGCCTATTAAGTG	21	CATTGTATGTTGCATCTGTAG	21	373	7368
12	32	chr2L	522270	522480	297	ATTTCGAATACGTATCGAATG	21	CATTTCCCTGGCAACTTTGCA	21	386	7381
**13**	**2165**	**chr3R**	**2.3E+07**	**2.3E+07**	**roo**	**ATCGCTTTTCCAATAAATTGCAT**	**23**	**CAGAGACAGCGATTTCAAAT**	**21**	**367**	**9459**
**14**	**2531**	**chrX**	**6005755**	**6005959**	**roo**	**ACCTTCACGCAGTCCAGCTTCG**	**22**	**GCACCTGGCAGGGGATTTTAATT**	**23**	**381**	**9473**
**15**	**1162**	**chr3L**	**957937**	**958157**	**roo**	**TATACAACCACGAGTTCTTGGAAG**	**24**	**ACAACATAAATAATTGCAGAGGC**	**23**	**296**	**9388**

PRIMER SEQUENCE FOR S2c1 DEPLETION

Sr. No.	Chr_cc	Chr_3p	Chr_coord_	Chr_coord_	repName	Forward Primer Sequence	Forward Primer Length	Reverse Primer Sequence	Reverse PrimerLength	Length witl	Length withTE depletic
1		chr2L	7350447	7350536	roo	CTCGCGGTGAGTCAACCCCAG	21	GAGAATTTCGGTGGTTACGCCC	22	7480	5757
2		chrX	6571315	6571399	Tirant	CCATATTTAGACAATTATTC	20	CAGTCCCACAAGTGCGAGCGG	23	8802	1240
3		chr2R	21753581	21753663	roo	GAACACTTTAAGCCCAGTTTG	21	GAACTTCAACAATATCCGAT	20	833	405
4		chr3L	3069878	3069958	Tirant	GACTCCTATGACGCAATACAG	21	CATTCTGTATACAAACAAATC	22	8912	1350
5		chr3L	7889043	7889129	Stalker4	TATGCCTATAATCAATAAATC	21	CTTCATGTTGCTACATAAATCAT	23	2873	1107
6		chr3L	7987706	7987783	NOF	TCCCGAATAGCACCGAATTTC	21	CAATTAATGTGTGAATCTCAAG	22	2650	620
7		chr3L	15426613	15426700	opus	TTTTGGTCGAGTTCTGTTCTG	21	GAAGTCTTTATAAGTCAAGAA	21	3160	2642
8		chr3R	24072571	24072628	jockey	CATTTCTTTGCGCCTGCCATG	21	GACAGCACTTGGATGATCCA	20	341	150
9		chrX	3842569	3842647	pogo	GATCCTTGTCCCGGATAAGTC	21	GATTTTCCCGTTCTGGAGTAA	21	541	355
10		chr3R	24931935	24932010	Doc	TGCAGAATTGTACAAGAGATG	21	CAGATATGTACAATTCCGGACA	22	5120	395

Table S2 Primer Lists

TIDAL_only_TEIns_sites

Site Number	Chr	Chr_coord _5p	Chr_coord _3p	TE	Coverage _Ratio	RAL-765_Inserts_Annotated.Insert_Code	Space	FW primer name	FW primer sequence	Fw Length	Tm	RV primer name	RV primer sequence	Rv Length	Tm	Predicted Amplicon Size
1	chr2L	8133670	8133752	HMS-Beagl	2.4	chr2L_17_8130001	82	TeIns_TID_1_Fw	GATCAAGTCGAACGAACAATT	21	56.8	TeIns_TID_1_Rv	CGCATGCATATAGGCGGATGC	21	67.7	275
2	chr2L	19834994	19835061	hobo	4.7	chr2L_20_19830001	67	TeIns_TID_2_Fw	TAAATATTTATCATTGTGAGCTG	23	51.9	TeIns_TID_2_Rv	AGTTTTATGGCATTTATGTCG	21	54.6	354
3	chr2L	12227998	12228146	BS	5.3	chr2L_10_12225001	148	TeIns_TID_3_Fw	CCAGCTGGCACAAATGAATG	20	62.6	TeIns_TID_3_Rv	CGTTTTAAGCCCACATTGCTG	21	62.7	274
4	chr2R	12581030	12581105	pogo	5	chr2R_14_12580001	75	TeIns_TID_4_Fw	ATTGAAAGAAAAATAGTCATGC	22	53.1	TeIns_TID_4_Rv	CCACTAAGCGATGACGAACGCG	22	69.1	333
5	chr2R	7621371	7621430	mdg1	4	chr2R_10_7620001	59	TeIns_TID_5_Fw	ATGTCAAGAATCTGAAGTTACG	22	53.8	TeIns_TID_5_Rv	CGTTTGTTTATGGTCCAAATGC	22	61.4	328
6	chr3L	4481564	4481640	roo	4.1	chr3L_14_4480001	76	TeIns_TID_6_Fw	GAGTGCAGACCCTACTATTAG	21	51	TeIns_TID_6_Rv	CACGTTCTTATGTGTATAATTA	22	47.8	337
7	chr3L	18394377	18394445	1360	4.2	chr3L_11_18390001	68	TeIns_TID_7_Fw	TCAAACTATGTGTTTTAAGAGTAT	24	50	TeIns_TID_7_Rv	CAGGGTGGATTCGCTATCAGG	21	64	360
8	chr3R	4885334	4885399	1360	1.7	chr3R_13_4885001	65	TeIns_TID_8_Fw	ATTTATGCCAGTCATGGGCC	20	62	TeIns_TID_8_Rv	CGAGCAAGAATGTACCTCAAGT	22	58.5	354
9	chr3R	20125023	20125087	jockey	2.4	chr3R_12_20125001	64	TeIns_TID_9_Fw	AATTGACTTGCCACGATGTGC	21	63.3	TeIns_TID_9_Rv	CCGAGTGCAATGGGAGTGGGC	21	71.3	360
10	chr3R	11089486	11089562	roo	5.4	chr3R_15_11085001	76	TeIns_TID_10_Fw	TTTGAACACAATTTGGAAGTG	21	56.2	TeIns_TID_10_Rv	CAGTCCGCCCAAGAAACCAAA	21	66.5	359
11	chrX	7785488	7785556	hobo	3.3	chrX_10_7785001	68	TeIns_TID_11_Fw	AAGTTTTATGACCTCCATATG	21	51.1	TeIns_TID_11_Rv	CATTAATTTCGCCAGCACAGC	21	62.9	342
12	chrX	15391856	15391929	roo	4.1	chrX_13_15390001	73	TeIns_TID_12_Fw	TATATATTTCGGTAAAACCCTG	22	53.3	TeIns_TID_12_Rv	CTTTGCATGATAACATTAAGT	21	49.9	341

Table S2 Primer Lists

TIDAL and LnB shared sites

Site Number	Chr	Chr_coord _5p	Chr_coord _3p	TE	Coverage _Ratio	Tidal Insert Code	LnB765	TIDAL	CnT	TEMP	Space	FW primer name	FW primer sequence	Fw Length	Tm	RV primer name	RV primer sequence	Rv Length	Tm	Predicted Amplicon Size
1	chr2L	267478	267547	P-element	4.4	chr2L_18_	chr2L_265	chr2L_265		chr2L_2650	69	TeIns_LnB_1_Fw	ACATACACAGAGACAGGAAAGC	22	56.1	TeIns_LnB_1_Rv	CGAATTTATGGAATTCAGCATT	22	58.2	314
2	chr2L	1167146	1167232	opus	2.6	chr2L_21_	chr2L_116	chr2L_116	chr2L_1		86	TeIns_LnB_2_Fw	GTCCACTATAACGTTACCCAAC	22	55.3	TeIns_LnB_2_Rv	CAAGAGACCATTGCGCAGGT	20	63.6	386
3	chr2L	20800099	20800248	Burdock	5.8	chr2L_22_	chr2L_208	chr2L_208		chr2L_2080	149	TeIns_LnB_3_Fw	CCTCCTGTCCCTACCAACTGG	21	63.1	TeIns_LnB_3_Rv	GACCTCTCCTGTCTCTTATTT	21	51.8	329
4	chr2R	9380884	9380961	3S18	2.4	chr2R_16_	chr2R_938	chr2R_938	chr2R_9	chr2R_938	77	TeIns_LnB_4_Fw	GGGGAGAAAATGGGAGAAAGC	21	63.3	TeIns_LnB_4_Rv	CCAAGTCGAAAATGTTGCTTC	21	59.7	365
5	chr2R	9442941	9443015	412	3.9	chr2R_13_	chr2R_944	chr2R_944	chr2R_9		74	TeIns_LnB_5_Fw	TGTCAAGAATCTGAAGTTACG	21	53.1	TeIns_LnB_5_Rv	GATTCGTTTGTTTATGGTCCA	21	57.5	331
6	chr2R	11578561	11578638	blood	5.6	chr2R_14_	chr2R_115	chr2R_115	chr2R_1	chr2R_115	77	TeIns_LnB_6_Fw	CATTTTTGAGTGCAGACCCTAC	22	58.8	TeIns_LnB_6_Rv	TGTCAAGTGTATATTGCTTCT	21	50.1	372
7	chr3L	934796	934851	hobo	8.3	chr3L_15_	chr3L_930	chr3L_930			55	TeIns_LnB_7_Fw	ATACGGAATTCAAACTATGTG	21	52	TeIns_LnB_7_Rv	TGGATTCGCTATCAGGATGTG	21	61	364
8	chr3L	22566716	22566793	hobo	4.3	chr3L_32_	chr3L_225	chr3L_225			77	TeIns_LnB_8_Fw	TTGCACTGCTATTTGCAGAAG	21	59.3	TeIns_LnB_8_Rv	CAATCGTCTCCATTTTGTATG	21	55.7	367
9	chr3R	18968019	18968094	Burdock	3.8	chr3R_19_	chr3R_189	chr3R_189		chr3R_189	75	TeIns_LnB_9_Fw	AGAGTGTTATCTATGGGAGAG	21	50.2	TeIns_LnB_9_Rv	GTCCATTTGTTTGTCCGCTCAC	22	63.9	375
10	chr3R	30626323	30626403	412	5.2	chr3R_22_	chr3R_306	chr3R_306		chr3R_306	80	TeIns_LnB_10_Fw	GAAATCCAAATGCCCGTTTCATG	23	66.5	TeIns_LnB_10_Rv	CTAGATTAGTGGCCATATTAAAG	23	52.3	379
11	chrX	6487781	6487856	blood	4.3	chrX_18_6	chrX_6485	chrX_6485	chrX_64		75	TeIns_LnB_11_Fw	TTTCATTCAAAATGCTTATGC	21	55.6	TeIns_LnB_11_Rv	CATGTAATGCGTGCATACATG	21	58.5	375
12	chrX	21399401	21399469	pogo	3.6	chrX_12_2	chrX_2139	chrX_2139		chrX_2139	68	TeIns_LnB_12_Fw	CGTGGAGGTAAGTGAAATTTC	21	56.4	TeIns_LnB_12_Rv	CGATGCTGCAACAAAACAAAT	21	61	321

Table S2 Primer Lists

CnT Only sites

Site Number	Insert_code	Chr	Start	Stop	Space	FW primer name	FW primer sequence	Fw Length	Tm	RV primer name	RV primer sequence	Rv Length	Tm	Predicted Amplicon Size
1	chrX_4330001	chrX	4334105	4334327	222	TeIns_CnT_1_Fw	CTAGGCTACTCCTAAAAGATATC	23	50.8	TeIns_CnT_1_Rv	ACGAGGCTAAAATAAAGGCCC	21	60.8	376
2	chrX_14000001	chrX	14001033	14007159	6126	TeIns_CnT_2_Fw	AACAAGAGAGAATGCTATAGTCG	23	54.8	TeIns_CnT_2_Rv	GATGAATTTTCGCAATTCACA	21	58.6	6092
3	chr3R_7695001	chr3R	7696150	7703338	7188	TeIns_CnT_3_Fw	CCACACTTATATTA ATTGGCA	22	53.1	TeIns_CnT_3_Rv	GATCTCTCGCCGCTGTCACTG	21	65.8	7171
4	chr3R_14020001	chr3R	14021702	14023463	1761	TeIns_CnT_4_Fw	CAGTTTGTCAAGAAACTGTTTAC	23	53.6	TeIns_CnT_4_Rv	CAGTTTGTCAAGAAACTGTTT	21	52.2	1734
5	chr3L_4360001	chr3L	4361048	4361901	853	TeIns_CnT_5_Fw	AGCTCAAAGAAGCTGGGGTCG	21	65.2	TeIns_CnT_5_Rv	CCATGAAATGAAACAATGATTAA	23	56.7	844
6	chr3L_17180001	chr3L	17180645	17180660	15	TeIns_CnT_6_Fw	ACGATTTTGCCGCTCACGC	19	66.8	TeIns_CnT_6_Rv	GCCGAAATTTGTAGTTAAAAT	21	52.2	394
7	chr2R_6945001	chr2R	6948454	6948972	518	TeIns_CnT_7_Fw	TCTAAAGTCGTGTTCCCTGAC	21	56.9	TeIns_CnT_7_Rv	CGTAAATTAAATTGGTTGAGGA	22	56	493
8	chr2R_13060001	chr2R	13064762	13064861	99	TeIns_CnT_8_Fw	TATATTTATGCCAGTCATGGG	21	55.2	TeIns_CnT_8_Rv	CGAGCAAGAATGTACCTCAAGT	22	58.5	357
9	chr2L_1805001	chr2L	1809645	1810122	477	TeIns_CnT_9_Fw	TTCAGGATTAGTTTGTCTAACAA	23	53.6	TeIns_CnT_9_Rv	ATTCCGAGAAATTTATTCCGA	21	57.7	447
10	chr2L_9780001	chr2L	9783398	9783415	17	TeIns_CnT_10_Fw	ATACGGGGTGCTGGTAAACTCC	22	63.4	TeIns_CnT_10_Rv	CGTCTTTGTCAACGAATCAATTG	23	61.7	394
11	chr4_180001	chr4	180927	183823	2896	TeIns_CnT_11_Fw	CTTTCTTATACGCATACATTG	21	50.6	TeIns_CnT_11_Rv	CGGCCATACACATTGGTCTTG	21	63.5	2892
12	chr4_1000001	chr4	999517	1001425	1908	TeIns_CnT_12_Fw	GTTCGTATTTACACTAGCTAAGG	23	52.4	TeIns_CnT_12_Rv	GTTACTCAGCAAAACGTAGTG	21	52.9	2008

Table S2 Primer Lists

TEMP Only Sites

Site Number	Chr	Start	Stop	rounder	InsertCode	Space	FW primer name	FW primer sequence	Fw Length	Tm	RV primer name	RV primer sequence	Rv Length	Tm	Predicted Amplicon Size
1	chr2L	9850436	9850443	9850001	chr2L_9850001	7	TeIns_TEM_1_Fw	GGTTGTCGTTGTCCTTCCTG	20	60.5	TeIns_TEM_1_Rv	GGTTCAGGTCAGGCAGGCAG	20	62	383
2	chr2L	1558495	1558995	1555001	chr2L_1555001	500	TeIns_TEM_2_Fw	CAGACAAAAGTAATGTGAAAC	21	50	TeIns_TEM_2_Rv	CAACCATTAATTGCTATATCA	21	50.7	482
3	chr2R	5334166	5334666	5330001	chr2R_5330001	500	TeIns_TEM_3_Fw	CCGTGTAGGCTTACAAGCAATAG	23	60.2	TeIns_TEM_3_Rv	CTTGTGGTGCAAAGTCAAATG	21	59.2	494
4	chr2R	2095477	2095485	2095001	chr2R_2095001	8	TeIns_TEM_4_Fw	GTCGACTATGTATGGAGGCTGCG	23	65.2	TeIns_TEM_4_Rv	CTTACAATTAGATTTAATGCTTT	23	50	405
5	chr3L	9571935	9572435	9570001	chr3L_9570001	500	TeIns_TEM_5_Fw	TCCGCAAATGTAAGGTATAGTG	22	57	TeIns_TEM_5_Rv	GTTTCGATGGATACGAAAACA	21	57.6	482
6	chr3L	5056174	5056674	5055001	chr3L_5055001	500	TeIns_TEM_6_Fw	CAATCCGCACTCAGACACTCAC	22	63.2	TeIns_TEM_6_Rv	GGCTCTAATCGTTTAGTTATAAA	25	52.1	499
7	chr3R	5035704	5035739	5035001	chr3R_5035001	35	TeIns_TEM_7_Fw	TGAATTTTACATTAAAGGCGG	21	57	TeIns_TEM_7_Rv	AATCCGAACCCGAATCGGAA	20	65.8	296
8	chr3R	1211275	1211285	1210001	chr3R_1210001	10	TeIns_TEM_8_Fw	TAGAACTATAGATCTAGCTAAGGG	24	50.8	TeIns_TEM_8_Rv	GTGATTGCCTTAGTTTTGTTT	21	53.8	398
9	chrX	294866	294869	290001	chrX_290001	3	TeIns_TEM_9_Fw	ATTGTATGCATAATTTACCACTT	23	52.6	TeIns_TEM_9_Rv	CGCATTCAGCTTCGGGTCGAC	21	61.8	395
10	chrX	5410656	5411156	5410001	chrX_5410001	500	TeIns_TEM_10_Fw	GTTAATAGTAATTAGCACATTGCA	24	53.5	TeIns_TEM_10_Rv	GAATCTCCGATATCATCAACGA	22	59	488
11	chrY	203747	204247	200001	chrY_200001	500	TeIns_TEM_11_Fw	CGTATCAGACAATCTAGTAGCAG	23	53.8	TeIns_TEM_11_Rv	GGACAACACCATTTCATAAT	20	52.3	487
12	chrY	1565254	1565753	1565001	chrY_1565001	499	TeIns_TEM_12_Fw	TCATCAAATCGGAGACACTCC	21	60.1	TeIns_TEM_12_Rv	ATGAATTTGTTCTATTTGAACTC	23	52.2	645

Primers for TE InDels In the ISO1-BL Line

TE Insertions

Site Number	Chr	Chr_coord_5p	Chr_coord_3p	TE	Coverage_Ratio		FW primer name	FW primer sequence	Fw Length	Tm		RV primer name	RV primer sequence	Rv Length	Tm	Amplicon Size
1	chr2R	2E+07	19768307	copia	4.5		ISO1_BL_FI1	TGCCTAGAATTTGCTATGCCGC	22	65.5		ISO1_BL_RI1	GTTTAAGTAAGGATCGATTTTAG	23	51.5	295bp
2	chr3L	4390337	4390468	roo	4.5		ISO1_BL_FI2	GTTGGACAAAGGAATCGGTAATG	23	61.8		ISO1_BL_RI2	GCATTCTGTACTGTTCCCAGCA	22	62.5	330bp
3	chr2R	8528862	8528983	I-element	4.4		ISO1_BL_FI3	CCATCAAAACGCACATATTGC	21	61.3		ISO1_BL_RI3	TTTCCGCTTTCCCACTTTC	19	60.1	273bp
4	chr3R	3E+07	29777247	F-element	17.9		ISO1_BL_FI4	TGGCGCTACGCTAATGAAATC	21	62.4		ISO1_BL_RI4	GCGGTCCTGATAAACGTATTT	21	58.2	414bp
5	chr2L	1068653	1068778	FB	4.2		ISO1_BL_FI5	CTGATAAGCGGCAGCATAAGCAG	23	65.3		ISO1_BL_RI5	GAGCCAAGCTTAGGCTGCAATT	22	64.1	517bp
6	chrX	6542904	6543018	I-element	4.3		ISO1_BL_FI6	TTGAAGTTGGCCATTTACGGG	21	64.1		ISO1_BL_RI6	GGACGCCAATGGAGCTTATTG	21	63.9	501bp
7	chrX	1.9E+07	19018748	copia	4.7		ISO1_BL_FI7	AAGATTTAGAGACACTCCACC	21	52.6		ISO1_BL_RI7	GCTTAAGGCCGCCGAACAA	19	65.6	469bp
8	chr3R	5066592	5066727	Tirant	4.4		ISO1_BL_FI8	TTATTACCACAGCTAAGAGTC	21	49.1		ISO1_BL_RI8	GATATGCTAATTCGGCGCG	19	61.6	515bp
9	chrX	1.1E+07	10556301	Doc	5.7		ISO1_BL_FI9	AGCCAAGATCTGTCATTTGTC	21	56.8		ISO1_BL_RI9	CAAACGACACAGCGAAGCGTA	21	64.6	363bp
10	chr2L	5999659	5999785	hobo	5.6		ISO1_BL_FI10	GTCGAAGTGCCGGAAACGT	19	63.5		ISO1_BL_RI10	CAAATTCTTAAAGCCAAGGCTT	22	59	479bp
11	chr2R	2.3E+07	22786444	hobo	5.3		ISO1_BL_FI11	AAGAATTTCTTTTCCCAAGCTC	22	58.1		ISO1_BL_RI11	GGTATACTTACCTTTTATGCAG	22	50.6	523bp
12	chr2L	9485261	9485390	hobo	4.2		ISO1_BL_FI12	GAGTGTCGCAGAGTCCGTCTG	21	63.6		ISO1_BL_RI12	CTAGTAGAATGATTTTGGGGCAA	23	59.5	514bp

TE Depletions

Site Number	Chr	Chr_coord_5p	Chr_coord_3p	TE	Coverage_Ratio		FW primer name	FW primer sequence	Fw Length	Tm		RV primer name	RV primer sequence	Rv Length	Tm	Predicted Amplicon Size		RV primer name	RV primer sequence	Rv Length	Tm	Predicted Amplicon Size
1	chrX	6335185	6335241	Tirant	4.6		ISO1_BL_FD1	GCTGATTTACGAATCACTTGAAG	23	58.1		ISO1_BL_RD1	GGCTAGCTGGGGACAATGTTG	21	64	380bp		ISO1_BL_RD1.1	CGTCATTGTATCGCGCTTATA	21	58.8	2757b.p.
2	chr3R	5045818	5045876	HMS-Beagle	4.4		ISO1_BL_FD2	GGTTACACAAGAGCGTGCAGC	21	63.2		ISO1_BL_RD2	CTAAGTCCCTAGCAATCAAGTGA	23	57.7	448bp		ISO1_BL_RD2.1	GCCACTTATTTATAAATGTCA	22	50	7393
3	chr3R	3.2E+07	31691562	Doc	4.3		ISO1_BL_FD3	AAGAGCGACTGAATAACGAAG	21	56.4		ISO1_BL_RD3	CCAGAACAATATGGAATAATT	21	51.1	449bp		ISO1_BL_RD3.1	GTCCATATCTGCTGCCACGC	20	64	5068
4	chr3R	1.7E+07	16756346	diver	3.6		ISO1_BL_FD4	CAATCAAACAGCGCGGCAC	19	64.1		ISO1_BL_RD4	CTGAACTGTATTCTTTCTTTCGC	23	57.4	436 bp		ISO1_BL_RD4.1	GGCCACCTCGGAGTGGCTC	19	65	6493
5	chr2L	1.3E+07	12861728	hobo	3.5		ISO1_BL_FD5	ATTTATCTTATCCTGTTATCCCA	24	53.9		ISO1_BL_RD5	CGACGCAAAACACCGTATTGA	21	64.1	458bp		ISO1_BL_RD5.1	TACTCCAGAATGTGCGTGGAG	21	61.2	1720
6	chr2L	1.8E+07	17951978	hobo	3.5		ISO1_BL_FD6	TAGAGCTAAGCCAGCCAGGAC	21	61		ISO1_BL_RD6	CACTCGAGTATTTTGTGTTGCC	22	59.7	381bp		ISO1_BL_RD6.1	AACTGTTACGAGATGATACAG	21	50	1785
7	chr3L	2927813	2927871	Tirant	3.5		ISO1_BL_FD7	GTCAGAGATAAAGAAGAAACTAA	23	50.5		ISO1_BL_RD7	GACTCGAGTAGCCACTCTCTG	21	55.9	393bp		ISO1_BL_RD7.1	TGTTGTTTTGGTGGTGTATACAA	23	57.9	8527
8	chrX	1.2E+07	11633445	297	2.9		ISO1_BL_FD8B	AACACAGATCAGTGTGTGAGT	21	52.8		ISO1_BL_RD8B	TCTCCTCAATCCAATTTGCAT	21	59.5	430bp		ISO1_BL_RD8.1	CAGAATGGAAATAAATTTGTT	21	51.4	7371
9	chr3L	8481746	8481802	roo	3.3		ISO1_BL_FD9B	AGTAGCTGTGGATCTGTGGC	20	57.9		ISO1_BL_RD9B	ATTTACTTAGGCCTCTGCGTA	21	56	408bp		ISO1_BL_RD9.1	TTGCGTTCTGCATCTGTCAGA	21	63.1	9454
10	chr2R	7608382	7608431	I-element	2.6		ISO1_BL_FD10B	ATTCAAGCGTATTGTTTATTG	21	52.4		ISO1_BL_RD10B	CATAACAAGCCAGCAATTAGTT	22	56.3	317bp		ISO1_BL_RD10.1	TGATCGAAATGGTGTTAAAGTCG	23	61.2	5489
11	chr2R	8398189	8398240	Doc	3		ISO1_BL_FD11B	TCGGAGACGGTGGATAGGTAG	21	61.4		ISO1_BL_RD11B	GTAGGTGCACTTGGAGCACC	20	57.2	399bp		ISO1_BL_RD11.1	GAGGCACGAACTGCTGGCT	19	65	5061
12	chr3R	3.1E+07	31279405	diver	2.5		ISO1_BL_FD12B	AGAGATCCAAATTCAAATATG	21	51.1		ISO1_BL_RD12B	ATCACGTCTGATCGCTATGAC	21	57.8	301bp		ISO1_BL_RD12.1	CTTGGCTAAAACCCACACAC	20	58.1	6402
